# Effect of a Probiotic and a Synbiotic on Body Fat Mass, Body Weight and Traits of Metabolic Syndrome in Individuals with Abdominal Overweight: A Human, Double-Blind, Randomised, Controlled Clinical Study

**DOI:** 10.3390/nu15133039

**Published:** 2023-07-05

**Authors:** Christiane Laue, Ekaterina Papazova, Angelika Pannenbeckers, Jürgen Schrezenmeir

**Affiliations:** 1 Clinical Research Center Kiel, Kiel Center of Innovation and Technology, Schauenburgstraße 116, D-24118 Kiel, Germany; c.laue@crc-kiel.de (C.L.); e.papazova@crc-kiel.de (E.P.); a.pannenbeckers@crc-kiel.de (A.P.); 2 University Medicine, Johannes-Gutenberg University, D-55131 Mainz, Germany

**Keywords:** probiotics, synbiotics, *L. fermentum*, weight management, body fat mass, metabolic syndrome, NAFLD

## Abstract

*L. fermentum* strains K7-Lb1, K8-Lb1 and K11-Lb3 were found to suppress Th1 and Th2 response and to enhance defensin release by enterocytes, respectively. Based on these anti-inflammatory actions, we investigated the effect of these strains on traits of metabolic syndrome, which is driven by low-grade inflammation. In a double-blind, randomised, placebo-controlled clinical trial with three parallel arms, 180 individuals with abdominal overweight were administered for 3 months with (1) placebo; (2) probiotic, comprising *L. fermentum* strains; or (3) synbiotic, comprising the strains + acacia gum (10 g daily). The effects were evaluated using Kruskal–Wallis one-way analysis of variance on ranks and post hoc tests (Holm–Sidak and Dunn’s tests). The alteration (∆) in body fat mass (kg) (primary parameter) during intervention was significantly (*p* = 0.039) more pronounced in the Probiotic group (−0.61 ± 1.94; mean ± SD) compared with the Placebo group (+0.13 ± 1.64). Accordingly, differences were found in ∆ body weight (*p* = 0.012), BMI (*p* = 0.011), waist circumference (*p* = 0.03), waist-to-height ratio (*p* = 0.033), visceral adipose tissue (SAD) (*p* < 0.001) and liver steatosis grade (LSG) (*p* < 0.001), as assessed using sonography. In the Synbiotic group, ∆SAD (*p* = 0.002), ∆LSG (*p* < 0.001) and ∆constipation score (*p* = 0.009) were improved compared with Placebo. The probiotic mixture and the synbiotic improved the parameters associated with overweight.

## 1. Introduction

In recent years, numerous studies have provided evidence that the intestinal microbiota has a key role in the interface between dietary factors and host biology and that overweight, diabetes and liver steatosis, which are known to depend on dietary factors, are associated with alterations in the composition and diversity of the intestinal microbiota [1,2,3,4,5,6,7].

The effects of probiotics on glucose and lipid metabolism, body fat mass, weight, visceral adipose tissue and liver steatosis were shown in several meta-analyses on the total variety of probiotics [8,9,10,11,12,13,14,15,16]. Some probiotic species/strains, however, seem to be more efficacious [14], which may be due to species- and strain-specific properties, e.g., gastrointestinal transit, anti-inflammatory properties, defensin induction, barrier function, existing mannose pathway, bile salt metabolism, energy utilisation from indigestible polysaccharides, etc. [17,18,19,20,21,22].

The *Limosilactobacillus (L.) fermentum* strains used in this trial as probiotic were isolated from Kimere, a traditional fermented food prepared from pearl millet in the Mbeere community in Kenya, East Africa [17]. This probiotic is fermented for 18–24 h prior to consumption and is consumed in its active fermenting state. The strains were selected for their resistance towards bile salts and acidity [17] and for their anti-inflammatory properties. *L. fermentum* strains K7-Lb1 (DSM 22831) and K8-Lb1 (DSM 22832) were found to suppress the Th1 and Th2 response of PBMCs in vitro, as assessed according to IFNα and IL-4 secretion, while others increased Th1 and Th2 response [20]. In contrast to other investigated *L. fermentum* strains, strain K11-Lb3 (DSM 22838) induced the production of hBD-2 by CaCo-2 cells [19], which was shown to enhance gut barrier function [23]. Impaired gut barrier function and translocation of LPS leading to inflammation in the liver and adipose tissue are associated with high sucrose and animal fat intake and with obesity and other traits of metabolic syndrome, including non-alcoholic fatty liver disease [22].

*L. fermentum* has an existing mannitol pathway and is thus able to use fructose as electron acceptor and recover NAD by metabolizing fructose to mannitol and producing acetate instead of ethanol. In contrast, other heterofermentative lactobacilli, like *W. confusa,* need to keep the NADH/NAD ratio balanced, when ATP is generated for energy production from fructose metabolism with the conversion of xylulose-5-phosphate to lactate, by recovering NAD with the conversion of acetyl-phosphate to ethanol. In contrast to these species, *L. fermentum*, hence, does not produce ethanol when fed with fructose and can compete for this energy source with microbes that do not possess a mannitol pathway and metabolize fructose to ethanol [22,24].

Based on these properties, one might expect more pronounced effects of these strains on traits of metabolic syndrome than those effects found in meta-analyses on the whole variety of probiotics without discriminating species and strain specificity.

The combination of these three *L. fermentum* strains with the prebiotic acacia gum was expected to permit even more pronounced effects, since acacia gum was previously shown to increase not only the number of bifidobacteria but also the number of lactobacilli in the gut [25,26]. Acacia gum, hence, is supposed to promote their propagation and their effects in the strict sense of a synbiotic [27]. A dosage of 10 g/day acacia gum was demonstrated to be sufficient for enhancing faecal lactobacilli and bifidobacteria [25,26].

Prebiotics increase satiety [28], reduce body weight, BMI, body fat, postprandial and fasting glucose, glycated haemoglobin (HbA1c), insulin levels and fasting triglycerides; increase HDL-C; and decrease enzymatic markers of liver steatosis, according to meta-analyses of RCTs [29,30,31,32,33].

For acacia gum, effects on traits of metabolic syndrome were also described. Body mass index (BMI), body fat mass and the visceral adiposity index were reduced in randomised controlled trials (RCTs) by 0.32 to 0.57 kg/m^2^, 2.18%, and 0.75, respectively, during an intervention period of 6 to 12 weeks [34,35,36]. Even though the effects still need to be confirmed in more stringent double-blind RCTs, a mechanism for a beneficial effect on glucose metabolism was identified: the reduction in the sodium glucose transporter SGLT1 in the intestine [37,38].

The present DB-RCT aimed at providing evidence of an effect of a probiotic composed of these three *L. fermentum* strains and of this synbiotic on traits of metabolic syndrome for the first time. The target parameters were selected for obtaining a health claim according to the Health Claim Directive of the EU (Regulation (EC) No. 1924/2006 on Nutrition and Health Claims Made on Foods) and/or the Regulation (EU) No. 609/2013 for Food for Special Medical Purposes after having confirmative evidence. In particular, EFSA Guidance on the scientific requirements for health claims related to appetite ratings, weight management and blood glucose concentrations [39], and EFSA Guidance on the scientific requirements for health claims related to antioxidants, oxidative damage and cardiovascular health [40] were considered.

## 2. Materials and Methods

### 2.1. Ethics

The application for ethical approval including study protocol and informed consent form was reviewed by the Independent Ethics Committee of the Medical Association of Schleswig-Holstein on 30 May 2021. Ethical evaluation resulted in written stated favourable opinion, with reference number 063/21 (I), on 1 July 2021.

The study was conducted in line with the principles of the Declaration of Helsinki, in accordance with the guidelines for Good Clinical Practice (ICH-GCP) and in accordance with the study protocol (Study protocol_Slim-LfX-2020, version 1.0_17.03.2020; see Supplementary Materials). Written informed consent was obtained from the study participants prior to any study-specific process.

### 2.2. Study Design and Conduct

**Figure 1:**
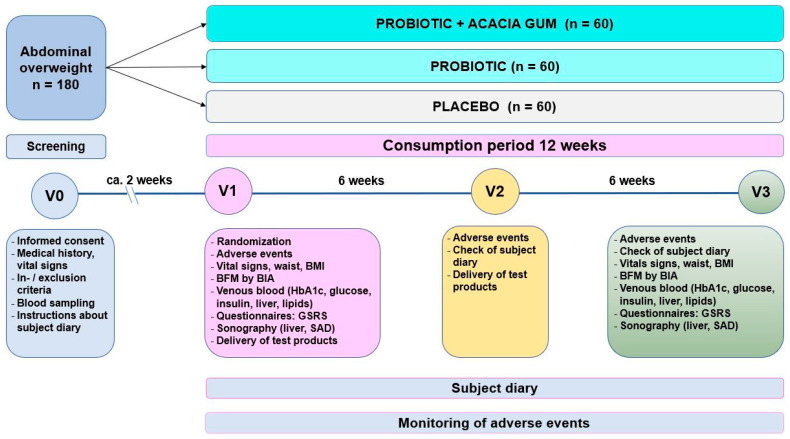
Summary of the study design.

This study followed a double-blind, randomised, placebo-controlled design with three parallel arms (Figure 1). A total of 180 female and male overweight individuals aged ≥18 years complying with the inclusion and exclusion criteria listed below were enrolled in the study. Individuals were recruited from the database of the study site and with advertisements and flyers. The place of recruitment was Kiel and the surrounding area.

The investigators completed a subject screening log to document each individual screened for this study regardless of enrolment. This screening log also aimed at avoiding selection bias. Each individual was assigned to one of the three test products in randomised order. Individuals were supposed to appear for four visits in total during the study.

Visit 0 (screening visit) and the start of the first intervention scheduled for visit 1 (intervention 1) were supposed to take place within ca. 2 weeks to provide actual lab parameters (e.g., impaired fasting glucose). The subsequent visits, V2 and V3, followed visit 1 after 6 weeks and 12 weeks, respectively.

Study participants were supposed to appear after a 12 h overnight fasting period at all visits except V2. They were provided with a diary at V0, were advised to keep it until the end of the study and were asked to fill in the diary every day with regards to product consumption, concomitant medication and events with potential significance for the study. They were required to bring the diary back to the study site at every visit for inspection.

This monocentre trial was conducted at CRC Clinical Research Center Kiel/Germany between June 2021 (first patient, first visit) and May 2022 (last patient, last visit).

### 2.3. Subject Selection and Withdrawal

The study population consisting of female and male individuals with abdominal overweight were recruited from the CRC database with the announcement of the study sent by mail, and with advertisements in the local daily newspaper and flyers that were on display, e.g., at family doctors’ waiting rooms.

They contacted the study site by phone or email and were asked if they were interested in further information. During telephone contact, subjects received summarized information about the planned trial. In case the individual was interested in participation, he/she was provided with the written subject information by e-mail or regular mail. After the subjects had read the subject information, they contacted the study site to clarify any further questions and/or schedule a screening visit.

### 2.4. Inclusion Criteria

To be enrolled, the following criteria had to be fulfilled: (1) overweight or obese (BMI ≥ 25), (2) elevated waist circumference (>94 cm and >80 cm (for European men and women, respectively)), (3) age ≥ 18 and (4) written informed consent.

### 2.5. Exclusion Criteria

Any of the following was regarded as a criterion for exclusion from enrolment into the study: subjects currently enrolled in another clinical study; subjects having finished another clinical study within 4 weeks before inclusion; hypersensitivity, allergy or intolerance to any compound of the test products (e.g., acacia gum); previous implantation of a cardiac pacemaker or other active implants; sulfonylurea treatment; any disease or condition that might have significantly compromised the hepatic (ascites), hematopoietic, renal, endocrine, pulmonary, central nervous, cardiovascular, immunological, dermatological or gastrointestinal system, or any other body system, with the exception of the conditions defined by the inclusion criteria; history of or present liver deficiency as defined by Quick test result < 70%; regular medical treatment, including OTC, that may have impacted the study aims (e.g., probiotics containing supplements, laxatives, steroids, etc.); history of hepatitis B or C, or HIV; major cognitive or psychiatric disorders; subjects who were scheduled to undergo any diagnostic intervention or hospitalization that may have caused protocol deviations; simultaneous study participation of members of the same household; pregnancy and lactation; ascites as assessed with sonography; any diet to lose body weight; eating disorders or vegan diet; anorexic drugs; present drug abuse or alcoholism; legal incapacity.

### 2.6. Test Products

#### 2.6.1. Placebo

The main component of the placebo was microcrystalline cellulose (MC). MC is refined wood pulp, a connective agent added to prescription drugs, over-the-counter medications and dietary supplements. It is a white, free-flowing powder. Chemically, it is an inert substance, is not degraded during digestion and has no appreciable absorption. In large quantities, it provides dietary bulk and may lead to a laxative effect. The MC used for the placebo in this study was provided by NUTRILINEA S.r.l., Italy, a company certified for the design and production of food supplements. The placebo contained the same amount of the components maltodextrin, sucralose and cream flavour as the probiotic and synbiotic test products (Table 1). Dietary fibre content was also identical in weight (Table 1). According to this, all study test products were similar in smell, flavour, colour, texture and appearance.

#### 2.6.2. Probiotic Test Product

The probiotic test product contained three *L. fermentum* strains: K7-Lb1 (DSM 22831), K8-Lb1 (DSM 22832) and K11-Lb3 (DSM 22838) [17,19,20].

The strains were produced by CSL (**C**ENTRO **S**PERIMENTALE DEL **L**ATTE S.R.L., I-26839 Zelo Buon Persico (LO), Strada per Merlino 3, Italy), certified for the production of bacterial freeze-dried cultures for food and farming/livestock, and pharmaceutical and nutraceutical sectors. The company CSL has been assessed and complies with the requirements of FSSC 22000 (certification scheme for food safety systems).

The species *L. fermentum* is included in EFSA’s **Q**uality **P**resumption of **S**afety (QPS) list. The strains were shown to be sensitive to antibiotics, following EFSA guidelines and ISO/IDF standards [41,42]. The content of each strain per sachet at production was 5 × 10^9^ CFU (Table 1). The target CFU per strain and sachet at the time of consumption was ≥1 × 10^9^ CFU. Daily intake was intended to be ≥2 × 10^9^ CFU for each strain at the end of shelf life. An assessment 20 days after the last visit of the last individual showed that the daily dose was 5.46 × 10^9^ CFU for the probiotic mixture.

#### 2.6.3. Synbiotic Test Product

The synbiotic test product contained the strains *Lactobacillus fermentum* K7-Lb1, *L. fermentum* K8-Lb1 and *L. fermentum* K11-Lb3 [17,19,20], and acacia gum (gum arabic). Acacia gum is purified and instantized soluble dietary fibre and was produced by NEXIRA (129 Chemin de Croisset—CS94151—76723 Rouen Cedex, France). It is a natural dietary fibre of the product line FIBREGUM ^TM^ (FIBREGUM P). To ensure characteristics identical to those of the placebo, sucralose, cream flavour and maltodextrin were also added. The content of each strain per sachet at production was 5 × 10^9^ CFU (Table 1). The target CFU per strain and sachet at the time of consumption was ≥1 × 10^9^ CFU. Daily intake was intended to be ≥2 × 10^9^ CFU for each strain at the end of shelf life. An assessment 20 days after the last visit of the last individual showed that the daily dose was 1.97 × 10^9^ CFU.

All study test products were similar in smell, flavour, colour, texture and appearance.

#### 2.6.4. Mode of Consumption

Each study participant was instructed to consume one sachet of 6 g of test product twice daily, in the morning and evening.

### 2.7. Assessments

#### 2.7.1. Bioelectrical Impedance Analysis (BIA)

BIA was used for the assessment of body composition, in particular body fat mass (BFM) (primary parameter), lean body mass (LBM) and visceral fat mass (VAT_BIA_) (L) (using an algorithm taking waist circumference and body fat into account). In this study, SECA mBCA 515 (medical Body Composition Analyser; seca GmbH & Co. KG, Hamburg, Germany) was used.

#### 2.7.2. Visceral Adiposity Index (VAI)

The VAI (secondary parameter) was assessed according to Amato et al., 2010 [43]. The VAI is an index composed of waist circumference (WC) (cm), triglycerides (TGs) (mmol/L) and HDL-C (mmol/L), and calculated in males as VAI = (WC/(39.68 + (1.88 × BMI)) × (TG/1.03) × (1.31/HDL) and in females as VAI = WC/(36.58 + (1.89 × BMI)) × (TG/0.81) × (1.52/HDL-C), respectively.

#### 2.7.3. Anthropometry

Body weight was assessed using an electronic scale (seca 704; seca GmbH & Co. KG, Hamburg, Germany). Height was assessed using a stadiometer (seca 217; seca GmbH & Co. KG, Hamburg, Germany). According to the guideline of the World Health Organisation, waist circumference was measured at the midpoint between the lower margin of the least palpable rib and the top of the iliac crest, using stretch-resistant tape [44].

#### 2.7.4. Blood Pressure

Blood pressure was measured using an aneroid sphygmomanometer (ERKA. Kallmeyer Medizintechnik GmbH & Co. KG, Bad Tölz, Germany) at screening, before ingestion of the test product and 120 min after the ingestion of the test product.

#### 2.7.5. Serum Parameters

All laboratory parameters were determined at the certified Laboratory Dr. Krause and Colleagues, MVZ GmbH, Kiel.

#### 2.7.6. Sonography

Visceral adipose tissue was assessed with sonography according to Armellini et al., 1991 [45], using Mindray DC-T6 (Mindray Healthcare GmbH, Darmstadt, Germany). Briefly, the sagittal abdominal diameter (SAD) between the dorsal surface of the rectus muscle and the anterior wall of the aorta was used as measure for visceral fat.

The liver steatosis grade was assessed with sonography. Quantification followed the criteria (liver echogenicity, posterior beam attenuation and loss of echoes from the walls of the portal veins) given by Saverymuttu et al., 1986 [46], resulting in the grading 0 = no steatosis; 1 = slight steatosis; 2 = moderate steatosis; and 3 = severe steatosis.

Ascites was excluded with the examination of potential spaces (including hepatic recesses and around the peripheral hepatic borders; splenic recesses and around the peripheral splenic borders; right sub-phrenic space; left sub-phrenic space; and sub-hepatic space (Morrison’s pouch)) according to Alnumeiri et al., 2015 [47].

#### 2.7.7. Medication

Anti-diabetic medication was quantified by expressing the percentage of the maximal dose per day $(X1*Dose_{X1\_max}$ of metformin).

Anti-hypertensive medication ($X2*Dose_{X2\_max}+Y2*Dose_{Y2\_max}+\ldots$) and anti-lipidemic medication ($X3*Dose_{X3\_max}+Y3*Dose_{Y3\_max}+\ldots$) (*Xi* < 1, *Yi* < 1) were quantified assuming that the maximal daily dose for each drug was 100%.

#### 2.7.8. Gastrointestinal Symptoms

Gastrointestinal symptoms were quantified with the Gastrointestinal Symptom Rating Scale (GSRS; according to Svedlund 1988, Dimenäs 1995 and Revicki 1998 [48,49,50]) related to the last 7 days before visits V1, V2 and V3.

#### 2.7.9. Compliance

Compliance was assessed (1) by counting consumed test products at V3 and the end of the study, respectively, and (2) using the Morisky score [51].

#### 2.7.10. Adverse Events (AEs)

An adverse event was defined as the appearance or worsening of any undesirable sign, symptom or medical condition occurring in an individual event if the event was not considered to be related to study products. Medical conditions/diseases present before starting administration of the study products were only considered adverse events if they worsened after starting administration of the study products. Abnormal laboratory values or test results were regarded as adverse events only if they induced clinical signs or symptoms and if they were considered clinically significant or required therapy.

A severe adverse event (SAE) was defined as an event that was fatal or life-threatening, resulted in persistent or significant disability/incapacity, constituted a congenital anomaly/birth defect, or required inpatient hospitalization or prolongation of existing hospitalization, unless hospitalization was due to one of the following: routine treatment or monitoring of the studied indication, not associated with any deterioration in condition; elective or pre-planned treatment for a pre-existing condition that was unrelated to the indication under study and had not worsened since the start of the administration of the study products; treatment on an emergency outpatient basis for an event not fulfilling any of the definitions of an SAE given above and not resulting in hospital admission; social reasons and respite care in the absence of any deterioration in the patient’s general condition; it was medically significant, i.e., defined as an event that jeopardizes the individual or may require medical or surgical intervention to prevent one of the outcomes listed above.

The investigator determined the relationship of the AE and test product consumption using the following scale:

- Not related: No investigational product was taken, or the AE could be ascribed with reasonable certainty to another cause.
- Unlikely: There were good reasons to think that there was no relationship.
- Possible: Equally valid arguments could be considered for or against an implication of the study product.
- Probable: The relationship was likely.
- Certain (definitely): There was strong relationship.

### 2.8. Statistics

#### 2.8.1. Determination of Sample Size

The primary parameter, body fat mass, was selected by estimating the sample size for several potential parameters (HbA1c, HOMA-IR, body weight, body fat mass, waist, etc.) within different populations (overweight, obesity, type 2 diabetes, IFG, etc.) based on the most recent meta-analysis of the effects of probiotics (all species and strains) published by Koutnikova et al., 2019 [14]. Since we expected a more pronounced effect by the selected strains to be used in this study and the combination with acacia gum, we assumed a twofold higher effect size (measured with Cohen’s d) than that found for the whole variety of probiotics. Taking this into account, the target parameter with the lowest estimated sample size was body fat mass in individuals with overweight (N = 56 for each arm). Accordingly, this target was defined as the primary parameter. Assuming a dropout rate of maximally 7%, a target number of *n* = 60 subjects per group was determined.

#### 2.8.2. Definition of Sets to Be Analysed

##### Full Analysis Set (FAS)

Compliance with the ITT principle would necessitate complete follow-up of all randomised subjects for study outcomes. As this cannot be achieved in most studies, a full analysis set (FAS) was planned to be analysed for providing evidence of an effect. This is as complete as possible and as close as possible to the ITT set (FAS) including all randomised test persons. The elimination of individuals was considered to be justified according to the ICH E9 guideline in the following cases:

- Violation of an essential and, before randomization, objectively measurable inclusion criterion.
- Not taking a single dose of the test substance (without knowledge of the assigned test group).
- Lack of any dates for the assessment of effectiveness after randomization.

##### Intention-To-Treat (ITT) Set

Evaluation in the ITT and PP populations served as sensitivity analysis with regard to the primary and secondary parameters. ITT was defined as all individuals randomised and having taken at least one dose of the test products (at V1). Subjects were evaluated in the planned treatment regimen rather than the actual treatment given.

##### Per-Protocol (PP) Set

The PP set was defined as all randomised individuals who had no major protocol deviation.

Per-protocol analysis was used for checking the robustness of the product effect.

#### 2.8.3. Statistical Tests

Null hypothesis

In case of normal distribution, the null hypothesis was the following: There is no difference among the mean values of the Probiotic, Synbiotic and Placebo (control) groups concerning the primary, secondary or explorative parameters to be tested acc. to the parameter description in the study protocol. If data are not normally distributed, the null hypothesis assumes an equality of medians of the three groups concerning the parameters to be tested.

Alternative hypothesis

In case of normal distribution, at least one of three mean values differs from the mean value of another group concerning the primary, secondary or explorative parameters to be tested acc. to the parameter description in the study protocol. If data are not normally distributed, at least one median differs from another median concerning the parameters to be tested.

Testing of Normality

All metric data were tested for normal distribution using the Shapiro–Wilk test at the 2.5% level with the following null hypothesis: data are normally distributed. If *p* > 0.025, the null hypothesis was confirmed, and the data were considered normally distributed. The test for normality failed if *p* ≤ 0.025.

Homoscedasticity was investigated with the Brown–Forsythe equal variance test at the 2.5% level.

Tests for Comparison of the Three Groups

In case of normality and homoscedasticity, the parametric method one-way ANOVA was used for statistical analysis for comparing the three groups according to the 3-arm design at the significance level of 5%. Otherwise, Kruskal–Wallis ANOVA on ranks was performed.

Post Hoc Tests

Post hoc tests were performed at the overall significance level of 5% if the main ANOVA test (parametric or non-parametric) was significant. The Holm–Sidak method was used in case of normality, and the Dunn’s method was the choice if the normality test failed, respectively.

Post hoc tests were performed focusing on comparisons versus the Placebo group only, based on the fact that this was the major focus for assessing efficacy.

Descriptive Statistics

Regarding descriptive statistics, data were expressed as means, standard deviations (SDs), medians, 25th and 75th percentiles, and frequency of observation. In figure means ± SEMs are used for depicting the results.

#### 2.8.4. Definition of Primary, Secondary and Exploratory Data

Body fat mass (BFM), as assessed with bioelectrical impedance analysis (BIA), was defined as the primary parameter, and the visceral adiposity index (VAI) according to Amato et al., 2010 [43], as the secondary parameter. All other parameters were regarded as exploratory.

#### 2.8.5. Data Screening and Transformation

In the analyses of primary and secondary parameters, a significant deviation from the normal distribution was examined. Additional investigations were conducted to find the skewness as a measure of how symmetrically the observed values were distributed about the mean. Some transformations to logarithmic scales were applied to these parameters on the basis of the skewness sign in order to avoid data loss due to rank order in non-parametric tests and to achieve normal distribution if possible.

Furthermore, an attempt was made to use an additional approach to evaluate the change between the start and the end of the study. Instead of the evaluation of ∆(V3-V1), ANCOVA with values at V3 as the dependent variable and baseline (V1) values as the covariate was performed. The aim was to compare the real study effect at the study end with exclusion of the baseline values’ influence. This was possible, e.g., in the analysis of the absolute body fat mass (BFM; kg), because the prerequisites for normality and homoscedasticity were given.

#### 2.8.6. Approach to Treatment of Missing Values

The missing values of primary and secondary parameters were subjected to analysis in order to assess the robustness of the primary analysis. First, we examined whether missing values in the primary and secondary parameters were likely to be MCAR (missing completely at random). Second, the distribution of missing values in the three groups was compared using the chi-square test at the 5% significance level.

#### 2.8.7. Database Generation and Management

The database for statistical analysis was generated as follows:

- The database was compiled after the last test person had completed the study.
- Data from paper documentation (CRFs, questionnaires) were transferred to electronic files by two persons each (double data entry), and the files were compared for discrepancies.
- Discrepancies between entries were corrected.

Then, the data bank was closed, and the data were transformed into an analysable form ready for statistical evaluation and unblinded.

#### 2.8.8. Interim Analysis

Interim analysis was neither planned nor performed.

#### 2.8.9. Software

SigmaPlot 14.0 (Systat Software, Inc., Frankfurt, Germany) was the main statistical programming software for analysing this study.

## 3. Results

### 3.1. Key Data of Study Conduct

**Figure 2:**
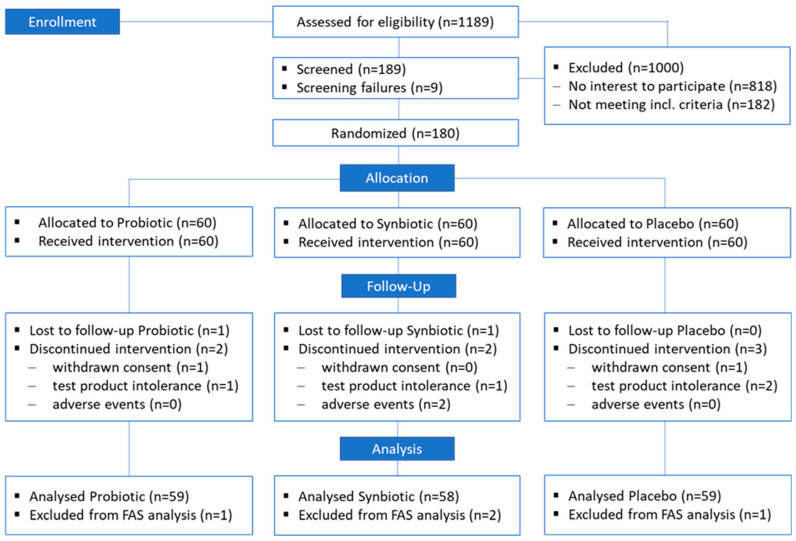
Flow diagram of the study progress through the different phases (according to CONSORT).

During the recruitment process, which included contacting individuals from the CRC database and using advertisements and flyers, 1189 subjects were addressed, and those interested were invited to screening visit V0; see Figure 2. In total, *n* = 189 individuals were screened by the investigator, and 9 (*n* = 9) individuals were screening failures due to laboratory values (*n* = 4), withdrawal of consent (*n* = 4) and current antibiotic therapy (*n* = 1). As intended, *n* = 180 individuals could be randomised and allocated to three groups—Probiotic, Symbiotic and Placebo, with 60 individuals each.

Ten individuals dropped out during the study (Probiotic: *n* = 3; Synbiotic: *n* = 4; and Placebo: *n* = 3), where two (*n* = 2) withdrew their consent (Probiotic: *n* = 1; Placebo: *n* = 1), four (*n* = 4) individuals reported an intolerance to the test product (Probiotic: *n* = 1; Synbiotic: *n* = 1; and Placebo: *n* = 2), two (*n* = 2) were lost to follow-up (Probiotic: *n* = 1; Synbiotic: *n* = 1) and two (*n* = 2) were lost due to an adverse event (Synbiotic: *n* = 2).

Four individuals were excluded from the FAS evaluation (Probiotic: *n* = 1; Synbiotic: *n* = 2; and Placebo: *n* = 1) due to lack of data for the assessment of an effect.

### 3.2. Study Populations

#### 3.2.1. ITT Population

The ITT population in this study consisted of all 180 randomised study participants (66 men and 114 women), with 60 being assigned to Probiotic; 60, to Synbiotic; and 60, to the Placebo (control) group.

#### 3.2.2. FAS Population

Six of the ten individuals who discontinued intervention (dropped out) came to an end-of-study visit, so BIA (bioelectrical impedance analysis) could be assessed. Because data for the primary parameter—alteration in body fat mass (BFM)—were thereby available, they became part of the FAS population. The other four (*n* = 4) individuals with only one BIA measurement were excluded from FAS analysis. Hence, the FAS population consisted of *n* = 176 individuals: *n* = 59 from the Probiotic group, *n* = 58 from the Synbiotic group and *n* = 59 from the Placebo group.

The primary analysis was performed based on this dataset.

#### 3.2.3. PP Population

All 10 individuals who dropped out were excluded from the PP population. Beside these dropouts, no major deviations from the study protocol occurred. So, the PP population consisted of 170 individuals: *n* = 57 from the Probiotic group, *n* = 56 from the Synbiotic group and *n* = 57 from the Placebo group.

### 3.3. Missing Values

The participants of the study were not aware of their BIA and blood results. Thus, discontinuation from the study due to knowledge about these values could be excluded. In other words, the reasons for withdrawal were unrelated to the outcomes of the primary and secondary parameters. Hence, missing values were regarded as completely at random (MCAR) and could be neglected without loss of validity of study findings.

The primary and secondary parameters were defined as the difference between the end and start of intervention. Individuals who had two BIA measurements were included in the FAS population regardless of the date of the second measurement. So, there were no missing values concerning the primary parameter in the FAS.

The visceral adiposity index (VAI), the secondary parameter, was calculated based on the values of the waist circumference (WC), triglycerides (TGs), body mass index (BMI) and high-density lipoprotein (HDL). Four of the six persons from the FAS population who withdrew preliminarily from the study did not have all measurements of these parameters at the study end. Their missing values constituted only 2.27% of all VAI values.

The three groups did not significantly differ in missing values in the FAS population acc. to the secondary parameter, the VAI (Chi^2^ (2, N = 180) = 0.499, *p* = 0.779) (chi-square-inaccurate because of the very small count of missing values: *n* = 2 in Probiotic, *n* = 1 in Synbiotic and *n* = 1 in the Placebo group). Hence, the missing values could be neglected and were not replaced with imputed values in the primary analysis. The statistical evaluation was performed based on the available case analysis. (Imputations were only made for ITT evaluation).

Other blood values for explorative parameter analysis were missing because of insufficient blood sample volume. Imputations were not made for these values either.

### 3.4. Baseline Characteristics of the FAS Population

The FAS population (*n* = 176) included 66 male and 114 female individuals. The baseline demographic and biometric data (Table 2) show that the population was characterized by abdominal overweight and traits associated with abdominal overweight.

The baseline characteristics at visit V0 and visit V1 (before intervention), respectively, did not significantly differ among the Placebo, Probiotic and Synbiotic groups (Table 3a). The laboratory parameters did not significantly differ either, with the exception of HDL-C and ALT (Table 3b).

### 3.5. Alterations during Intervention

#### 3.5.1. Body Composition as Assessed with Body Impedance Analysis (BIA) including Primary Parameter (Body Fat Mass (BFM))

The alteration during intervention ∆(V3-V1) in the primary parameter, BFM (kg), in the FAS population was normally distributed, with *p* = 0.437, as assessed using Shapiro–Wilk. ∆(V3-V1) in the relative BFM (%) was non-normally distributed, showing skewness to the left (negative). Data transformation, log_10_(Max—∆ BFM), did not lead to a normal distribution of the gathered relative BFM values, so the statistical evaluation was performed with Kruskal–Wallis ANOVA on ranks.

**Figure 3:**
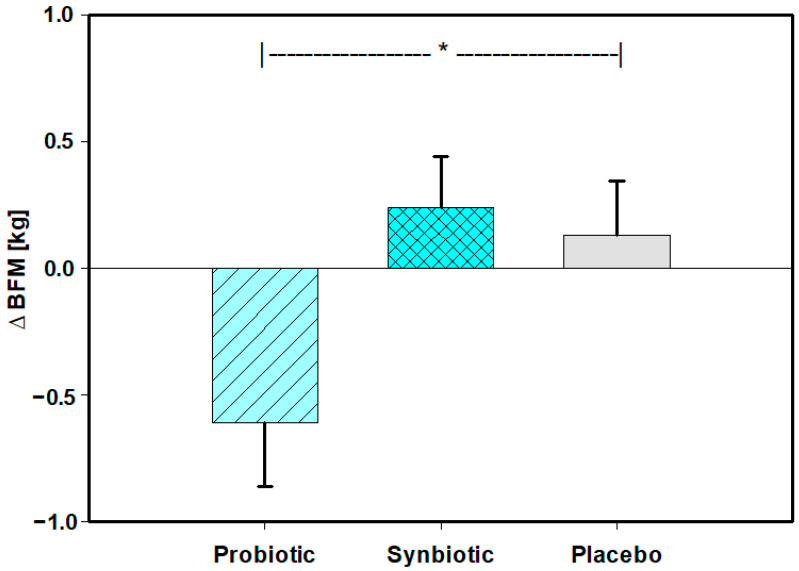
Alteration in body fat mass (∆ BFM) (kg) (primary parameter) during intervention, as assessed with bioimpedance (BIA). Means ± SEMs. * *p* = 0.039 in Holm–Sidak.

The alteration (∆) in BFM (kg) during intervention was significantly (*p* = 0.039) more pronounced in the Probiotic group (−0.61 ± 1.94; mean ± SD) than in the Placebo group (+0.13 ± 1.64; mean ± SD) (Table 4) (Figure 3). ∆ BFM (kg) did not significantly differ between the Synbiotic and Placebo groups (*p* = 0.730). ∆ Rel BFM (%) also did not significantly differ between the Probiotic and Placebo groups (*p* = 0.546) or between the Synbiotic and Placebo groups (*p* = 0.326).

The alteration in visceral adipose tissue (VAT_BIA_) (L) during intervention, as assessed with BIA by additionally taking waist circumference into account, differed significantly (*p* = 0.021) in Kruskal–Wallis ANOVA but only showed a trend towards more pronounced reduction in the Probiotic group compared with the Placebo group (Table 4).

The alteration in fat-free mass (FFM) (kg) during intervention did not significantly differ among the groups (Table 4).

After significance (*p* = 0.015) was found in ANOVA with normally distributed data, post hoc tests (Holm–Sidak) were conducted: *p* = 0.039 Probiotic versus Placebo.

Applying the ANCOVA test to BFM (kg) at visit V3 with covariate BFM (kg) at V1 as an alternative attempt to using the difference ∆(V3-V1) among groups confirmed the significant difference among groups (*p* = 0.015). BFM at V3 (37.99 ± 11.67 kg) adjusted by BFM at V1 (38.60 ± 11.24 kg) in the Probiotic group significantly differed (*p* = 0.046 in Holm–Sidak test) from the that in the Placebo group (39.91 ± 12.14 kg at V3 and 39.78 ± 11.70 kg at V1).

∆ BFM (kg) in the PP population was evaluated using ANOVA due to normally distributed data. The results confirmed those in the FAS population. ∆ BFM (kg) significantly (*p* = 0.012) differed among groups. Holm–Sidak showed a significant (*p* = 0.41) difference between the Probiotic and Placebo groups.

ANCOVA at V3 with baseline (V1) values as the covariate also showed a significant difference among groups (*p* = 0.011), and again, the Probiotic group significantly differed (*p* = 0.044) from the Placebo group in the Holm–Sidak test.

In order to achieve a full ITT population, the missing values of dropout subjects were replaced with the mean values of the corresponding group data, since data were normally distributed. In line with the results in the FAS and PP populations, ANOVA showed a significant (*p* = 0.012) difference among groups, and Holm–Sidak, a significant (*p* = 0.034) difference between the Probiotic and Placebo groups.

ANCOVA adjusted for baseline values was also in line with the results in the FAS and PP populations, showing a significant (*p* = 0.012) difference among groups and a significant (*p* = 0.039) difference between the Probiotic and Placebo groups in the Holm–Sidak test.

#### 3.5.2. Visceral Adiposity Index (VAI) According to Amato (Secondary Parameter)

The ∆ VAI data were not normally distributed due to their leptokurtic kurtosis. Various transformations were tested without achieving a normal distribution. The ∆ VAI did not significantly differ among the groups (*p* = 0.541 in Kruskal–Wallis). The alteration during intervention was −0.201 ± 0.524 in Placebo, −0.154 ± 0.697 in Probiotic and −0.179 ± 0.681 in the Synbiotic group. In line with this, no significant differences among groups were found in the PP population (*p* = 0.523) nor in the ITT population (*p* = 0.442).

#### 3.5.3. Anthropometry and Blood Pressure

The alterations in body weight, BMI, waist circumference and waist-to-height ratio significantly differed between the Probiotic and Placebo groups (Table 5).

#### 3.5.4. Laboratory Parameters

The reduction in fasting plasma glucose levels and HOMA-IR tended to be more pronounced; the difference among groups, however, was not significant (Table 6). The groups did not significantly differ in the alteration in plasma liver enzymes or in CRP. The nominal reduction in liver enzymes indicating liver steatosis (ALT and AST), however, was more pronounced in the Probiotic group than in the Placebo group.

#### 3.5.5. Sonography

In agreement with the bioimpedance data, visceral adipose tissue (VAT_sono_), as assessed with sonography by measuring the sagittal distance between the ventral peritoneum and the aorta, was significantly reduced during intervention in the Probiotic and Synbiotic groups compared with the Placebo group (Table 7). The liver steatosis grade (LSG) also significantly improved during intervention in the Probiotic and Synbiotic groups compared with the Placebo group (Table 7).

Ascites was seen in none of the individuals, which underlines that waist circumference was representative for assessing visceral adipose tissue and not falsified by liquid in the peritoneal cavity.

#### 3.5.6. Medication

Five individuals (*n* = 2 Placebo, *n* = 2 Probiotic and *n* = 1 Synbiotic) were on anti-diabetic medication during the total study period. The anti-diabetic medication (*n* = 4 metformin and *n* = 1 metformin and sitagliptin) did not change during the intervention in these individuals. Only one individual (in the Probiotic group) was on medication dedicated to the reduction in triglyceride levels (fenofibrate). The dosage was not altered during the intervention period. N = 29 individuals in the Probiotic group, *n* = 18 in the Synbiotic group and *n* = 32 in the Placebo group were on anti-hypertensive medication during the intervention period. The alteration in anti-hypertensive medication quantified by the formula X2∗dose_max_ + Y2∗dose_max_+, etc., did not significantly differ (*p* = 0.211 in Kruskal–Wallis test) among groups: Pro 0.0 ± 0.0; Syn 0.03 ± 0.14; Pla 0.03 ± 0.16.

#### 3.5.7. Gastrointestinal Symptoms

The ∆ constipation score of the GSRS [46,47,48] was significantly (*p* = 0.009) improved in the Synbiotic group compared with the Placebo group (Table 8). The alterations in all other components of the GSRS did not differ among groups.

#### 3.5.8. Compliance

Compliance assessed with “pill counting” (difference between dispensed and returned test products) was high and did not significantly (*p* = 0.223) differ among groups: 95.38 ± 7.83% in the Probiotic group, 96.00 ± 9.14% in the Synbiotic group and 96.11 ± 7.34 in the Placebo group.

Likewise, compliance assessed according to Morisky et al., 1986 [51], did not significantly (*p* = 0.382) differ among groups: 0.534 ± 0.569 in the Probiotic group, 0.386 ± 0.491 in the Synbiotic group and 0.483 ± 0.538 in the Placebo group.

#### 3.5.9. Adverse Events (AEs)

The three groups did not significantly differ in occurrence and incidence of adverse events (Table 9).

## 4. Discussion

At baseline, the FAS population was characterized by abdominal overweight (inclusion criterion). The traits related to this feature were in line with this, such as the mean values of BMI, waist circumference, WHtR, blood pressure, BFM, VAT_BIA_, plasma glucose, HbA1c (%), HOMA-IR, QUICKI, liver steatosis grade and VAT_sono_ (SAD). Mean plasma lipid levels and liver enzymes were within the normal ranges. Mean CRP levels were only slightly elevated (normal value < 3 mg/L), indicating low-grade inflammation in only a part of the population.

The three groups did not significantly differ in baseline characteristics and laboratory findings, with the exception of HDL-C and ALT. With 2 out of 24 parameters, this is close to what one might expect as accidental significance. Accidental difference is also indicated by the lack of differences in parameters that are associated with HDL-C as part of metabolic syndrome, such as weight, BMI, waist circumference, WHtR, blood pressure, plasma glucose and HOMA-IR [52,53]. Likewise, parameters associated with ALT, such as body weight, waist circumference, liver steatosis grade, AST and GGT [54], as well as CRP [55], did not differ. Thus, the three groups could be considered comparable in their baseline characteristics.

Out of 180 randomised individuals (ITT population), a high number (*n* = 176) of individuals could be evaluated in the FAS. With *n* = 1 in the Probiotic group, *n* = 2 in the Synbiotic group and *n* = 1 in the Placebo group, the losses were low and comparable among groups. Bias from dropouts during the study conduct, hence, can be considered unlikely.

As indicated by pill counting and the Morisky score [51], the study participants in this double-blind, randomised, placebo-controlled trial had high compliance, and the groups did not differ in compliance. The dropout rate was low, and the results of the primary and secondary parameters were similar in the FAS, PP and ITT populations. This may have been due to rather low and similar incidence of side effects and indicates that the results of the study can be seen as robust.

Also, interference with traits of metabolic syndrome with alteration in anti-diabetic (metformin, sitagliptin), anti-lipemic and anti-hypertensive medication during the intervention period could be excluded.

The reduction in the primary parameter, ∆BFM, of −0.74 kg in the Probiotic group compared with the Placebo group was moderate and within the range (−0.96 kg; 95% CI −1.21 to −0.71) found in the meta-analysis of the studies reporting on this parameter [14]. The result has to be seen against the background that the study participants were not on a diet for weight management. Accordingly, the Placebo group did not lose BFM but even gained some BFM during the study period. Furthermore, the meta-analysis contained randomised controlled trials that were not blinded and may have thus been biased and thereby possibly overestimated the probiotic effect. In obese individuals, who were also included in the present trial, the meta-analysis did not find a significant effect on BFM.

In line with the reduction in BFM by the probiotic, there were better outcomes in visceral adipose tissue, as assessed with BIA; body weight; BMI; waist circumference; WHtR; visceral adipose tissue, as assessed with sonography; and in liver steatosis grade, as assessed with sonography. The reduction in body weight of −1.06 kg compared with the Placebo group was within the range (−0.94 kg; 95% CI −1.17 to −0.70) found in a meta-analysis of the studies reporting on this parameter [14] but higher than that found in the meta-analysis (−0.26 kg; 95% CI −0.75 to +0.23) by Perna et al., 2021 [16]. The reduction in the body mass index of −0.37 kg/m^2^ compared with Placebo was also in the range (−0.55 kg/m^2^; 95% CI −0.86 to −0.23) found in the meta-analysis by Koutnikova et al. [14] and the meta-analysis by Perna et al. [16] (−0.73 kg/m2; 95% CI −1.31 to −0.16), as was the reduction in waist circumference of −1.17 cm compared with −1.31 cm (95% CI −1.79 to −0.83) found in the meta-analyses by Koutnikova et al., 2019 [14], and Perna et al., 2021 [16] (−0.71 cm; 95% CI −1.24 to −0.19). According to the pertinent EFSA guideline [39], the reduction in waist circumference can be seen as a valid measure of visceral adipose tissue, since potentially interfering ascites was excluded in all individuals with the aid of sonography. The VAT_BIA_ data and the SAD assessed with sonography confirmed the reduction in visceral adipose tissue. It is noteworthy that not only the alteration in BFM but also that in a number of other parameters associated with overweight showed significant differences among groups, although sample size estimation made us only expect significant differences in BFM. Together with similar findings in the different populations (FAS, PP and ITT), this indicates the robustness of the findings and may indicate particular efficacy compared with the probiotic category in general.

The somewhat more pronounced reduction in fasting glucose levels and HOMA-IR in the Probiotic group compared with the Placebo group did not attain the significance level (Table 6). Indeed, the sample size that was calculated a priori for these parameters based on data from the meta-analysis by Koutnikova et al., 2019 [14], was considerably higher than the *n* = 56 sample size calculated for BFM. In patients with type 2 diabetes, we estimated a sample size of *n* = 116 for HOMA-IR, *n* = 286 for HbA1c and *n* = 309 for fasting glucose levels. Regarding the meta-analysis effects on glucose metabolism, they were more pronounced in overt type 2 diabetes than in individuals not suffering from diabetes. The number of diabetic individuals in this trial, however, was low, with *n* = 6 in the Probiotic group, *n* = 4 in the Synbiotic group and *n* = 6 in the Placebo group.

The significantly more pronounced reduction in liver steatosis grade, as assessed with sonography, in the Probiotic group compared with the Placebo group is in line with the reduction in liver enzymes indicating liver steatosis (ALT and AST), although it did not attain the level of statistical significance. This may have been due to the fact that most individuals started with baseline levels within the normal range. In patients with NAFLD, liver enzymes were reduced by probiotics according to the meta-analyses by Koutnikova et al., 2019, and Xiao et al., 2019 [14,15].

The mean level of the inflammatory marker CRP was also only slightly above the normal range. This may explain why the nominally more pronounced reduction in the Probiotic group compared with the Placebo group did not attain the level of statistical significance. In fact, low-grade inflammation was suggested to drive hyperphagia by attenuating CCK-induced satiation, and dysregulation of anorexigenic and orexigenic hormones expressed in vagal afferent neurons [56].

The effect on BFM, BMI, body weight and visceral adipose tissue of the probiotic strains used in this study was evidently exerted in spite of a non-significant reduction in CRP. Taking the sample size into account, this does not exclude an effect via this mechanism. However, other mechanisms of reduction in BFM and overweight by probiotics have been suggested, such as induction of butyrate and GLP-1 [57,58] and regulation via chaperone ClpB, a bacterial protein that is a conformational antigen mimetic of α-melanocyte stimulating hormone (α-MSH; a peptide processed from proopiomelanocortin (POMC) implicated in body weight regulation) [59]. Furthermore, microorganisms that are able to metabolize fructose via the mannitol pathway (without producing ethanol) may reduce ethanol production in the gut by competing with microorganisms that metabolize fructose to ethanol and are associated with obesity [21,22,24].

The reduction in constipation, as assessed with the GSRS, in the Synbiotic group compared with the Placebo group may be explained by dietary fibre and the prebiotic effect, respectively. The synbiotic also resulted in better outcomes in visceral adipose tissue and liver steatosis grade, as assessed with sonography, but all other parameters did not significantly improve. The combination of *L. fermentum* strains and prebiotic acacia gum was expected to exert more pronounced effects than the probiotic alone, since acacia gum was shown to increase faecal bifidobacteria and lactobacilli [25,26] and was thus supposed to promote the propagation and effects of *L. fermentum* strains as a synbiotic [27]. Furthermore, prebiotics were shown to increase satiety [28] and reduce body weight, BMI, body fat, postprandial and fasting glucose, HbA1c and insulin levels, and fasting triglycerides and to increase HDL-C and enzymatic makers of liver steatosis according to meta-analyses of RCTs [29,30,31,32,33]. Regarding acacia gum, in particular, effects on traits of metabolic syndrome were also described. Body fat mass (BMI), body fat and the visceral adiposity index were reduced in double-blind, randomised, controlled trials (DB-RCTs) [34,35,36]. In these trials, however, a higher dosage of acacia gum (30 g daily) than that (10 g daily) used in the present trial was administered. Furthermore, the double-blindness of the trials published by Babiker et al. is uncertain, since the placebo dosage was only 5 g, whereas the dosage of acacia gum was 30 g, applied as powder in sachets [34,35,36]. This may have resulted in bias and overestimation of the effect. The lower number of parameters reduced by the synbiotic compared with the probiotic in the present trial may be explained by a lower count of viable lactobacilli in the synbiotic test product compared with the probiotic test product. This was likely due to increased moisture from acacia gum in the synbiotic test preparation. Furthermore, data regarding whether the increase in faecal lactobacilli seen during administration of acacia gum [25,26] is associated with an increase in faecal *L. fermentum* counts are lacking.

## 5. Conclusions

The probiotic resulted in significant improvements in the primary parameter, body fat mass, as assessed with BIA; body weight; BMI; waist circumference; WHtR; visceral adipose tissue, as assessed with sonography; and in liver steatosis grade, as assessed with sonography. The number of significant results exceeded those expected from meta-analyses of previous studies involving probiotics targeting weight management and metabolic health outcomes.

The synbiotic resulted in better outcomes in visceral adipose tissue and liver steatosis grade, as assessed with sonography, and in reduction in constipation. The count of viable lactobacilli was lower in the synbiotic test product than in the probiotic test product, likely due to excessive moisture in test preparation from acacia gum. This may explain the somewhat reduced effect seen in the Synbiotic group compared with the Probiotic group.

## Figures and Tables

**Table 1:** Composition of the test products.

Ingredients Per Sachet	Placebo	Probiotic		Synbiotic	
		CFU a.p.	CFU a.e.s.	CFU a.p.	CFU a.e.s.
*L. fermentum* K7-Lb1		5 × 10^9^	1 × 10^9^	5 × 10^9^	1 × 10^9^
*L. fermentum* K8-Lb1		5 × 10^9^	1 × 10^9^	5 × 10^9^ CFU	1 × 10^9^ CFU
*L. fermentum* K11-Lb3		5 × 10^9^	1 × 10^9^	5 × 10^9^	1 × 10^9^
	mg	mg		mg	
Microcrystalline cellulose	5555.6	5555.6			
Acacia gum				5555.6	
Sucralose	11.3	11.3		11.3	
Cream flavour	97.4	97.4		97.4	
Maltodextrin	185.7	185.7		185.7	
TOTAL	6000.0	6000.0		6000.0	

a.p.: at production; a.e.s.: target colony-forming units (CFU) at the end of shelf-life.

**Table 2:** Baseline characteristics of the FAS population (*n* = 176) at visits V0 and V1, respectively. Waist-to-height ration (WHtR); blood pressure (bp); body fat mass (BFM); fat-free mass (FFM); visceral adipose tissue (VAT); glycated haemoglobin (HbA1c); insulin resistance (HOMA-IR); insulin sensitivity (QUICKI); high-density lipoprotein cholesterol (HDL-C); low-density lipoprotein cholesterol (LDL-C); *C*-reactive protein (CRP); aspartate aminotransferase (AST); alanine transaminase (ALT); gamma-glutamyl transferase (GGT); liver steatosis grade (LSG); visceral adipose tissue, as assessed according to the sagittal diameter (SAD) in sonography.

Time	Parameter	Mean	SD	Median	25%	75%
Point						
V0	Age (years)	60.06	12.33	62.00	53.00	69.00
	Height (cm)	170.1	9.1	170.0	163.3	176.0
V1	Weight (kg)	93.83	17.82	92.30	80.46	102.20
	BMI (kg/m^2^)	32.33	5.45	30.84	28.42	34.87
	Waist (cm)	109.45	12.36	107.5	101.05	115.30
	WHtR	0.644	0.073	0.627	0.590	0.680
	Bp syst (mmHg)	131.2	15.99	130.0	120.0	140.0
	Bp diast (mmHg)	85.00	9.12	85.00	80.00	90.00
	Abs. BFM (kg)	41.25	7.663	42.62	35.47	47.07
	Rel. BFM (%)	38.94	11.72	35.62	31.76	46.04
	FFM (kg)	54.89	11.36	52.55	45.31	64.20
	VAT(BIA) (L)	4.208	2.13	3.595	2.82	5.072
	Glucose (mg/dL)	110.3	17.58	107.0	101.0	117.0
	HbA1c (%)	5.54	0.44	5.50	5.30	5.78
	HOMA-IR	4.282	3.586	3.079	2.275	4.865
	QUICKI	0.321	0.0276	0.323	0.303	0.337
	Cholesterol (mg/dL)	227.8	44.23	225.5	195.3	257.5
	HDL-C (mg/dL)	62.43	14.70	61.5	51.00	70.75
	LDL-C (mg/dL)	146.0	33.38	141.5	124.0	164.75
	Triglycerides (mg/dL)	129.1	56.86	118.0	87.0	162.0
	CRP	0.395	0.742	0.23	0.13	0.46
	AST (U/L)	26.48	9.255	24.0	21.0	29.0
	ALT (U/L)	26.94	15.40	23.5	17.0	31.0
	GGT (U/L)	31.11	23.39	24.0	17.0	34.0
	LSG	1.148	0.637	1.00	0.67	1.67
	SAD	52.21	23.12	50.55	33.4	69.93

**Table 3:** a: Baseline characteristics of the FAS population (*n* = 176) at visits V0 and V1, respectively. Comparison among groups: Probiotic (Pro), Synbiotic (Syn) and Placebo (Pla) groups. *p* according to ANOVA or Kruskal–Wallis ANOVA on ranks depending on the distribution of data. Body mass index (BMI); waist-to-height ration (WHtR); blood pressure (BP); body fat mass (BFM); fat-free mass (FFM); visceral adipose tissue (VAT), as assessed with BIA (VAT_BIA_) and as sagittal diameter of the peritoneal cavity (SAD) as assessed with sonography; liver steatosis grade (LSG). b: Baseline laboratory parameters of the full analysis set (FAS) population (*n* = 176) at visit V1. Comparison among groups: Probiotic (Pro), Synbiotic (Syn) and Placebo (Pla) groups. *p* according to ANOVA or Kruskal–Wallis ANOVA on ranks depending on the distribution of data. Glycated haemoglobin (HbA1c); insulin resistance (HOMA-IR); insulin sensitivity (QUICKI); high-density lipoprotein cholesterol (HDL-C); low-density lipoprotein cholesterol (LDL-C); *C*-reactive protein (CRP); aspartate aminotransferase (AST); alanine transaminase (ALT); gamma-glutamyl transferase (GGT).

(a)										
Time Point	Param.	Group	Size	Mean	SD	Median	25%	75%	*p*	Post Hoc Test If Sign (vs. Control)
V0	Age (years)	Pro	60	61.50	11.30	63.00	56.30	69.80	0.016	
		Syn	60	56.90	14.60	59.00	48.00	68.00		
		Pla	60	61.80	10.30	63.50	54.30	70.00		
	Height (cm)	Pro	60	171.0	9.1	171.0	165.0	176.0	0.190	
		Syn	60	168.0	8.3	169.0	162.0	174.0		Normality
		Pla	60	171.0	9.6	172.0	163.0	178.0		(p ANOVA)
V1	Weight (kg)	Pro	59	95.1	19.1	91.3	82.7	102.2	0.282	
		Syn	58	90.9	17.4	88.3	78.6	101.3		
		Pla	59	95.5	16.9	93.5	83.1	106.2		
	BMI (kg/m^2^)	Pro	59	32.37	5.18	31.07	28.90	34.80	0.612	
		Syn	58	31.98	5.73	30.54	28.25	34.56		
		Pla	59	32.64	5.50	31.37	28.34	36.55		
	Waist (cm)	Pro	59	110.0	12.2	107.2	102.3	114.4	0.291	
		Syn	58	107.6	12.8	105.8	97.5	115.1		
		Pla	59	110.7	12.1	108.3	101.5	119.0		
	WHtR	Pro	59	0.64	0.07	0.63	0.60	0.68	0.547	
		Syn	58	0.64	0.08	0.62	0.59	0.67		
		Pla	59	0.65	0.08	0.64	0.60	0.71		
	BP syst (mmHg)	Pro	59	133.5	16.56	130.0	120.0	140.0	0.259	
		Syn	58	128.6	17.09	130.0	118.8	140.0		Normality
		Pla	59	131.4	14.08	130.0	120.0	140.0		(p ANOVA)
	BP diast (mmHg)	Pro	59	86.4	9.51	90.0	80.0	90.0	0.426	
		Syn	58	83.9	8.43	85.0	80.0	90.0		
		Pla	59	84.7	9.35	85.0	80.0	90.0		
	BFM (kg)	Pro	59	38.60	11.24	35.37	31.75	45.19	0.610	
		Syn	58	38.44	12.34	34.67	31.64	47.00		
		Pla	59	39.78	11.70	38.27	31.38	47.19		
	FFM (kg)	Pro	59	56.47	12.77	54.85	45.26	66.24	0.190	
		Syn	58	52.47	9.63	49.78	44.77	59.76		
		Pla	59	55.68	11.23	53.63	46.29	64.85		
	VAT_BIA_ (L)	Pro	59	4.45	2.27	3.72	3.00	5.30	0.123	
		Syn	58	3.74	1.77	3.36	2.45	4.95		
		Pla	59	4.43	2.27	3.74	2.82	5.10		
	SAD (mm)	Pro	59	53.69	24.64	48.20	33.40	73.80	0.417	
		Syn	58	49.01	22.68	43.60	29.80	66.18		
		Pla	59	53.87	22.03	54.90	37.60	71.70		
	LSG	Pro	59	1.31	0.70	1.33	0.67	1.78	0.120	
		Syn	58	1.08	0.61	0.95	0.56	1.56		
		Pla	59	1.05	0.57	0.89	0.67	1.33		
**(b)**										
**Time Point**	**Param.**	**Group**	**Size**	**Mean**	**SD**	**Median**	**25%**	**75%**	***p***	**Post Hoc Test If Sign (vs. Control)**
V1	Glucose (mg/dL)	Pro	59	112.1	24.4	106.0	101.0	122.0	0.877	
		Syn	58	108.6	13.1	107.0	100.5	116.3		
		Pla	59	110.1	12.7	108.0	100.0	117.0		
	HbA1c (%)	Pro	59	5.58	0.40	5.60	5.30	5.80	0.353	
		Syn	58	5.47	0.32	5.50	5.28	5.60		
		Pla	59	5.55	0.57	5.50	5.20	5.80		
	HOMA-IR	Pro	59	5.11	4.43	3.30	2.31	7.16	0.196	
		Syn	58	4.03	3.76	2.83	2.19	4.45		
		Pla	59	3.71	2.05	3.09	2.29	4.53		
	QUICKI	Pro	59	0.316	0.030	0.320	0.289	0.337	0.164	Normality
		Syn	58	0.325	0.030	0.327	0.307	0.339		(*p*—ANOVA)
		Pla	59	0.321	0.021	0.323	0.306	0.337		
	Cholesterol (mg/dL)	Pro	59	228.17	46.66	219.00	199.00	251.00	0.472	
		Syn	58	232.72	44.82	228.00	198.25	265.00		Normality
		Pla	59	222.68	41.25	226.00	192.00	254.00		(*p*-ANOVA)
	HDL-C (mg/dL)	Pro	59	59.10	14.70	57.00	48.00	68.00	0.006	Pro vs. Pla (*p* = 0.903)
		Syn	58	66.86	13.64	65.00	58.00	76.25		Syn vs. Pla (*p* = 0.041)
		Pla	59	61.41	14.86	60.00	52.00	70.00		(Dunn’s method)
	LDL-C (mg/dL)	Pro	59	148.66	34.65	142.00	128.00	166.00	0.530	
		Syn	58	147.29	35.28	143.00	120.00	166.75		
		Pla	59	142.07	30.23	140.00	120.00	163.00		
	Triglycerides (mg/dL)	Pro	59	128.10	56.49	113.00	87.00	153.00	0.973	
		Syn	58	128.16	51.08	120.00	90.00	167.25		
		Pla	59	130.98	63.19	119.00	87.00	164.00		
	CRP (mg/L)	Pro	59	0.52	1.21	0.23	0.16	0.47	0.136	
		Syn	58	0.36	0.30	0.26	0.13	0.58		
		Pla	59	0.31	0.28	0.21	0.12	0.40		
	AST (U/L)	Pro	59	28.41	9.95	25.00	22.00	33.00	0.138	
		Syn	58	25.83	9.82	24.00	19.00	28.25		
		Pla	59	25.20	7.66	24.00	20.00	28.00		
	ALT (U/L)	Pro	59	30.27	15.26	26.00	21.00	34.00	0.011	Pro vs. Pla (*p* = 0.025)
		Syn	58	25.86	17.45	19.50	15.00	29.50		Syn vs. Pla (*p* = 1.000)
		Pla	59	24.66	12.89	23.00	16.00	28.00		(Dunn’s method)
	GGT (U/L)	Pro	59	32.48	20.96	27.00	20.00	37.00	0.142	
		Syn	58	27.16	16.64	21.00	16.00	31.25		
		Pla	59	33.64	30.24	24.00	17.00	35.00		

**Table 4:** Alteration (∆V3-V1) in body composition during intervention in the FAS, as assessed with body impedance analysis (BIA): absolute (abs) (kg) and relative (rel) (%) body fat mass (BFM); fat-free mass (FFM) (kg); visceral adipose tissue (VAT_BIA_) (L). Comparison among Probiotic (Pro), Synbiotic (Syn) and Placebo groups (Pla). *p* in ANOVA or Kruskal–Wallis ANOVA on ranks depending on the distribution of data; *p* in post hoc tests versus (vs.) Placebo group.

Time Period	Parameter	Group	Size	Mean	SD	Median	25%	75%	*p*	Post Hoc Test If Sign (vs. Control)
∆(V3-V1)	BFM (kg)	Pro	59	−0.61	1.94	−0.46	−1.72	0.81	0.015	Pro vs. Pla (*p* = 0.039)
		Syn	58	0.24	1.52	0.30	−0.79	0.95		Syn vs. Pla (*p* = 0.730)
		Pla	59	0.13	1.64	0.24	−1.30	1.02		Normality (Holm–Sidak)
	BFM (%)	Pro	59	−0.43	1.41	−0.33	−1.31	0.57	0.045	Pro vs. Pla (*p* = 0.546)
		Syn	58	0.15	1.11	0.23	−0.57	1.01		Syn vs. Pla (*p* = 0.326)
		Pla	59	−0.05	1.11	−0.10	−1.03	0.70		(Dunn’s method)
	FFM (kg)	Pro	59	−0.08	1.06	−0.03	−0.64	0.56	0.254	
		Syn	58	0.01	1.19	−0.14	−0.91	0.74		Normality
		Pla	59	0.25	1.12	0.32	−0.47	0.99		
	VAT_BIA_ (L)	Pro	59	−0.20	0.44	−0.19	−0.44	0.04	0.021	Pro vs. Pla (*p* = 0.148)
		Syn	58	−0.02	0.39	−0.03	−0.15	0.15		Syn vs. Pla (*p* = 0.675)
		Pla	59	−0.04	0.36	−0.10	−0.32	0.13		(Dunn’s method)

**Table 5:** Alterations (∆V3-V1) in anthropometric data and blood pressure during intervention. Comparison among Probiotic (Pro), Synbiotic (Syn) and Placebo groups (Pla). *p* in ANOVA or Kruskal–Wallis ANOVA on ranks depending on the distribution of data; *p* in *post hoc* tests versus (vs.) Placebo group. Body mass index (BMI); waist-to-height ration (WHtR); blood pressure (BP).

Time Period	Param.	Group	Size	Mean	SD	Median	25%	75%	*p*	Post Hoc Test If Sign (vs. Control)
∆(V3-V1)	Weight (kg)	Pro	59	−0.69	2.17	−0.60	−1.90	0.75	0.013	Pro vs. Pla (*p* = 0.012)
		Syn	58	0.25	2.02	0.28	−0.84	1.49		Syn vs. Pla (*p* = 1.000)
		Pla	59	0.37	1.87	0.10	−0.60	1.80		(Dunn’s method)
	BMI (kg/m^2^)	Pro	59	−0.24	0.74	−0.20	−0.65	0.27	0.013	Pro vs. Pla (*p* = 0.011)
		Syn	58	0.08	0.70	0.10	−0.32	0.51		Syn vs. Pla (*p* = 1.000)
		Pla	59	0.13	0.63	0.04	−0.22	0.55		(Dunn’s method)
	Waist (cm)	Pro	59	−1.57	2.70	−1.80	−3.00	0.00	0.016	Pro vs. Pla (*p* = 0.033)
		Syn	58	−0.53	2.57	−0.45	−1.83	1.00		Syn vs. Pla (*p* = 1.000)
		Pla	59	−0.40	2.55	−0.50	−2.30	0.70		(Dunn’s method)
	WHtR	Pro	59	−0.009	0.016	−0.011	−0.018	0.000	0.018	Pro vs. Pla (*p* = 0.033)
		Syn	58	−0.003	0.015	−0.003	−0.011	0.006		Syn vs. Pla (*p* = 1.000)
		Pla	59	−0.002	0.015	−0.003	−0.013	0.004		(Dunn’s method)
	BP syst (mmHg)	Pro	58	−1.93	14.64	0.00	−10.00	5.00	0.414	
		Syn	57	−0.18	10.80	0.00	−5.00	5.00		
		Pla	59	−2.88	11.97	0.00	−10.00	5.00		
	BP diast (mmHg)	Pro	58	−3.22	8.05	−5.00	−6.75	0.00	0.049	Pro vs. Pla (*p* = 0.243)
		Syn	57	−0.83	6.52	0.00	−5.00	5.00		Syn vs. Pla (*p* = 0.845)
		Pla	59	−1.51	7.64	0.00	−5.00	0.00		(Dunn’s method)

**Table 6:** Alterations (∆V3-V1) in laboratory parameters during intervention. Comparison among Probiotic (Pro), Synbiotic (Syn) and Placebo groups (Pla). *p* in ANOVA or Kruskal–Wallis ANOVA on ranks depending on the distribution of data; post hoc tests versus (vs.) Placebo group were not performed because of lacking significance in ANOVA. Glycated haemoglobin (HbA1c); insulin resistance (HOMA-IR); insulin sensitivity (QUICKI); high-density lipoprotein cholesterol (HDL-C); low-density lipoprotein cholesterol (LDL-C); *C*-reactive protein (CRP); aspartate aminotransferase (AST); alanine transaminase (ALT); gamma-glutamyl transferase (GGT).

Time Period	Param.	Group	Size	Mean	SD	Median	25%	75%	*p*
∆(V3-V1)	Glucose (mg/dL)	Pro	57	−1.77	10.69	−2.00	−6.50	3.00	0.388
		Syn	57	−1.75	8.08	−2.00	−6.00	3.00	
		Pla	58	−0.03	8.72	−1.00	−5.00	3.00	
	HbA1c (%)	Pro	56	−0.04	0.22	0.00	−0.10	0.10	0.266
		Syn	57	0.02	0.18	0.00	−0.10	0.20	
		Pla	58	0.01	0.24	0.00	−0.10	0.13	
	Insulin (mU/L)	Pro	57	−2.45	7.80	−1.60	−4.05	2.00	0.164
		Syn	57	−0.30	6.41	0.00	−2.40	2.40	
		Pla	58	−0.23	3.78	−0.70	−2.35	1.88	
	HOMA-IR	Pro	57	−0.77	2.88	−0.42	−1.15	0.54	0.164
		Syn	57	−0.16	2.06	−0.11	−0.58	0.59	
		Pla	58	−0.05	1.15	−0.11	−0.68	0.67	
	QUICKI	Pro	57	0.004	0.022	0.008	−0.003	0.014	0.283
		Syn	57	0.000	0.018	0.002	−0.010	0.013	
		Pla	58	0.003	0.015	0.002	−0.007	0.013	
	Cholesterol (mg/dL)	Pro	57	−3.11	24.97	−6.00	−16.00	14.50	0.653
		Syn	57	−6.58	24.53	−4.00	−19.00	6.00	
		Pla	58	−7.16	21.40	−7.00	−18.25	3.50	
	HDL Chol. (mg/dL)	Pro	57	0.28	8.53	1.00	−2.50	4.50	0.468
		Syn	57	−0.77	8.21	0.00	−5.00	4.00	
		Pla	58	0.72	6.51	1.00	−3.00	4.25	
	LDL Chol. (mg/dL)	Pro	57	2.02	20.10	2.00	−8.00	16.00	0.416
		Syn	57	−0.65	19.63	0.00	−9.50	10.50	
		Pla	58	−2.03	17.19	−2.00	−12.25	8.00	
	Triglycerides (mg/dL)	Pro	57	−8.16	47.12	−1.00	−27.50	17.00	0.353
		Syn	57	−15.33	45.66	−10.00	−30.50	6.00	
		Pla	58	−14.29	36.22	−15.00	−34.00	2.25	
	CRP (mg/L)	Pro	57	−0.18	1.24	0.00	−0.11	0.04	0.626
		Syn	57	−0.01	0.28	0.00	−0.09	0.06	
		Pla	58	0.02	0.22	0.00	−0.06	0.05	
	AST (U/L)	Pro	57	−0.12	13.00	0.00	−4.00	2.50	0.137
		Syn	57	−0.60	6.63	−1.00	−4.00	2.00	
		Pla	58	0.74	6.03	0.00	−2.00	3.00	
	ALT (U/L)	Pro	57	−0.54	10.51	0.00	−5.50	2.00	0.260
		Syn	57	1.21	12.39	1.00	−4.00	3.00	
		Pla	58	1.72	9.75	1.00	−2.00	4.00	
	GGT (U/L)	Pro	57	0.97	17.88	−1.00	−3.50	2.00	0.812
		Syn	57	−0.49	5.90	0.00	−2.00	1.50	
		Pla	58	0.85	12.67	−1.00	−2.25	2.00	

**Table 7:** Alterations (∆V3-V1) in visceral adipose tissue (VAT_sono_) during intervention, as assessed according to Armellini [43] using the sagittal diameter (SAD) of the peritoneal cavity, and in liver steatosis grade (LSG), as assessed according to Saverymuttu [44]. Comparison among Probiotic (Pro), Synbiotic (Syn) and Placebo groups (Pla). *p* in ANOVA or Kruskal–Wallis ANOVA on ranks depending on the distribution of data; *p* in post hoc tests versus (vs.) Placebo group.

Time Period	Param.	Group	Size	Mean	SD	Median	25%	75%	*p*	Post Hoc Test If Sign (vs. Control)
∆(V3-V1)	VAT_sono_ SAD (mm)	Pro	57	−6.28	9.34	−5.30	−10.85	−1.20	<0.001	Pro vs. Pla (*p* < 0.001)
		Syn	58	−4.99	11.84	−4.20	−10.55	0.55		Syn vs. Pla (*p* = 0.002)
		Pla	59	2.06	11.65	3.40	−6.10	9.90		(Dunn’s method)
	LSG	Pro	57	−0.25	0.27	−0.22	−0.44	−0.11	<0.001	Pro vs. Pla (*p* < 0.001)
		Syn	58	−0.17	0.24	−0.11	−0.23	0.00		Syn vs. Pla (*p* < 0.001)
		Pla	59	0.05	0.29	0.00	−0.11	0.22		(Dunn’s method)

**Table 8:** Alterations (∆V3-V1) in gastrointestinal symptoms during intervention, as assessed with the Gastrointestinal Score Rating Scale (GSRS) [46,47,48]. Comparison among Probiotic (Pro), Synbiotic (Syn) and Placebo groups (Pla). Number (N); standard deviation (SD). *p* in ANOVA or Kruskal–Wallis ANOVA on ranks depending on the distribution of data; *p* in post hoc tests versus (vs.) Placebo group.

Time Point	Parameter	Group	N	Mean	SD	Median	25%	75%	*p*	Post Hoc Test If Sign (vs. Control)
∆(V3-V1)	Total Score	Pro	58	−0.05	0.48	−0.03	−0.33	0.15	0.356	
		Syn	58	−0.16	0.59	−0.07	−0.27	0.07		
		Pla	58	−0.01	0.43	0.00	−0.15	0.15		
	Pain Score	Pro	58	0.04	0.77	0.00	0.00	0.00	0.119	
		Syn	58	−0.16	0.62	0.00	−0.33	0.00		
		Pla	58	0.05	0.46	0.00	0.00	0.00		
	Reflux Score	Pro	58	−0.10	0.84	0.00	0.00	0.00	0.101	
		Syn	58	−0.24	0.53	0.00	−0.50	0.00		
		Pla	58	−0.05	0.79	0.00	−0.50	0.00		
	Indigestion Score	Pro	58	−0.15	0.88	0.00	−0.56	0.25	0.544	
		Syn	58	−0.02	0.96	0.00	−0.25	0.50		
		Pla	58	−0.04	0.72	0.00	−0.25	0.25		
	Constipation Score	Pro	58	−0.10	0.89	0.00	−0.33	0.00	0.012	Syn vs. Pla (*p* = 0.014) (Dunn’s method)
		Syn	58	−0.36	0.93	0.00	−0.67	0.00		
		Pla	58	0.11	0.69	0.00	0.00	0.42		
	Diarrhoea Score	Pro	58	0.06	0.77	0.00	−0.33	0.33	0.395	
		Syn	58	−0.10	0.87	0.00	−0.33	0.08		
		Pla	58	−0.12	0.76	0.00	−0.33	0.33		

**Table 9:** Adverse events (AEs) during intervention (occurrence = number of individuals who experienced one or more AEs; incidence = number of events). Comparison among Probiotic (Pro), Synbiotic (Syn) and Placebo groups (Pla). *p* in chi-square test for occurrence and in Kruskal–Wallis ANOVA on ranks for incidence. UTI = urinary tract infection; GITI = gastrointestinal tract infection; RTI = respiratory tract infection; laboratory-only findings express which were categorised as clinically significant.

Occurrence	Probiotic	Synbiotic	Placebo	*p*
UTI	0	0	1	0.369 *
GITI	13	8	12	0.484
RTI	4	6	8	0.478
Other infections	4	5	6	0.804
Laboratory	2	0	1	0.367 *
Pain	4	12	8	0.090
Allergy	1	1	0	0.600 *
Other AEs	23	15	16	0.235
Total number of individuals with AEs	34	32	33	0.963
**Incidence**	**Probiotic**	**Synbiotic**	**Placebo**	***p***
UTI	0	0	3	0.371
GITI	15	11	16	0.512
RTI	5	7	9	0.492
Other infections	4	5	6	0.805
Laboratory	2	0	1	0.369
Pain	4	14	11	0.090
Allergy	1	1	0	0.602
Other AEs	33	19	22	0.180
Total number of AEs	64	57	68	0.762

*—chi-square-inaccurate.

## Data Availability

The data presented in this study are available on request from the corresponding author.

## Supplementary Materials

The following supporting information can be downloaded at: https://www.mdpi.com/article/10.3390/nu15133039/s1, Study protocol.
