# Supplementary material for: Effect of a Probiotic and a Synbiotic on Body Fat Mass, Body Weight and Traits of Metabolic Syndrome in Individuals with Abdominal Overweight: A Human, Double-Blind, Randomised, Controlled Clinical Study
Source: Nutrients. 2023 Jul 5;15(13):3039. doi: 10.3390/nu15133039 (PMC10346309; doi:10.3390/nu15133039)
Supplement: Supplementary file 1 [file nutrients-15-03039-s001.zip › nutrients-2470410-supplementary.pdf]

CLINICAL STUDY PROTOCOL

STUDY PRODUCTS: Synbiotic (3 different *L. fermentum* strains plus acacia gum),
Probiotic (same 3 *L. fermentum* strains)
Placebo

STUDY CODE: Slim-LfX2-2021

DATE: 06.05.2021

VERSION: 1.0

STUDY TITLE:

Effect of a synbiotic on body fat mass, weight management, traits of metabolic syndrome and gut permeability in individuals with abdominal overweight:
a randomised, controlled, double-blind clinical study

**Sponsor:**	**Slimbiotics GmbH**<br>**Helmut ESSL, CEO**<br>Tuchlauben 18/12, 1010 Vienna, Austria<br>Phone: +43/1/2350780, Fax: +43/1/2350790<br>Email: essl@hso.at
**Principal Investigator:**	**Dr. Christiane LAUE**<br>**CRC Clinical Research Center Kiel GmbH**<br>Kiel Center of Innovation and Technology<br>Schauenburgerstr. 116, 24118 Kiel, Germany<br>Phone: +49(0)431-5606-870, Fax: +49(0)431-5606-871<br>Email: c.laue@crc-kiel.de
**Scientific adviser:**	**Prof. Dr. Juergen SCHREZENMEIR**<br>**Professor for Internal Medicine**<br>**Johannes-Gutenberg University of Mainz**<br>Langenbeckstr. 1, 55131 Mainz, Germany<br>Phone: +49(0)172 951 9673<br>Email: juergen.schrezenmeir@gmx.de
**Scientific cooperator**	**Prof. Dr. rer. nat. Andre W. FRANKE**<br>Institute of Clinical Molecular Biology<br>UKSH, Campus Kiel<br>Schittenhelmstr. 12<br>24105 Kiel, Germany<br>Phone: +49(0)431 500 - 15 109 or 15 110<br>Email: a.franke@mucosa.de

This study will be conducted according to the protocol and in compliance with the ethical principles stated in the Declaration of Helsinki, the guidelines on Good Clinical Practice (ICH-GCP) and the national regulations (GCP-Verordnung).

List of Abbreviations

AE	Adverse Event
BMI	Body Mass Index
CRP	C-Reactive Protein
CRF	Case Report Form
EPIC	European Prospective Investigation into Cancer and Nutrition
FAS	Full-Analysis-Set
FFQ	Food Frequency Questionnaire
FLI	Fatty Liver Index
FPB	Fasting Plasma Glucose
GCP	Good Clinical Practice
GI	Gastrointestinal
GSRS	Gastrointestinal Symptom Rating Scale
Hb	Hemoglobin
HOMA	Homeostasis Model Assessment
ICF	Informed Consent Form
IEC	Independent Ethics Committee
ID	Identification (number)
ITT	Intention-To-Treat
L.	Lactobacillus
LPSBP	Lipo-PolySaccharid-Binding-Protein
MC	Microcrystalline Cellulose
MSX	Metabolic Syndrome (Syndrom X)
NAFLD	Non-Alcoholic Fatty Liver Disease
PI	Principal Investigator
PP	Per Protocol
SAD	Sagittal Abdominal Diameter
SAE	Serious Adverse Event
SAP	Statistical Analysis Plan
SEM	Standard Error of Mean
SD	Standard Deviation
VAI	Visceral Adiposity Index
WHO	World Health Organisation
WHtR	Waist-to-Height Ratio

TABLE OF CONTENT

1 SYNOPSIS 8

2 SCIENTIFIC BACKGROUND 15

2.1 EFFECT OF INTESTINAL MICROBIOTA AND PROBIOTICS ON HOST METABOLISM 15

2.1.1 Intestinal microbiota 15

2.1.2 Effects of probiotics 16

2.2 EFFECTS OF PREBIOTICS 17

2.3 THE PERTINENT LACTOBACILLI 18

2.3.1 Origin 18

2.3.2 Anti-inflammatory effects in vitro 19

2.3.3 Defensin-inducing properties in vitro 19

2.3.4 Rational of selection of the pertinent strains 19

2.3.5 Rational of dosage of the pertinent strains 19

2.3.6 Safety 19

2.4 ACACIA GUM 20

2.4.1 Origin, chemistry and usage 20

2.4.2 Prebiotic effect (bifidogenicity, lactobacilli) 20

2.4.3 Effects on weight management 20

2.4.4 Effects on glucose metabolism 21

2.4.5 Effects on plasma lipids 22

2.4.6 Safety 22

3 AIM 22

3.1 STUDY OBJECTIVES AND PARAMETER 23

3.1.1 Primary target parameter 23

3.1.2 Secondary target parameter 23

3.1.3 Exploratory parameters 23

4 STUDY DESIGN AND POPULATION 25

4.1 SUMMARY OF STUDY DESIGN 25

4.2 DESCRIPTION OF THE STUDY DESIGN AND CONDUCT 25

4.2.1 Scheduled site visits 26

4.3 SUBJECT SELECTION AND WITHDRAWAL 26

4.3.1 Inclusion criteria 26

4.3.2 Exclusion criteria 27

4.3.3 Withdrawal or elimination criteria 27

4.4 PREMATURE DISCONTINUATION OF THE STUDY 27

4.4.1 Subject‘s withdrawal 27

4.4.2 Subject’s discontinuation 28

4.4.3 Stop of a part or all of the study 28

4.5 REPLACEMENT CONDITIONS 28

5 STUDY CONDUCT ... 28

5.1 SCHEDULE OF ASSESSMENT ... 28

6 STUDY VISITS ... 29

6.1 SCREENING VISIT (V0) ... 29

6.2 RANDOMIZATION AND INTERVENTIONAL VISIT 1 (V1) ... 30

6.3 VISIT 2 (V2): ... 31

6.4 VISIT 3 (V3) ... 31

7 TEST PRODUCTS ... 32

7.1 SYNBIOTIC PRODUCT COMPONENTS ... 32

7.1.1 Lactobacillus fermentum strains ... 32

7.1.2 Acacia gum ... 33

7.1.3 Maltodextrin ... 34

7.1.4 Sucralose ... 34

7.1.5 Cream flavour ... 34

7.1.6 Composition of the synbiotic product (summary table) ... 35

7.2 PROBIOTIC PRODUCT COMPONENTS ... 35

7.2.1 Lactobacillus fermentum strains ... 35

7.2.2 Composition of the probiotic product (summary table) ... 35

7.3 PLACEBO PRODUCT ... 36

7.3.1 Microcrystalline Cellulose ... 36

7.3.2 Further components ... 36

7.3.3 Composition of the placebo product (summary table) ... 36

7.4 PRODUCTION OF TEST PRODUCTS ... 37

7.5 MODE OF CONSUMPTION ... 37

7.6 STORING, HAND OUT AND RETURN BACK OF TEST PRODUCTS ... 38

7.7 ACCOUNTABILITY OF TEST PRODUCTS ... 38

8 SAFETY ASPECTS ... 38

8.1 TEST PRODUCTS ... 38

8.1.1 Verum products ... 38

8.1.2 Placebo product ... 38

8.2 BIOELECTRICAL IMPEDANCE ANALYSES (BIA) ... 38

8.3 ADVERSE EVENTS (AE'S) ... 39

8.3.1 Relationship of adverse event to the test product ... 40

8.3.2 Reporting/Notification of adverse events ... 40

8.3.3 Monitoring of persons following the occurrence of adverse events ... 41

8.4 BLOOD PRESSURE ... 41

8.5 MONITORING OF LABORATORY PARAMETERS ... 41

8.6 KEEPING BACK UP BLOOD MATERIAL ... 41

9 STATISTICAL METHODS ... 42

9.1 DETERMINATION OF SAMPLE SIZE ... 42
9.2 DEFINITION OF SETS TO BE ANALYZED ... 42
9.2.1 Intention-To-Treat (ITT) collective ... 42
9.2.2 Full Analysis (FAS) Set ... 42
9.2.3 Per-Protocol (PP) Set ... 42
9.3 STATISTICAL TESTS ... 43
10 ETHICAL CONSIDERATIONS ... 43
10.1 ETHICS COMMITTEE ... 43
10.2 CHANGES TO THE STUDY PROTOCOL ... 44
10.3 PROTOCOL DEVIATIONS ... 44
10.4 INFORMED CONSENT FORM ... 44
11 DATA MANAGEMENT AND MONITORING ... 44
11.1 IDENTIFICATION OF SOURCE DOCUMENTATION AND DATA ... 44
11.1.1 Source Document Definition ... 44
11.1.2 Source Data Definition ... 45
11.1.3 Management of source documents during the study ... 45
11.2 DIRECT ACCESS TO SOURCE DATA/DOCUMENTS ... 45
11.3 LAYOUT OF THE CASE REPORT FORM (CRF) ... 46
11.4 IDENTIFICATION OF SUBJECTS ... 46
11.5 SUBJECT DIARY AND QUESTIONNAIRES ... 46
11.6 ELECTRONIC DATA CAPTURE ... 46
11.7 DATABASE MANAGEMENT ... 46
12 ARCHIVAL ... 46
12.1 GENERAL ... 46
12.2 INVESTIGATOR ... 47
13 CONFIDENTIALITY ... 47
14 INSURANCE ... 47
15 OWNERSHIP OF RESULTS ... 47
16 STUDY REPORT AND PUBLICATION ... 48
17 RESPONSIBILITIES ... 48
17.1 SPONSOR ... 48
17.2 INVESTIGATOR ... 48
18 REFERENCES ... 49
19 APPENDICES ... 54
APPENDIX I: SUCRALOSE ... 54
APPENDIX II: AROMA PANNA DRY ... 56
APPENDIX III: MALTODEXTRIN ... 57
APPENDIX IV: DECLARATION OF HELSINKI ... 59
APPENDIX V: SERIOUS ADVERSE EVENT FORM ... 67

20 SIGNATURES 70

1 SYNOPSIS

**Study title**	Effect of a synbiotic on body fat mass, weight management, traits of metabolic syndrome and gut permeability in individuals with abdominal overweight a randomised, controlled, double-blind clinical study
**Study code**	Slim-LfX2-2021
**Sponsor**	Slimbiotics GmbH, Vienna / AUSTRIA
**Study centre/ country**	Clinical Research Center, Kiel / GERMANY
**Planned study period**	June 2021 – December 2022
**Study objectives**	To assess the effect of a synbiotic on body fat mass, body weight, long-term glycemia, insulin resistance and gut permeabilty in patients with abdominal overweight
**Study design**	Randomised, placebo-controlled, double-blind study with three parallel arms

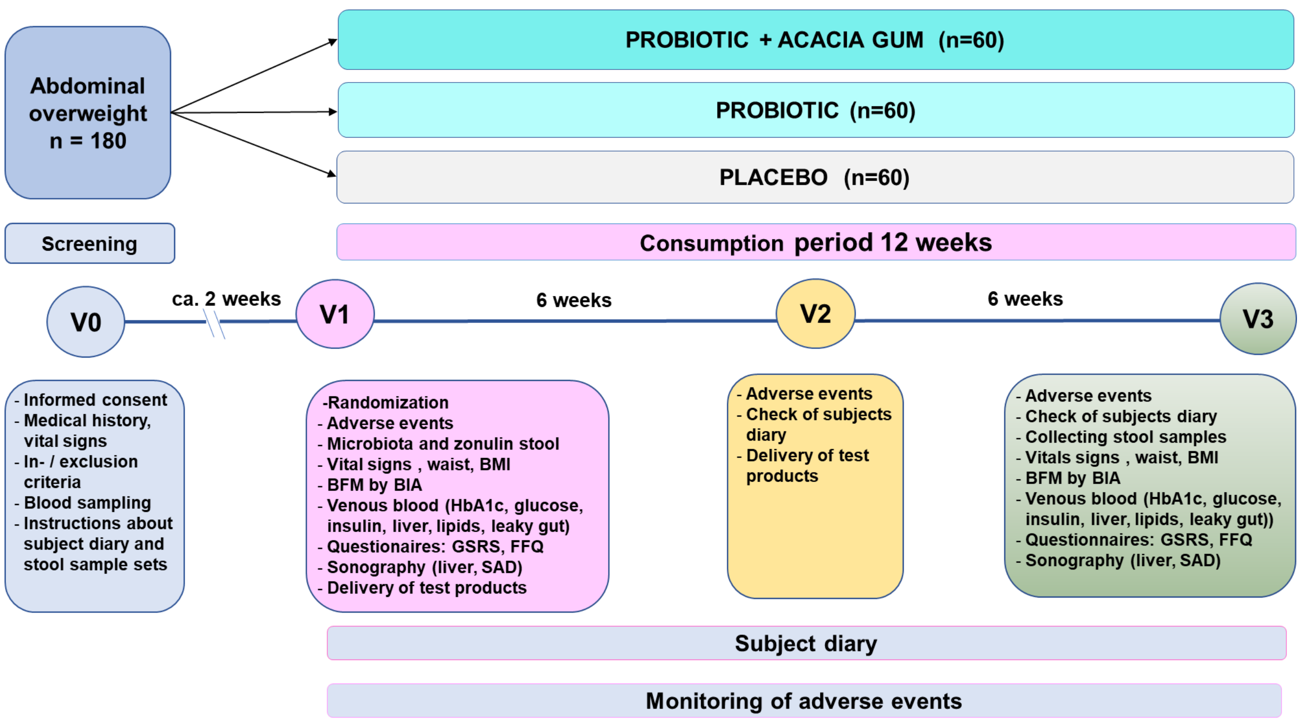

**Planned number of subjects**	n=180, 3 groups with n = 60, each

**Inclusion criteria**	1. Overweight or obese (BMI ≥ 25)<br>2. Elevated waist circumference (>94cm and >80cm (for European men and women, respectively)<br>3. Age ≥ 18<br>4. Written informed consent
**Exclusion criteria**	Any of the following is regarded as a **criterion for exclusion from enrollment** in the study:<br>1. Subjects currently enrolled in another clinical study<br>2. Subjects having finished another clinical study within the last 4 weeks before inclusion<br>3. Hypersensitivity, allergy or intolerance against any compound of the test products (e. g. acacia gum)<br>4. Condition after implantation of a cardiac pacemaker or other active implants<br>5. Sulfonylurea treatment<br>6. Any disease or condition which might compromise significantly the hepatic (ascites), hematopoietic, renal, endocrine, pulmonary, central nervous, cardiovascular, immunological, dermatological, gastrointestinal or any other body system with the exception of the conditions defined by the inclusion criteria<br>7. History of or present liver deficiency as defined by Quick < 70%<br>8. Regular medical treatment including OTC, which may have impact on the study aims (e. g. probiotics containing supplements, laxatives, steroids etc.)<br>9. History of hepatitis B, C, HIV<br>10. Major cognitive or psychiatric disorders<br>11. Subjects who are scheduled to undergo any diagnostic intervention or hospitalization which may cause protocol deviations<br>12. Simultaneous study participation by members of the same household<br>13. Pregnancy and lactation<br>14. Ascites as assessed by sonography<br>15. Any diet to lose body weight<br>16. Eating disorders or vegan diet<br>17. Anorexic drugs<br>18. Present drug abuse or alcoholism<br>19. Legal incapacity

**Investigational products: Mode of administration/ Dose schedule**	Group 1: **placebo:** 5.6g microcrystalline cellulose. Further components are maltodextrin (185.7mg), sucralose (11.3mg) and flavour (97.4mg)<br><br>Group 2: **verum:** a **symbiotic (primary/secondary parameters)** consisting of the strains *Lactobacillus fermentum* K7-Lb1 (≥1x10^{9}CFU), *L. fermentum* K8-Lb1 (≥1x10^{9}CFU), *L. fermentum* K11-Lb3 (≥1x10^{9}CFU) + 5.6g acacia gum (acacia gum). To ensure identical characteristics with placebo, sucralose, cream flavour and maltodextrin are added too.<br><br>Group 3: **probiotic (exploratory parameters)**: consisting of the strains *Lactobacillus fermentum* K7-Lb1 (≥1x10^{9}CFU), *L. fermentum* K8-Lb1 (≥1x10^{9}CFU), *L. fermentum* K11-Lb3 (≥1x10^{9}CFU). To ensure identical characteristics with placebo, 5.6g microcrystalline cellulose, sucralose, cream flavour and maltodextrin are added too.<br><br>In total each of the three products contain 6g powder per sachet.<br><br>The test products should be consumed two times daily in the morning and in the evening well dispersed in a cold, non-alcoholic drink (200ml), preferably in drinking water.
**Intervention period per subject**	12 weeks: 84(+6) days
**Primary parameter**<br>**alteration V3-V1**<br>**symbiotic vs placebo group**	Body Fat Mass (BFM) as assessed by bioelectrical impedance analysis (BIA)
**Secondary parameters**<br>**alteration V3-V1**<br>**symbiotic vs placebo group**	Visceral Adiposity Index (VAI) after *Amato et al. Diabetes Care 33:920–922*, 2010: (Females: VAI = WC / (36.58 + (1.89 × BMI)) × (TG/0.81) × (1.52/HDL)), (Males: VAI = (WC / (39.68 + (1.88 × BMI)) × (TG/1.03) × (1.31/HDL)

**Exploratory parameters**<br>alteration V3-V1<br>**symbiotic vs placebo group**<br>**and**<br>**probiotic vs placebo group**	Waist, Waist-to-Height Ratio (WHtR), body weight, BMI<br>MSX-index (alteration V3-V1) according to ATP III/IDF* definitions (Waist x FPG x Tg x 1/HDL-C x BP_{sys} x BP_{dias}) (ascites as interfering cause for alteration of these measures will be excluded by abdominal sonography)<br>**National Cholesterol Education Program Adult Treatment Panel (NCEP ATP III) and International Diabetes Federation (IDF)*<br>HbA1c<br>HOMA-IR (Homeostasis Model Assessment (HOMA)-IR = glucose [mmol/L] x insulin [µU/ml]/22,5)<br>Blood pressure and pulse<br>Blood parameters: FPG, insulin, Tg, HDL-C, LDL-C, hsCRP, AST, ALT, yGT, cytokeratin-18 fragment (markers of liver steatosis/steatohepatitis), gut permeability parameters: zonulin, LPS<br>Fatty Liver Index (FLI) calculated from serum triglyceride, body mass index, waist circumference, and gamma-glutamyltransferase according to *Bedogni et al. BMC Gastroenterol 2006; 6: 33*: FLI = (e 0.953*loge (triglycerides) + 0.139*BMI + 0.718*loge (ggt) + 0.053*waist circumference - 15.745) / (1 + e 0.953*loge (triglycerides) + 0.139*BMI + 0.718*loge (ggt) + 0.053*waist circumference - 15.745) * 100<br>NAFLD liver fat score according to *Kotronen et al. Gastroenterology 2009; 137: 865-872:* NAFLD liver fat score -2.89 + 1.18*metabolic syndrome (yes = 1/no= 0) + 0.45*type 2 diabetes (yes= 2/no= 0) + 0.15*fS-insulin (mU/L) + 0.04*fS-AST (U/L) - 0.94*AST/ALT<br>Liver steatosis grade: Sonographical diagnosis of liver steatosis and quantification follows the criteria given by *Saverymuttu et al. (1986)* resulting in a grading from 0 = no steatosis, 1 = slight steatosis, 2 = moderate steatosis and 3 = severe steatosis.<br>Sagittal abdominal diameter (SAD): Distance between the under surface of the rectus muscle and the anterior wall of the aorta – measure for visceral fat after *Armellini et al*. 1991: *Sagittal abdominal diameter as a practical predictor of visceral fat,* Int J Obes. *1991*<br>Bio-Impedance-Analysis (BIA) for assessment of body fat mass (BFM), visceral fat mass and other parameters of body composition.<br>anti-diabetic medication (X_1*$dose_{max}$,); anti-hypertensive medication (X_2*$dose_{max}$ + Y_2*$dose_{max}$ + etc.); anti-lipidemic medication (X_3*$dose_{max}$ + Y_3*$dose_{max}$ + etc.) (X_i<1, Y_i<1, maximal daily dose for each drug is assumed to be 1)<br>Gastrointestinal Symptom Rating Scale (GSRS according to *Svedlund 1988, Dimenäs 1995, Revicki 1998*), related to the last 7 days before V1 and V3<br>Compliance (counting consumed test products; *Morisky* score)<br>fecal bifidobacterial and lactobacilli at V1 and V3<br>Primary and secondary parameters at V1 and V3
**Inclusion/eligibility parameters (V0)**	BMI, glucose, waist circumference (WC), systolic and diastolic BP, heart rate, Na^+, K^+, creatinine, γ-GT, AST, ALT, AP, CHE, CRP, leukocytes, erythrocytes, Hb, hematocrit, MCV, MCH, MCHC, thrombocytes, LDL-C, HDL-C, triglycerides, Quick (prothrombin time)

**Schedule of visits and assessments**	**Visit 0**<br>Screening visit, checking subject's eligibility<br>Formalities in line with ICH-GCP: subject information & ICF, medical history, concomitant medication, physical examination, vital signs, body height, body weight, waist circumference, waist-to-height-Ratio (WHtR), BMI, check of inclusion & exclusion criteria, blood sampling for eligibility parameters and for exclusion parameters (s. section above).<br>Scheduling the study visits, hand over the subject diary and instructions how to keep it.<br>Instruction to return to next visits in fasting condition and to bring back the subject diary and the stool sample.<br>Delivery of two stool sample kits to be used at the first bowel movement in the space of the last two days before V1 resp. V3 (for determination of fecal bifidobacterial, lactobacilli and Zonulin).
	**Visit 1 ca. 2 weeks after V0**<br>Starting intervention (during the visit)<br>Checking the subject diary and possible adverse events between V0 and V1; randomisation, instructions to bring back the subject diary and unused study products to the next visit.<br>Vital signs, body weight, waist circumference, waist-to-height-ratio (WHtR), BMI<br>Body fat mass (BFM), lean body mass (LBM) and other parameters by bioelectrical impedance (BIA) using the medical Body Composition Analyzer seca mBCA 515.<br>Fasting blood test (HbA1c, glucose, insulin, triglyceride, HDL-C, LDL-C, CRP, ALT, AST, γ-GT, CHE, LPS, cytokeratin-18 fragment)<br>Collecting stool samples (microbiota, zonulin)<br>Questionnaire FFQ (EPIC-Potsdam Food Frequency Questionnaire) to recall dietary intake during the last 12 months<br>Questionnaire to evaluate gastrointestinal symptoms (GSRS: Gastrointestinal Score Rating System according to *Svedlund 1988, Dimenäs 1995, Revicki 1998*) related to the last 7 days)<br>Sonography (liver steatosis grade after *Saverymuttu et al. 1986* and after *Webb et al. 2008*; exclusion of ascites; distance between the under surface of the rectus muscle and the anterior wall of the aorta – measure for visceral fat after *Armellini et al. 1991*<br>Completing eCRFs, accountability log

	**Visit 2**<br><br>**6 weeks ±3 days after V1**<br><br>Checking the subject diary and possible adverse events between V1 and V2; compliance assessment (counting of products given back)<br><br>Delivery of test products for intervention period V2-V3<br><br>Completing eCRFs, accountability log
	**Visit 3**<br><br>**12 weeks +6 days after V1**<br><br>Checking the subject diary and possible adverse events between V2 and V3; compliance assessment (counting of products given back, score according *Morisky et al. 1986*)<br><br>Information from the investigator about subject's medical findings<br><br>Vital signs, body weight, waist circumference, waist-to-height-ratio (WHtR), BMI<br><br>Body fat mass (BFM), lean body mass (LBM) and other parameters by bioelectrical impedance (BIA)<br><br>Fasting blood test (HbA1c, glucose, insulin, triglyceride, HDL-C, LDL-C, CRP, ALT, AST, γ-GT, CHE, LPS, cytokeratin-18 fragment)<br><br>Collecting stool samples (microbiota, zonulin)<br><br>Questionnaire FFQ to recall dietary intake during the last 12 weeks<br><br>Questionnaire GSRS with regard of the last 7 days<br><br>Sonography (liver steatosis grade after *Saverymuttu et al. 1986* and after *Webb et al. 2008;* exclusion of ascites; peritoneum-aorta distance – measure for visceral fat after *Armellini et al*. 1991)<br><br>Collecting all study related document and source data (diary, questionnaires etc.)<br><br>Completing eCRFs, accountability log
**Optional visits**	According to investigators opinion

**Statistical analyses**	The statistical methodology will be described in more detail in the statistical analysis plan finalized before database lock and unblinding.<br><br>For the primary goal, the comparison of BFM between Placebo versus symbiotic group Mann Whitney U test is applied. This is also applied for the secondary goals.<br><br>Comparison within the groups will be performed for each parameter using two-sided statistical tests (parametric such as paired *t* test and/or non-parametric such as Wilcoxon Signed Rank Test according to the distribution).<br><br>Descriptive statistics will be given in mean, SD, 95% CI of mean, median, 25th and 75th percentiles, frequency of observation. In figures mean ± SEM will be used for depicting the results.<br><br>The statistical evaluation will be performed at a 5% significance level acc. to the ITT principle in the FAS as main target population. Evaluation in the ITT and PP population will serve for sensitivity analyses with regard to the primary and secondary parameters.<br><br>For the secondary parameters adjustment for multiple testing will be done according Bonferroni-Holm.

2 SCIENTIFIC BACKGROUND

2.1 Effect of intestinal microbiota and probiotics on host metabolism

2.1.1 Intestinal microbiota

In recent years numerous studies provided evidence that the intestinal microbiota has a key role in the interface between dietary factors and host biology and that overweight, diabetes and liver steatosis, known to depend on dietary factors, are associated with alterations in composition and diversity of intestinal microbiota.

In N=123 non-obese and N=169 obese Danish individuals with a low bacterial richness (23% of the population) was characterized by more marked overall adiposity, insulin resistance and dyslipidaemia and inflammatory phenotype (Le Chatelier et al., 2013). In N=345 Chinese with T2D and controls patients with type 2 diabetes was characterized by a decrease in the abundance of some butyrate-producing bacteria and an enrichment of other microbial functions conferring sulphate reduction (Qin et al, 2012).

The composition of the microbiota was associated with certain dietary patterns and could be affected by dietary and therapeutic interventions. Fecal microbiota were strongly associated with long-term diets (N=98): Bacteroides with intake of protein and animal fat, Prevotella with carbohydrate intake. High-fat/low-fiber versus low-fat/high-fiber diet changed microbiome composition (N=10) (Wu et al., 2011).

In N=98 elderly microbiota composition correlated with frailty, co-morbidity, nutritional status, markers of inflammation (Claesson et al., 2012).

In obese or overweight (N=49) individuals with reduced microbial gene richness (40%) metabolic disorders and low-grade inflammation were more pronounced. However, dietary intervention (weight management) improved low gene richness and clinical phenotypes, but was less efficient for inflammation variables in individuals with lower gene richness (*Cotillard et al,* 2013). An exclusively animal or plant diet altered the microbiota composition and overwhelmed interindividual differences. The animal diet resulted in an increase in bile salt tolerant microorganisms (*Alistipes, Bilophila*, and *Bacteroides*) and a decrease in firmicutes, which ferment plant polysaccharides (*Roseburia, Eubacterium rectale*, Ruminococcus bromii) (*David et al,* 2014). The increase in *Bilophila wadworthia* under animal diet support the link between dietary fat, bile salt metabolism and growth of microorganisms, which favour inflammation (*Devkota, 2012*). In the Belgian Flemish Gut Flora Project (FGFP; discovery cohort; N=1106) und Dutch LifeLines-Deep study; N=1135) *Akkermansia* abundance was negatively associated with BMI and triglycerides. One bicluster was identified which comprised 15 genera, incl. some Clostridia and hydrogenotrophic genera, e.g. *Methanobrevibacter* and *Desulfovibrio*. In this cluster women and individuals with lower body weight dominated. Another bicluster comprised 7 genera, incl. *Bacteroides* und *Parabacteroides*. In this cluster individuals with lower diversity of microbiome dominated as well as preference of low dietary fibre white bread (*Falony et al*., 2016; *Everard et al*., 2013; *Flandroy* et al., 2018).

2.1.2 Effects of probiotics

Probiotics were defined as "A preparation of or a product containing viable, defined microorganisms in sufficient numbers, which alter the microflora (by implantation or colonization) in a compartment of the host and by that exert beneficial health effects in this host" (*Schrezenmeir & de Vrese*., 2001).

The beneficial effect of the oral administration of probiotics on in overweight and obesity, respectively, has been demonstrated by meta-analyses of controlled clinical trials.

In a meta-analysis of 25 RCTs with N = 1931 *body weight* was significantly reduced compared to control (MD -0.59 kg; 95% CI - 0.30; -0.87; P < 0.05), even though the effect was rather small (*Zhang Q* et al., 2016). Accordingly, *BMI* (kg/m^2) was reduced (MD -0.49; 95% CI -0.24; -0.74; P < 0.05) whereby the reduction was more pronounced when baseline BMI was ≥ 25 kg/m^2 (*Zhang Q* et al., 2016). *Waist circumference* was not significantly reduced in a meta-analysis of DB-RCTs with N = 304 individuals (MD -2.11 cm; 95% CI −3.543; -0.677; P < 0.004; I^2 = 0%, P = 0.63) (Sun J, Buys N. Ann Med 2015;47:430-40).

The effect of probiotics on glucose metabolism in N = 1105 individuals with the metabolic syndrome was demonstrated in a meta-analysis of 17 RCTs. Fasting *blood glucose* (FBG) was slightly reduced (MD -0.31 (mmol/L); 95% CI -0.56, -0.06; p = 0.02; I^2 = 92%, P < 0.001) (*Ruan Y* et al., 2015). Insulin levels and *HOMA-IR* (as measure of insulin resistance) was also reduced; HOMA-IR on the basis of 8 RCTs comprising N = 635 (MD - 0.48; 95% CI -0.83, -0.13; p = 0.007; I^2 = 93%, p < 0.01) (*Ruan Y* et al., 2015). In a meta-analysis of 19 RCTs (N = 1002) FBG (mmol/L) was again reduced (MD −0.18; 95% CI −0.37, 0.00; p = 0.05; I^2 = 72%, P < 0.001) (*Nikbakht* et al., 2016). In both meta-analyses the effects were more pronounced in case of: Hyperglycemia, antidiabetics therapy, duration of intervention > 8 weeks, the use of multi-species preparations and high dosage of microbes (> 10^{11} CFU/day).

In a meta-analysis of 8 RCTs comprising N = 429 type 2 diabetes patients *FBG* was reduced (MD -15.92 mg/dL; 95% CI -29.75; -2.09; P = 0.02; I^2 = 64% P < 0.05) (Zhang Q et al, Wu Y, Fei X. Medicina 2016;52:28-34). Accordingly, *HbA1c* was reduced (MD -0.54 %; 95% CI -0.82; -0.25; P < 0.001; I^2 = 4%; P = 0.35). Again *HOMA-IR* (MD -1.08; 95% CI -1.88; -0.28 P < 0.01; I^2 = 0%; P = 0.68) and insulin were reduced.

Similar results were seen in a meta-analysis of 12 RCTs comprising N = 770 T2D patients (*Hu YM* et al., 2017). In addition, a reduction of serum triglycerides and cholesterol and an increase in HDL-C were reported (*Hu YM* et al., 2017).

Obesity and particularly T2D and liver steatosis are associated with low grade *inflammation* of the liver, adipose and muscle tissue and this inflammation of the key tissues of glucose metabolism is regarded as driving force for insulin resistance (*Elshaghabee* et al., 2019). The origin of this inflammation is considered to be the result of an impaired barrier function of the intestine permitting the translocation of Gram-negative bacteria and its lipopolysaccharides (LPS), the endotoxins. The barrier function, again, is modulated by bacteria. Probiotics have been demonstrated in vitro and in vivo to improve the intestinal barrier function (*Elshaghabee* et al., 2019).

For this reason, the effect of probiotics on inflammation markers gained interest. In a meta-analysis of 2 RCTs with N=69 *CRP* was reduced (MD -1.207; 95% CI −2.425; -0.011; P < 0.052; I^2 = 0%, P = .002) (*Sun J* et al., 2015).

In a meta-analysis of 4 RCTs with N = 134 in individuals with liver steatosis (NAFLD or NASH) probiotics significantly reduced *ALT, AST*, HOMA-IR and *TNFα* (Ma et al., 2013).

In a very recent meta-analysis comprising N=58 studies and N=3701 individuals the effect of probiotics was evaluated in the total population and sub-populations with normal weight, overweight, obese, T2D, gestational diabetes and liver steatosis (Koutnikova et al., 2019). In overweight but not obese subjects, probiotics induced improvements in: body weight (k = 25 trials, d = −0.94 kg mean difference, 95% CI −1.17 to −0.70, I^2 = 0.0%), body mass index (k = 32, d = −0.55 kg/m², 95% CI −0.86 to −0.23, I^2 = 91.9%), waist circumference (k = 13, d = −1.31 cm, 95% CI −1.79 to −0.83, I^2 = 14.5%), *body fat mass* (k = 11, d = −0.96 kg, 95% CI −1.21 to −0.71, I^2 = 0.0%) and *visceral adipose tissue mass* (k = 5, d = −6.30 cm², 95% CI −9.05 to −3.56, I^2 = 0.0%). In type 2 diabetics, probiotics reduced fasting glucose (k = 19, d = −0.66 mmol/L, 95% CI −1.00 to −0.31, I^2 = 27.7%), glycated haemoglobin (k = 13, d = −0.28 pp, 95% CI −0.46 to −0.11, I^2 = 54.1%), insulin (k = 13, d = −1.66 mU/L, 95% CI −2.70 to −0.61, I^2 = 37.8%) and homeostatic model of insulin resistance (k = 10, d = −1.05 pp, 95% CI −1.48 to −0.61, I^2 = 18.2%). In subjects with fatty liver diseases, probiotics reduced alanine (k = 12, d = −10.2 U/L, 95% CI −14.3 to −6.0, I^2 = 93.50%) and aspartate aminotransferases (k = 10, d = −9.9 U/L, 95% CI −14.1 to -5.8, I^2 = 96.1%).
CRP was reduced (k = 41, d = −0.48 mg/L; 95% CI −0.76 to −0.21; I^2 = 67.1%) in the total population.

This meta-analysis also showed more pronounced effects for certain species and strains, respectively. Effects of certain strains can be expected to be stronger due to strain and species-specific properties, e.g., gastrointestinal transit, anti-inflammatory properties, defensin induction, barrier function, existing mannose-pathway, bile salt metabolism, energy utilisation from indigestible polysaccharides, etc. (Njeru et al., 2010; Ghadimi et al., 2008; Ghadimi et al., 2011; Ghadimi et al., 2014; Elshaghabee et al., 2016).

2.2 Effects of prebiotics

Prebiotics were defined as “a non-digestible food ingredient that beneficially affects the host by selectively stimulating the growth and/or activity of one or a limited number of bacteria in the colon” (Gibson & Roberfroid, 1995).
The term “synbiotic” is used when a product contains both probiotics and prebiotics. Because the word alludes to synergism, this term should be reserved for products in which the prebiotic compound selectively favors the probiotic compound (Schrezenmeir & de Vrese, 2001).
In a meta-analysis of 13 RCTs comprising N=513 individuals with overweight (BMI ≥25 kg/m²) prebiotics reduced plasma triglycerides (MD = −0.72 mmol/L; 95% CI −1.20, −0.23), cholesterol (MD = −0.25; 95% CI −0.48, −0.02), LDL-C (MD = −0.22; 95% CI −0.44, −0.00) and increased HDL-C (MD = +0.49; 95% CI 0.01, 0.97). Synbiotics reduced triglycerides and fasting insulin (Beserra et al., 2015).
In a meta-analysis of 26 RCTs (N=831) prebiotics reduced postprandial satiety (MD = −0.57, 95 % CI −1.13, −0.01), glucose (MD = −0.76, 95 % CI −1.41, −0.12) and insulin levels (MD = −0.77, 95 % CI −1.50, −0.04) (Kellow et al., 2014).
In a meta-analysis of 12 RCTs (N=609) soluble fiber supplementation reduced BMI by 0.84 (95% CI: 21.35, 20.32; P = 0.001), body weight by 2.52 kg (95% CI: 24.25, 20.79 kg; P = 0.004), body fat by 0.41% (95% CI: 20.58%, 20.24%; P, 0.001), fasting glucose by 0.17 mmol/L (95% CI: 20.28, 20.06 mmol/L; P = 0.002), and fasting insulin by 15.88 pmol/L (95% CI: 229.05, 22.71 pmol/L; P = 0.02) (Thompson et al., 2017).
In a meta-analysis of 20 RCTs (N=607) fructan-type prebiotics reduced only LDL-C (MD – 0.15; 95% CI –0.29, –0.02; P = 0.03) without affecting the other endpoints. Within the

T2DM subgroup analysis, fructan supplementation was positively associated with a decreased fasting insulin concentration (MD: –4.01; 95% CI: –5.92, –2.09; P <0.0001) and increased high density lipoprotein-cholesterol (HDL-C) (MD: 0.07; 95% CI: 0, 0.14; P = 0.05). Moreover, fasting glucose tended to be reduced only in the T2DM subgroup (MD: –0.42; 95% CI: –0.90, 0.06; P =0.09) (Liu et al., 2016).
In meta-analyses of 35 RCTs the following was found: In patients with T2DM, psyllium (dosed before meals) significantly improved fasting blood glucose (FBG) concentration (237.0 mg/dL; P <0.001) and glycated hemoglobin (HbA1c) [20.97% (210.6 mmol/mol); P =0.048]. Glycemic effects were proportional to baseline FBG; no significant glucose lowering was observed in euglycemic subjects, a modest improvement was observed in subjects with pre-T2DM, and the greatest improvement was observed in subjects who were being treated for T2DM (Gibb et al., 2015).
In a meta-analysis of 25 studies (9 assessed prebiotic, 11 assessed probiotic, and 7 assessed synbiotic supplementation) for a total of 1309 patients with NAFLD the supplementation significantly reduced BMI (-0.37 kg/m^2; 95% CI -0.46 to -0.28; P <0.001), hepatic enzymes (ALT, -6.9 U/L [95%CI -9.4 to -4.3]; AST, -4.6 U/L [95%CI, -6.6 to -2.7]; c-GT, -7.9 U/L [95%CI, -11.4 to -4.4]; P <0.001), serum cholesterol (-10.1 mg/dL 95%CI, -13.6 to -6.6; P <0.001), LDL-C (-4.5 mg/dL; 95%CI, -8.9 to -0.17; P <0.001), and TAG (-10.1 mg/dL; 95%CI, -18.0 to -2.3; P <0.001), but not inflammation (TNF-a, -2.0 ng/mL; [95%CI, -4.7 to 0.61]; CRP, -0.74 mg/L [95%CI, -1.9 to 0.37]). Subgroup analysis by treatment category indicated similar effects of prebiotics and probiotics on BMI and liver enzymes but not total cholesterol, HDL-C, and LDL-C (Loman et al., 2018).

2.3 The pertinent lactobacilli

2.3.1 Origin

The Lactobacillus strains, used here, were isolated from Kimere, a spontaneously fermented pearl millet dough prepared in the Mbeere community of Kenya, East Africa (Njeru et al., 2010). It is fermented for 18-24 hours prior to consumption and is consumed in its active fermenting state. Kimere samples, collected from 11 homesteads in Mbeere, showed average pH values of 3.63 ± 0.29. Counts of presumptive lactic acid bacteria, aerobic mesophilic bacteria, and yeasts were 8.52 ± 0.02, 8.31 ± 0.04, and 7.19 ± 0.02 log10 colony forming units per gram, respectively. The yeast flora appeared to be dominated by Saccharomyces cerevisiae. Neither coliforms nor *Enterobacteriaceae* were detected in the Kimere samples.
48 *Lactobacillus* isolates were characterized and identified by Gram staining, catalase reaction, carbohydrate fermentation patterns, API 50 CHL, growth temperatures, species-specific polymerase chain reaction, amplified rDNA restriction analysis, and partial sequencing of the 16S rDNA. *Lactobacillus fermentum* (46 isolates) appeared to be the dominant *Lactobacillus* species in Kimere, with other species identified as *Lactobacillus plantarum* and *Weissella confusa* (1 isolate each). Analysis of strain diversity with pulsed-field gel electrophoresis indicated relatively large biodiversity among *L. fermentum* strains.
When tested for their potential to be applied as probiotics, all of the 46 *Lactobacillus fermentum* isolates were able to grow in MRS medium containing 0.3 % ox gall. 12 of them were able to grow in the presence and 3 %, and of these 60 % survived incubation at pH 3 in the presence of 2 mg pepsin per ml for three hours (Njeru et al., 2010).

2.3.2 Anti-inflammatory effects in vitro

From the 48 *Lactobacillus* strains isolated from Kimere 10 were selected based on their bile salt tolerance. Their effects on secretion by PBMCs of the T-helper cells Th1- and Th2-cytokines IFN-α and IL-4, respectively, in the presence or absence of staphylococcal enterotoxin A (SEA) were assessed (Ghadimi et al., 2014). Two strains, K1-Lb1 and K4-Lb6, induced basal IFNα-secretion. Four strains, K1-Lb6, K6-Lb2, **K7-Lb1**, and **K8-Lb1** diminished INFα-secretion by SEA-stimulated PBMCs. All strains, except K1-Lb1, K2-Lb4, and K9-Lb3, inhibited SEA-stimulated IL-4 secretion. Comparing the genomes of K1-Lb1 and K8-Lb1 by suppression subtractive hybridization (SSH) indicated that K1-Lb1 is able to synthetize polysaccharides, for the synthesis of which K1-Lb8 appears to lack enzymes. A difference in the hydrophobicity properties of the surfaces of both strains indicated that this has impact on their surface (Ghadimi et al., 2014).

2.3.3 Defensin-inducing properties in vitro

11 out of the 48 strains isolated from Kimere were tested for induction of the defensin hBD-2 by CaCo-2 cell (Ghadimi et al., 2011). Only two strains, **K11-Lb3** and K2-Lb6, significantly induced the production of hBD-2 by CaCo-2 cells. This effect was strain-specific, dose-dependent. The effect seemed to dependent on genes coding for glycosylated cell-surface structures synthesized with the aid of glycosyltransferase, UDP-N-acetylglucosamine 2-epimerase, and rod shape-determining protein MreC (Ghadimi et al., 2011). ß-Defensin 2 was shown to improve gut barrier function in dextran sodium sulfate (DSS)–induced colitis (Han et al., 2015).

2.3.4 Rational of selection of the pertinent strains

Based on these in vitro results and based on the impact of inflammation and intestinal barrier function on traits of the metabolic syndrome, the *L. fermentum* strains K7-Lb1 and K8-Lb1 (reducing Th1 and Th2 response) and K11-Lb3 (inducing defensin).

2.3.5 Rational of dosage of the pertinent strains

The dosage was adapted to the one used in most of the probiotics used for metabolic targets. It was also within the dose-response range of defensin induction of the *in vitro* trials published by Ghadimi et al., 2011 taking the ratio of lactobacilli per enterocyte *in vitro* into account and transferring this to the number of lactobacilli ingested and the average total number of **enterocytes in the human intestine.**

2.3.6 Safety

By documentation since the year 1901 the use of *L. fermentum* in fermented food has a long tradition (Bourdichon et al., 2012). The EFSA listed *L. fermentum* as one of the Qualified Presumption of Safety (QPS) microorganisms intentionally added to food (EFSA Journal, 2008). *L. fermentum* is also listed in the Inventory of the International Dairy Federation tested for safety (Bourdichon et al., 2012). The particular strains have a tradition of use as live microorganisms in fermented food (Njeru et al., 2010). Phenotypic evaluation of antibiotic resistance by Sacco laboratories (CENTRO SPERIMENTALE DEL LATTE S.r.l., specialised in production and testing microorganisms for food production) according to EFSA guidelines and ISO/IDF standard (EFSA Journal 2018) fulfilled the criteria required for their use in food (see attached documents). The strains are deposited at DSMZ (see attachment).

2.4 Acacia gum

2.4.1 Origin, chemistry and usage

Acacia gum = gum arabic is a natural gum consisting of the hardened sap of various species of the acacia tree. It is collected predominantly from *Acacia senegal* and *Vachellia (Acacia) seyal*.

Gum arabic is a complex mixture of glycoproteins and polysaccharides predominantly consisting of arabinose and galactose.

It is soluble in water, edible, and used primarily in the food industry as a stabilizer, with EU E number E414. Acacia gum is used in pharmacy and cosmetics as a binder, emulsifying agent, and a suspending or viscosity increasing agent (Smolinske, Susan C., 1992). It is used in the food industry as a stabilizer, emulsifier and thickening agent in icing, fillings, soft candy, chewing gum and other confectionery (Laura Halpin Rinsky; Glenn Rinsky, 2009) and to bind the sweeteners and flavorings in soft drinks, e.g., it is and ingredient in soft drink syrup (Cola) and "hard" gummy candies such as gumdrops, marshmallows, and M&M's.

2.4.2 Prebiotic effect (bifidogenicity, lactobacilli)

Acacia gum is a dietary fiber, which is not degraded by the human digestive enzymes in the small intestine, but fermented in the colon by the intestinal microorganisms. Acacia gum increased the counts of fecal bifidobacteria and lactobacilli in dosages of ≥ 10 g per day (Cherbut et al., 2003; Calame et al., 2008).

Therefore, it is more suited as synbiotic in combination with probiotic lactobacilli than FOS, which is mainly increasing bifidobacteria and causes abdominal complaints at these dosages, whereas acacia does not (Calame et al., 2008).

It is also more suited as synbiotic with lactobacilli than polydextrose, which increased bifidobacteria, but decreased lactobacilli (see table in do Carmo et al., 2016).

2.4.3 Effects on weight management

In mice Ushida et al., 2011 found reduced age-dependent fat deposition in the visceral adipose tissue by acacia gum compared to control. This was suggested to be due to β3-adrenergic stimulation of adipocytes.

According to this finding the total body weight gain was significantly decreased in mice treated with acacia gum (+10.97 ± 0.76 g) as compared to non-treated mice (+13.98 ± 0.98 g) (Nasir, 2014). In a further study acacia gum significantly decreased body weight ($P < 0.05$) and visceral adipose tissue weight ($P < 0.01$) (Musa et al., 2015). A high-fat diet supplemented with 10% w/w acacia gum (High+gum) for 12 weeks resulted in decreased ($P < 0.01$) visceral adipose tissue (VAT) compared to the control without acacia gum (Ahmed et al., 2016).

In a DB-RCT 120 healthy females were divided into two groups: A test group of 60 volunteers receiving acacia gum (30 g/day) for 6 weeks and a placebo group of 60 volunteers receiving pectin (1 g/day) for the same period of time. Fat percentage was calculated using Jackson and Pollock 7 caliper method and Siri equation. Pre- and post-analysis among the acacia gum group showed significant reduction in *BMI* by 0.32 (95% CI: 0.17 to 0.47; $P < 0.0001$) and *body fat* percentage by 2.18% (95% CI: 1.54 to 2.83; $P < 0.0001$) (Babiker et al., 2012).

In a DB-RCT 100 T2D patients were divided into two groups one receiving acacia gum (30 gm/day), the other 5 g of placebo daily for 3 months. BMI was decreased significantly by 2.06% (95% CI: −0.98; −0.16), $P < 0.05$) within the acacia gum group (Babiker et al., 2017).

In a DB-RCT 91 type 2 diabetic patients on hypoglycemic agents were assigned into two groups, either to consume 30 g of acacia gum or 5 g of placebo daily for 3 months. BMI and visceral adiposity index *VAI* decreased significantly ($P < 0.05$) in the acacia gum group by 2

and 23.7%, respectively. Body *adiposity index* significantly decreased by 3.9% in the acacia gum group while there were no significant changes in *waist circumference* or waist-to-hip ratio (WHR). Systolic blood pressure significantly decreased by 7.6% in acacia gum group and by 2.7% in placebo group from baseline (Babiker et al., 2018). A similar reduction of syst BP was seen in an uncontrolled study by Glover et al., 2009.
In an uncontrolled trial 52 T2D patients received 60 g/day of Gum arabic for three months. BMI was slightly but not significantly reduced during intervention (Ibrahim et al., 2017).

In summary the effects on body weight, BMI and visceral fat seem to be consistent. The effects, however, were only shown in before-after comparisons and not in comparisons with a proper placebo group. Furthermore, the study results were mainly provided by one center in Kartoum.

Since acacia gum promotes lactobacilli and seems to have metabolic effects by itself, we expect a synergistic or at least additive effect by combining the pertinent lactobacilli with acacia gum.

2.4.4 Effects on glucose metabolism

EFSA assessed the effects of acacia gum on postprandial glycemia and glucose maintenance in 2010 and concluded at that time that the effects were not sufficiently substantiated (EFSA, 2010). There, however, are findings which indicate effects and underlying mechanisms in mice and a potential effect in man.

In wild-type C57Bl/6 mice drinking a 20% glucose solution for four weeks significantly increased body weight and fasting plasma glucose concentrations. By acacia gum the effects were significantly blunted. Acacia gum further significantly blunted the increase in body weight, fasting *plasma glucose* and *fasting insulin* concentrations during high fat diet (Nasir et al., 2010). This may be explained by a downregulation of the Intestinal Na^+-coupled glucose transporter *SGLT1* (Nasir et al., 2010).
This is in agreement with the findings of Nasir et al., 2014: During acacia gum administration oral *glucose tolerance* with 3 mg/g bw was significantly improved compared to control mice (AUC 29700 ± 1018 min·mg/dl vs. 27207 ± 892 min·mg/dl) whereas intra-peritoneal glucose tolerance test was unaffected by supplementation with acacia gum.
Under prolonged administration of a high glucose diet and under prolonged administration of a high fat diet the fasting blood glucose was also blunted by simultaneous treatment with acacia gum as compared with the control group followed by a significant decrease in fasting insulin concentrations in mice receiving acacia gum as compared to the control without acacia gum (Nasir et al., 2014).
Gum arabic non-significantly reduced blood glucose and down-regulated PPAR-γ and SCD expression in mice (Musa et al., 2015).
In mice fed a high-fat diet (high) or a high-fat diet supplemented with 10% w/w acacia gum (High+gum) for 12 weeks blood glucose was decreased by acacia gum (Ahmed et al., 2016).

In the DB-RCT in T2D patients, mentioned above, the acacia gum group showed a significant reduction in fasting plasma glucose (FPG) and *HbA1c* (P<0.05) within the acacia gum group (Babiker et al., 2017).
In an uncontrolled trial in 49 T2D patient's acacia gum slightly, but n.s. reduced HbA1c (Ibrahim et al., 2017).

2.4.5 Effects on plasma lipids

In mice a reduction of *cholesterol and LDL-C levels*, respectively, was reported by Ahmed et al., 2016.
A reduction was also seen in human trials (Sharma, 1985; Ross et al., 1993; Mee et al., 1997; Mohamed et al., 2015; Babiker et al., 2017).
EFSA assessed the effect of acacia gum on cholesterol level in 2009 and concluded that the evidence was not sufficiently substantiated at that time.

2.4.6 Safety

Acacia gum is regarded as safe for human use as food ingredient. An ADI-value was not defined. Acacia gum, however, may seldomly elicit allergic reactions in individuals who are sensitive to acacia gum (https://www.lebensmittellexikon.de/g0002470.php). The specification of the acacia gum used in the test products can be seen from the attached technical data sheet from Nexira.

3 AIM

The effects of probiotics on glucose and lipid metabolism, on body fat, weight, visceral fat and liver steatosis were shown by several meta-analyses for the total variety, as described above. Some probiotic species/strains, however, seem to be more efficacious (Koutnikova et al., 2019). The lactobacilli used in this trial were selected for their anti-inflammatory properties and based on induction of defensins in enterocytes. Therefore, one may expect more pronounced effects of these strains on traits of the metabolic syndrome, which is driven by low grade inflammation, than those found in the meta-analyses for the whole variety of probiotics without discriminating species and strain specificity.
The combination of these *Lactobacillus* strains with acacia gum is expected to enable even more pronounce effects, since acacia gum was shown to increase the number of lactobacilli in the gut (Cherbut et al., 2003; Calame et al., 2008) and, hence, are supposed to promote their propagation and, hence their effects. The dosage of 10 g/day acacia gum was demonstrated to be sufficient for enhancing fecal lactobacilli and bifidobacterial (Cherbut et al., 2003; Calame et al., 2008).
Acacia gum, however, seems to have own effects on traits of the metabolic syndrome. Even though the effects still need to be confirmed in more DB-RCTs, one may suggest a separate effect, e.g. by reduction of the SGLT1in the intestine (Nasir et al., 2010).

This DB-RCT aims at providing first evidence for an effect of this symbiotic on traits of the metabolic syndrome. The target parameters were selected for allowing a health claim according to the Health Claim Directive of the EU (REGULATION (EC) No 1924/2006 OF THE EUROPEAN PARLIAMENT AND OF THE COUNCIL of 20 December 2006 on Nutrition and Health Claims Made on Foods) and/or the REGULATION (EU) No 609/2013 OF THE EUROPEAN PARLIAMENT AND OF THE COUNCIL of 12 June 2013 for Food for Special Medical Purposes after having confirmative evidence.
The following health claim options are feasible according to the EFSA Guidance on the scientific requirements for health claims related to appetite ratings, weight management, and blood glucose concentrations (EFSA Journal 2012;10(3):2604) and to the EFSA Guidance on the scientific requirements for health claims related to antioxidants, oxidative damage and cardiovascular health (EFSA Journal 2011;9(12):2474):

- Beneficial effect on long-term glycemia (glucose metabolism) as assessed by HbA1c,
- facilitates weight management as assessed by body weight, BMI, waist circumference, or body fat mass,
- reduces insulin resistance, a risk factor for type 2 diabetes, as assessed by HOMA-IR,
- reduces LDL-C, a risk factor coronary heart disease,
- increases HDL-C, a beneficial physiological effect.

It is noteworthy that EFSA admits demonstration of effects in type 2 diabetes for health claims on these target parameters assuming that there is a continuity of these parameters from healthy to impaired metabolism (EFSA Journal 2012;10(3):2604).

Alternatively, the following claims may be used as FSMPs:

- For dietary management of impaired glucose metabolism and type 2 diabetes
- for dietary weight management in overweight
- for dietary management of insulin resistance

The primary parameter has been selected by estimating the sample size for these potential targets based on the most recent meta-analysis of *Koutnikova et al. 2019*. Since we expect a more pronounced effect by the selected strains and the combination with acacia gum (see above) we assumed a twofold higher effect than found for the whole variety of probiotics. Taking this into account, the target parameter with the lowest estimated sample size was body fat in individuals with type 2 diabetes (N = 56 for each arm). Accordingly, this target was defined as primary parameter.

3.1 Study objectives and parameter

3.1.1 Primary target parameter

synbiotic vs placebo group (alteration V3-V1)

- Body Fat Mass (BFM) as assessed by bioelectrical impedance analysis (BIA)

3.1.2 Secondary target parameter

synbiotic vs placebo group (alteration V3-V1)

- HOMA-IR (Homeostasis Model Assessment (HOMA)-IR = glucose [mmol/L] x insulin [µU/ml]/22,5)

3.1.3 Exploratory parameters

synbiotic vs placebo group and probiotic vs placebo group (alteration V3-V1)

- Waist, Waist-to-Height Ratio (WHtR), body weight, BMI,
- MSX-index according to ATP III/IDF* definitions (Waist x FPG x Tg x 1/HDL-C x BP_{sys} x BP_{dias})(ascites as interfering cause for alteration of these measures will be excluded by abdominal sonography)

  **National Cholesterol Education Program Adult Treatment Panel (NCEP ATP III) and International Diabetes Federation (IDF)*
- HbA1c
- HOMA-IR (Homeostasis Model Assessment (HOMA)-IR = glucose [mmol/L] x insulin [µU/ml]/22,5)
- Blood pressure and pulse

- Blood parameters: FPG, insulin, Tg, HDL-C, LDL-C, hsCRP, AST, ALT, yGT, cytokeratin-18 fragment (markers of liver steatosis/steatohepatitis), gut permeability parameters: zonulin, LPS
- Fatty Liver Index (FLI) calculated from serum triglyceride, body mass index, waist circumference, and gamma-glutamyltransferase according to Bedogni et al. BMC Gastroenterol 2006; 6: 33: FLI = (e 0.953*loge (triglycerides) + 0.139*BMI + 0.718*loge (ggt) + 0.053*waist circumference - 15.745) / (1 + e 0.953*loge (triglycerides) + 0.139*BMI + 0.718*loge (ggt) + 0.053*waist circumference - 15.745) * 100
- NAFLD liver fat score according to Kotronen et al. Gastroenterology 2009; 137: 865-872: NAFLD liver fat score -2.89 + 1.18*metabolic syndrome (yes = 1/no= 0) + 0.45*type 2 diabetes (yes= 2/no= 0) + 0.15*fS-insulin (mU/L) + 0.04*fS-AST (U/L) - 0.94*AST/ALT
- Liver steatosis grade: Sonographical diagnosis of liver steatosis and quantification follows the criteria given by Saverymuttu et al. (1986) resulting in a grading from 0 = no steatosis, 1 = slight steatosis, 2 = moderate steatosis and 3 = severe steatosis.
- Sagittal abdominal diameter (SAD): Distance between the under surface of the rectus muscle and the anterior wall of the aorta – measure for visceral fat after *Armellini et al*. 1991: *Sagittal abdominal diameter as a practical predictor of visceral fat*
- anti-diabetic medication (X1*$dose_{max}$,); anti-hypertensive medication (X2*$dose_{max}$ + Y2*$dose_{max}$ + etc.); anti-lipidemic medication (X3*$dose_{max}$ + Y3*$dose_{max}$ + etc.) (Xi<1, Yi<1, maximal daily dose for each drug is assumed to be 1)
- Gastrointestinal Symptom Rating Scale (GSRS according to Svedlund 1988, Dimenäs 1995, Revicki 1998), related to the last 7 days before V1and V3
- compliance (counting consumed test products; Morisky score)
- values of the primary and secondary parameters at V1, V3

4 STUDY DESIGN AND POPULATION

4.1 Summary of study design

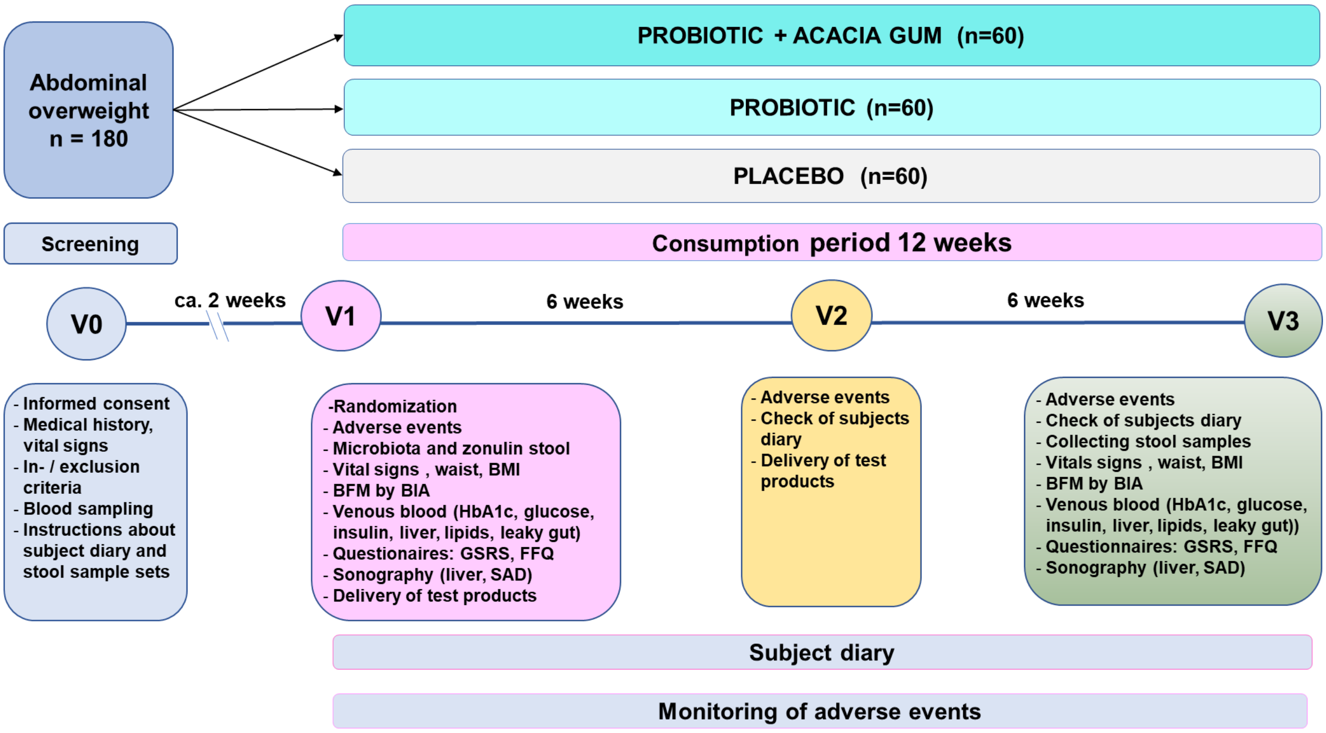

4.2 Description of the study design and conduct

This study follows a double-blind, randomized, placebo-controlled design with three parallel arms.

180 female and male overweight subjects aged ≥ 18 years complying with the inclusion and exclusion criteria listed below will be enrolled in the study. Subjects will be recruited from the database of the study site and in case of need from advertisements and flyers. The place of recruitment is Kiel and the surrounding area.

The Investigator should complete a subject screening log to document each subject screened for this study regardless of enrollment. This screening log also may state that subjects were chosen without bias.

The design enables the assignment of each subject to one of the three test products in a randomized order (s. section 7).

Subjects will appear in total for four visits during the study (detailed description of the visits see chapter 5).
Visit 0 (screening visit) and start of first intervention scheduled for visit 1 (intervention 1) should take place within ca. 2 weeks to provide actual lab parameters (e.g. impaired fasting glucose). The subsequent visits V2 und V3 will follow visit 1 after 6 weeks resp. 12 weeks.

4.2.1 Scheduled site visits

Visit	Type of visit	Time point
V0	Informed Consent, Evaluation of Inclusion & Exclusion criteria, Screening examination	
V1	Randomization, medical examinations and start of intervention	Around two weeks after V0
V2	Medical examinations	6 weeks ± 3 days after V1
V3	Medical examinations, end of study	12 weeks + 6 days after V1

Subjects will appear to all visits except V2 after a 12 h – overnight fast.

Subjects will be provided with a subject diary at V0 and will keep it until the end of the study. Subjects should complete the subject diary every day with regards to product consumption

- concomitant medication
- and possible events, which may be important information for the Investigator

Subjects will be required to bring the diary back to the study site at every visit for inspection.

4.3 Subject selection and withdrawal

The study population containing female and male subjects with abdominal obesity will be recruited from the CRC database receiving the announcement of the study via mail and, if necessary, by advertisement in the daily newspaper and flyers that are on display e.g. at the family doctor's waiting room.

They will contact the study site by phone or e-mail, if they are interested in further information. During the telephone contact subjects will receive summarized information about the planned trial. In case the subject is still interested in participation, he will be provided with the written subject information by e-mail or regular mail. After the subject has read the subject information, he will contact the study site to clarify any further questions and/or schedule a screening visit.

4.3.1 Inclusion criteria

To be enrolled, the following criteria have to be fulfilled:

1. Overweight or obese (BMI ≥ 25)
2. Elevated waist circumference (>94cm and >80cm (for European men and women, respectively)
3. Age ≥18
4. Written informed consent

4.3.2 Exclusion criteria

Any of the following is regarded as a criterion for exclusion from enrollment into the study:

20. Subjects currently enrolled in another clinical study
21. Subjects having finished another clinical study within the last 4 weeks before inclusion
22. Hypersensitivity, allergy or intolerance against any compound of the test products (e. g. acacia gum)
23. Condition after implantation of a cardiac pacemaker or other active implants
24. Sulfonylurea treatment
25. Any disease or condition which might compromise significantly the hepatic (ascites), hematopoietic, renal, endocrine, pulmonary, central nervous, cardiovascular, immunological, dermatological, gastrointestinal or any other body system with the exception of the conditions defined by the inclusion criteria
26. History of or present liver deficiency as defined by Quick < 70%
27. Regular medical treatment including OTC, which may have impact on the study aims (e. g. probiotics containing supplements, laxatives, steroids etc.)
28. History of hepatitis B, C, HIV
29. Major cognitive or psychiatric disorders
30. Subjects who are scheduled to undergo any diagnostic intervention or hospitalization which may cause protocol deviations
31. Simultaneous study participation by members of the same household
32. Pregnancy and lactation
33. Ascites as assessed by sonography
34. Any diet to lose body weight
35. Eating disorders or vegan diet
36. Anorexic drugs
37. Present drug abuse or alcoholism
38. Legal incapacity

4.3.3 Withdrawal or elimination criteria

No specific criteria for withdrawal of subjects are applied in the study. General procedures in case of premature discontinuation of subjects are described in the next chapter.

4.4 Premature discontinuation of the study

4.4.1 Subject‘s withdrawal

Subjects are free to withdraw from the study at any time without prejudice to their continued care.

4.4.2 Subject's discontinuation

Subjects may be discontinued from the study at any time. Specific reasons for discontinuing a subject from the study are:

- Safety reasons as judged by the Investigator
- Development of specific exclusion criteria during the study, which have impact on subject's safety
- Incorrect enrollment or randomization of the subject
- Subject's wish to withdraw prematurely from the study
- In case of a severe non-compliance to protocol as judged by the Investigator

Note: For subjects withdrawing prematurely from the study or have to discontinue due to any reason, adverse events should be followed up, questionnaires should be completed and diaries returned by the subjects, if possible.

4.4.3 Stop of a part or all of the study

If the study or part of the study is terminated or suspended for any medical or ethical reason or for any unexpected adverse events, the Investigator will inform the Ethics Committee.

4.5 Replacement conditions

- Randomized subjects being withdrawn prematurely before finishing all study visits will not be replaced unless the Sponsor decides on further recruitment in order to achieve the number of evaluable subjects as defined in the study protocol.
- Randomized subjects discontinuing prematurely before finishing all study visits will not be replaced unless the Sponsor decides on further recruitment in order to achieve the number of evaluable subjects as defined in the study protocol.

5 STUDY CONDUCT

5.1 Schedule of assessment

	**Visit 0** (Screening)	**Visit 1** (Randomization)	**Visit 2**	**Visit 3**
Written informed consent	X			
Demographic data	X			
Significant medical history	X			
Concomitant medication	X	X	X	X
Smoking and alcohol status	X			
Vital signs (blood pressure, pulse)	X	X		X
Body weight and height, waist	X	X		X
Arterial blood pressure, pulse	X	X		X
Blood samples (eligibility)	X			
Check of inclusion and exclusion criteria	X			

Subject's eligibility	X			
Allocation of a random number		X		
Visits at start and during intervention		X		X
Blood samples (HbA1c, glucose, insulin, lipids)		X		X
Blood samples (liver enzymes)		X		X
BIA, body fat mass		X		X
Sonography liver, HRI, SAD		X		X
Stool sample		X		X
Questionnaires on gastrointestinal symptoms (GSRS), with respect to the last 7 days before the visit day		X		X
Questionnaire on food frequency (FFQ)		X		X
Adverse event monitoring		X	X	X
Delivery of test products		X	X	
Accountability Log, compliance, Morisky[1] score			X	X[1]
Collection and checking of subject diary and questionnaires		X	X	X

6 STUDY VISITS

6.1 Screening visit (V0)

Prior to the inclusion procedure (for the assessment of eligibility of the subject), the subject will be informed in detail by written information as well as verbally by the investigator about the study and will be given the opportunity to ask the Investigator any questions. After signing and dating the Informed Consent Form (ICF) by both the subject and the Investigator the tasks performed during V0 are the following:

- Verification of subject identity
- Documenting the subject demographics and ethnics
- Taking the medical history
- Documenting possible concomitant medication (CM) and alimentary supplements
- Documenting smoking and alcohol status
- Ensuring subject is under 12-hours fasting condition
- Assessment of vital signs (blood pressure, pulse) and anthropometry (body weight, body height, waist circumference)
- Checking of the inclusion and exclusion criteria

- Blood sampling for eligibility/inclusion parameters (glucose, insulin, Na^+, K^+, creatinine, γ-GT, AST, ALT, AP, CHE, CRP, leukocytes, erythrocytes, Hb, hematocrit, MCV, MCH, MCHC, thrombocytes, LDL-C, HDL-C, triglycerides)
- Providing a subject diary for daily reply regarding adverse event and medication
- Providing 2 stool sampling kits for the stool samples to be collected within the lastb two days prior to V1 resp. V3
- Completing subject file and CRFs

6.2 Randomization and interventional visit 1 (V1)

During the second visit (V1) the study subjects will be randomized and the first dose of the study product will be consumed. This visit will take place around two weeks after V0. Possible adverse events which happened since V0 will be documented.

Tasks to be performed during V1:

- Assessment of adverse events since visit V1
- Checking of the inclusion / exclusion criteria
- Randomization
- Collecting of the first stool sample
- Assessment of arterial blood pressure, pulse, body weight and waist
- Blood sampling (glucose, Na^+, K^+, creatinine, γ-GT, AST, ALT, AP, CHE, CRP, leukocytes, erythrocytes, Hb, hematocrit, MCV, MCH, MCHC, thrombocytes, LDL-C, HDL-C, triglycerides, Quick (prothrombin time)
- Body composition analysis (**body fat mass**, lean body mass and other body composition parameters by means of bioelectrical impedance analysis (BIA).
- Sonography of liver and visceral fat (liver steatosis grade after *Saverymuttu et al. 1986* and *Webb et al. 2008,* exclusion of ascites, SAD – Sagittal Abdominal Diameter after *Armellini et al. 1991*) and exclusion of ascites.
- Subjects will complete the questionnaire on gastrointestinal symptoms (GSRS – Gastrointestinal Symptom Rating Scale; *Svedlund 1988*; *Dimenäs 1995*; *Revicki 1998*) with regard to the last 7 days before the visit day (V1)
- Subject will complete the EPIC Potsdam FFQ (Food Frequency Questionnaire) concerning the dietary intake in the last 12 months
- Dispensing of the first batch of test products for the intervention period (V1-V2)
- Consumption of the first dose of study product
- Instruction to bring back the unused test products and the subject diary at the next visit V2
- Completing subject file, eCRFs and accountability log.

6.3 Visit 2 (V2):

This visit will take place 6 weeks ± 3 days after V1. Subjects are requested to return the subject diary and the unused test product. Adverse events will be monitored.

Tasks to be performed during V2:

- Assessment of adverse events since visit V1
- Check of the subject diary
- Managing the returned test products and dispensing of the second batch of test products for the intervention period (V2-V3)
- Instruction to bring the unused test products, subject diary, the completed FFQ questionnaire and the froozen stool sample at the next visit V3
- Subjects are requested to attend the next visit V3 after an overnight fasting of at least 12 hours.
- Completing subject file, eCRFs and accountability log.

6.4 Visit 3 (V3)

The visit will take place at least 12 weeks after start of test product consumption (V1). Deviations in the date of V3 are permitted only up to 6 days after the scheduled visit date. The entire subject documentation will be checked and completed. The investigator will discuss all findings of blood withdrawals with the study participants.

The following tasks are to be performed at this final visit:

- Assessment of adverse events since visit V2
- Collecting of the second stool sample
- Assessment of arterial blood pressure, pulse, body weight and waist
- Check of the subject diary
- Blood sampling (HbA1c, glucose, insulin, triglyceride, HDL-C, LDL-C, CRP, ALT, AST, γ-GT)
- Body composition analysis (**body fat mass**, lean body mass and other body composition parameters) by means of bioelectrical impedance analysis (BIA)
- Sonography of liver and visceral fat; exclusion of ascites
- Subjects will complete the questionnaire on gastrointestinal symptoms (GSRS – Gastrointestinal Symptom Rating Scale) with regard to the last 7 days before the visit V3
- Subjects will complete the FFQ questionnaire concerning the dietary intake in the last 12 weeks (*only during intervention*)
- Assessment of subject compliance by means of pill counting (returned test products) and score after *Morisky et al.*
- Discussion about findings of blood withdrawals
- Handing over the expense allowance (crossed check)

7 TEST PRODUCTS

7.1 Synbiotic product components

The synbiotic product contains the strains *Lactobacillus fermentum* **K7-Lb1** (≥1x10^9CFU), *L. fermentum* **K8-Lb1** (≥1 x10^9CFU), *L. fermentum* **K11-Lb3** (≥1x10^9CFU) and **acacia gum** (gum arabic). To ensure identical characteristics with placebo, sucralose, cream flavour and maltodextrin are added too.

7.1.1 Lactobacillus fermentum strains

L. fermentum strains are produced by **CSL**: **C**ENTRO **S**PERIMENTALE DEL **L**ATTE S.R.L., I-26839 Zelo Buon Persico (LO), Strada Per Merlino 3, *Italy*, certified for production of bacterial freeze-dried cultures for food and farming/livestock, pharmaceutical and nutraceutical sectors, and of liquid yeast and mould cultures for the food industry. The company CSL has been assessed and complies with the requirements of FSSC 22000 (Certification scheme for food safety systems).

QPS status

The species *L. fermentum* are included in EFSA's **Q**uality **P**resumption of **S**afety (QPS) list.

Antibiotic susceptibility analysis

The strain has shown to be sensitive to antibiotics, following EFSA guidelines ISO/IDF standards (EFSA Journal, 2018, 16(3):5206; ISO 10932:2010; IDF 223:2010).

Microbiological specifications (all three *L. fermentum* strains)

**Assay**	**Result**	**Method (Reference)**
Yeast & mould	<10 CFU/g	CSLSOP CQ-099 (ISO 6611/IDF94)
Non-LacticAcid Bacteria	<5000 CFU/g	CSLSOP CQ-090 (ISO 13559)
*Bacillus cereus**	<100 CFU/g	CSLSOP CQ-089(ISO 7932)
Coagulasepositive staphylococci (including *Staphylococcus aureus*)*	Not detected in 1g	CSLSOP CQ-122 (ISO 6888-1-2)
Enterobacteriaceae	<10 CFU/g	CSLSOP CQ-105 (ISO 21528-1-2)
*Escherichia coli**	Not detected in 1g	CSLSOP CQ-104 (AFNOR BRD 07/01-07/93)
*Listeria monocytogenes**	Not detected in 25g	CSLSOP CQ-158 (AFNORBRD 07/04-09/98)
Salmonella spp.*	Not detected in 25g	CSLSOP CQ-184 (ISO 6785/IDF93)

* Analysed on regular basis. Allanalytical methods are available upon request

GMO Status

CSL organisms are not genetically modified (GMO), in accordance to the European Directive 2001/18/EC.

This product does not require labelling with regard to the use of GMO, in accordance to Regulation (EC) No.1829/2003, and Regulation (EC) No.1830/2003.

Allergens

The raw materials used are free of the following components and their products thereof: cereals containing gluten, crustaceans, eggs, fish, peanuts, soybeans, milk (including lactose), nuts, celery, mustard, sesame seeds, sulphur dioxide and sulphite, lupin and molluscs.

The list of allergens is in compliance with Regulation (EC) 1169/2011.

BSE/TSE status

This product does not contain bovine spongiform encephalopathy (BSE) or transmissible spongiformencephalopathies (TSE), in accordance to Regulation EMA 410/01 rev. 3.

Storage and shelf life
Cultures have a shelf-life
of 12 months when at or below +8 °C (46.6 °F).

7.1.2 Acacia gum

This component is a purified and instantised soluble dietary fibre and is produced by NEXIRA, 129 Chemin de Croisset – CS94151 – 76723 Rouen Cedex, *France.* It is a natural dietary fibre of the product line FIBREGUM ™ (FIBGRGUM P).

For more general information on acacia gum see section 2.4.1.

Technical Data Sheet (FIBREGUM P)

**Physical and chemical data**					
**Analysis**	**Description**	**MINI**	**MAXI**	**UNIT**	**Test Method**
Moisture	5h @ 105°C		10	%	USP <921> Method III
Colour @ 25%	Aqueous solution 25% - Lovibond AF900		7	–	–
pH	25% aqueous solution, @ 20°C	4,1	4,8	–	Eur. Ph. 2.2.3
Viscosity 25%	25%, Brookfield LVF 60 rpm @ 20°C	60	130	mPa.s	–
Total Ashes @ 600°C	8h @ 600°C		4	%	Eur. Ph.
Acid insoluble ashes	Ashes + acid hydrolysis		0,5	%	USP<561>
Acid insoluble matters	Gravimetric determination after acid hydrolysis		0,1	%	–
Optical rotation (1%)	589 nm, aqueous solution 1%	Laevorot.		–	–
Total dietary fibre (on dry weight)	Enzymatic & gravimétric	90		%	AOAC 985.29
Mesh size powder through 63 µm	Vibro - sieving		15	%	–
Turbidity 5%	5% Solution		120	NTU	–
Glucose and Fructose*	Chromatography	Pass test		–	HPLC
Starch, Dextrin and Agar*	Iodine solution test	Pass test		–	Eur. Ph.
Sterculia gum*	Eur. Ph (A-B)	Pass test		–	Eur. Ph.

Tragacantha*	Chromatography	Pass test		–	HPLC
Identification test*	Eur. Ph (A-B-C-D)	Pass test		–	Eur. Ph.
Tannin test*	reaction with ferric chloride solution	Pass test		–	Eur. Ph.
Solubility and reaction*	solution in water	Pass test		–	USP
Total heavy metal*			5	ppm	FCC (Method 2)
Arsenic*	ICP-Mass spectrometry		0,5	ppm	ICP -OES/ICP-MS
Lead*	ICP-Mass spectrometry		0,1	ppm	ICP -OES/ICP-MS
Mercury*	Atomic Absorption spectrometry		0,1	ppm	SAA / ICP-MS
Cadmium*	ICP-Mass spectrometry		0,1	ppm	ICP -OES/ICP-MS
**Bacteriology**					
**Analysis**	**Description**	**MINI**	**MAXI**	**UNIT**	**Test Method**
Total Plate Count	72 h @ 30°C - PCA		2000	cfu/g	ISO 4833-1
Total Coliform (test for the presence of)	24h LST 30°C + 24/48h BLBVB 30°C		abs/g	–	ISO 4831
Yeast	5 days @ 25°C - YGC		100	cfu/g	100 ISO 6611
Molds	5 days @ 25°C - YGC		100	cfu/g	ISO 6611
E. coli (test for the presence of)	48 h @ 44°C - EP without indole + Kovacs reagent		abs/5g	–	ISO 7251
Salmonella (presence)	Pre-enrichment, 24 h @ 41°C		abs/25g	–	ISO 6579-1

*tested 1/year (product of the same grade)

7.1.3 Maltodextrin

Maltodextrin is produced by ROQUETTE, 15063 Cassano Spinola (AL) – Via Serravalle 26, *Italy*. It is a mixture of glucose, disaccharides and polysaccharides obtained by the partial hydrolysis of starch.

7.1.4 Sucralose

Sucralose is produced by **Shandong** Kambo Biochemical Technology Co, Ltd, Shandong, *China* – 257400.

It functions as a nonnutritive sweetener/ flavour enhancer for e. g. tablets, pharmaceutical, powdered drink mixes, baking, carbonated soft drinks, yogurts, ice cream. Kanbo sucralose has a clean, sweet taste that is approximately 600 times sweeter than sugar. The product complies with FCC9, EP7.0, USP38 and E955 standards, according to Commission Regulation (EU) No 231/2012.

7.1.5 Cream flavour

Cream flavour (with trade name *Aroma Panna Dry*) is produced by ESSEPI, 20161 Milano – Via Bellerio 35, *Italy*.

The product obtained for mixing of flavouring substances is a fine powder white/strow yellow, with smell and taste characteristic. It is used in the pharmaceutical industry, Reg. CE 1334/2008 and has a shelf life of 1 year.

7.1.6 Composition of the synbiotic product (summary table)

**Ingredients**	***mg* per sachet**	**Intake**
Lactobacillus fermentum K7-Lb1 100 B/g	50	$1x10^9$ at the end of shelf life<br>$5x10^9$ at production
Lactobacillus fermentum K8-Lb1 100 B/g	50	$1x10^9$ at the end of shelf life<br>$5x10^9$ at production
Lactobacillus fermentum K11-Lb3 100 B/g	50	$1x10^9$ at the end of shelf life<br>$5x10^9$ at production
**Acacia gum**	5555,6	
Sucralose	11,3	
Cream flavour	97,4	
Maltodextrin	185,7	
**TOTAL**	6000	

7.2 Probiotic product components

The probiotic product contains of the same *L. fermentum* strains like the synbioticum *Lactobacillus fermentum* **K7-Lb1** (≥$1x10^9$CFU), *L. fermentum* **K8-Lb1** (≥$1x10^9$CFU), *L. fermentum* **K11-Lb3** (≥$1x10^9$CFU). To ensure identical characteristics with the symbiotic and the placebo product, microcrystalline cellulose (7.3.1). sucralose (7.1.4), cream flavour (7.1.5) and maltodextrin (7.1.3) are added too.

7.2.1 Lactobacillus fermentum strains

See 7.1.1

7.2.2 Composition of the probiotic product (summary table)

**Ingredients**	***mg* per sachet**	**Intake**
Lactobacillus fermentum K7-Lb1 100 B/g	50	$1x10^9$ at the end of shelf life<br>$5x10^9$ at production
Lactobacillus fermentum K8-Lb1 100 B/g	50	$1x10^9$ at the end of shelf life<br>$5x10^9$ at production

Lactobacillus fermentum K11-Lb3 100 B/g	50	$1x10^9$ at the end of shelf life<br>$5x10^9$ at production
**Microcrystalline cellulose**	5555,6	
Sucralose	11,3	
Cream flavour	97,4	
Maltodextrin	185,7	
**TOTAL**	6000	

7.3 Placebo product

7.3.1 Microcrystalline Cellulose

The main component of the placebo is microcrystalline cellulose (MC). MC is a connective agent added to prescription drugs, over the counter medications, and dietary supplements. Microcrystalline Cellulose is also known as cellulose. MC $(C_6H_{10}O_5)_n$ is refined wood pulp. It is a white, free-flowing powder. Chemically, it is an inert substance, is not degraded during digestion and has no appreciable absorption. In large quantities it provides dietary bulk and may lead to a laxative effect. MC used for placebo in this study is provided by produced by NUTRILINEA S.r.l , Italy, a company certified for design and production of food supplements (see secion 7.3).

7.3.2 Further components

The placebo contains the same amount of the component's maltodextrin, sucralose and cream flavor as the verum products (see above).

7.3.3 Composition of the placebo product (summary table)

**Ingredients**	***mg* per sachet**	**Intake**
Microcrystalline cellulose	5555,6	
Sucralose	11,3	
Cream flavour	97,4	
Maltodextrin	185,7	
**TOTAL**	6000	

Therefor all study test products are similar in smell, flavour, color, texture and appearance.

7.4 Production of test products

The test products are produced by NUTRILINEA S.r.I., Via Gran Bretagna 1 – 21013 Gallarate (VA), *Italy,* certified for design and production of food supplements on behalf of third parties. The company has been found to comply with the requirements of UNI EN ISO 9001:2015 (see appendix). It will be provided in cardboard boxes with double sachets. A double sachet is one unit with two sachets coupled by a perforation. So, the double sachet correspondents with the daily dosage as scheduled in this study.

On each **sachet** will be printed in the German language the following information:

- To be used in clinical trial
- Study code
- **Random-No**
- Batch number
- Expiry date
- Storage between 4°C and 8°C
- Stir in 200 ml non-alcoholic drink at room temperature 1minute before consumption

On each **cardboard box** a nonremovable label will be displayed in German language with the following information:

- To be used in clinical trial
- Study code
- Name of Sponsor
- Name of Investigator
- **Random-No**
- Batch number
- Expiry date
- Content: sachets 45 twin sachets
- Storage between 4°C and 8°C
- To be stirred in 200 ml non-alcoholic drink at room temperature
- Only for study participants

7.5 Mode of consumption

Each study participant will consume one sachet of 6 g test product twice daily, at morning and evening (one twin sachet daily).

7.6 Storing, hand out and return back of test products

The investigating site will ensure that the study products are stored safely and properly according to the instructions given by the Sponsor and kept in a secured location to which only the investigator and designated study staff have access.

Study products are dispensed once at visit V1 and once at visit V2. The first test product will be consumed in the study center at visit V1. At next study visits (V2 and V3), subjects must return any unused sachets to the person responsible for product management on study site.

7.7 Accountability of test products

The investigator must maintain an accurate record of the shipment and dispensing of study products in a product accountability log. Monitoring of product accountability will be performed by the quality manager after the visits and at the end of the trial.

8 SAFETY ASPECTS

8.1 Test products

8.1.1 Verum products

Safety information for *L. fermentum* (s. section 2.3. 6)

- - Heavy metal analysis

**Heavy metal***	**Amount (ppm)**
LEAD (Pb)	<1
MERCURY (Hg)	<0.03
CADMIUM (Cd)	<0.1

(*) level of heavy metals is controlled on regular basis

For more detailed information about safety for *L. fermentum* s. section 2.3.6 (Scientific background).

For more detailed information about safety for *acacia gum* s. section 2.4.6 in (Scientific background).

Other additives such as sucralose (E 955), flavorings and maltodextrin are also food additives that, in the given amounts, pose no health risk. (see Appendix I-III).

8.1.2 Placebo product

The main component of the placebo constitutes microcyistalline Cellulose (MC, E 460). It serves among other things as an indigestible fiber for reduced-calorie foods such as salad dressings, desserts and ice creams. In pharmacy it is used as a binder and carrier for the manufacture of tablets. Other additives such as sucralose (E 955), flavorings and maltodextrin are identical with the verum ingredients.

8.2 Bioelectrical impedance analyses (BIA)

The bioelectrical impedance analyses (BIA) is a commonly used method for assessment of body composition, in particular body fat (BFM), visceral fat mass and lean body mass (LBM).

A weak alternating current flows through the body and the voltage is measured in order to calculate impedance (resistance) of the body. Most of the body water is stored in the muscle, so the electric current through this tissue leads to lower impedance, whereas the fat tissues shows an increased impedance. This way, due to the different conducting tissue features the method can estimate the total body water (TBW), the fat-free body mass and the body fat.

In this study, the SECA mBCA 515 (medical Body Composition Analyser) will be used for the measurement of the primary parameter – Body Fat Mass (BFM). This is a precise and safe certified medical device which can measure within a short time (up to 17 seconds) the body fat in persons with a body weight up to 300 kg. This method is harmless and free of health risks. This measurement should not be performed on patients with pacemakers or other active implants.

8.3 Adverse events (AE's)

An adverse event is the appearance or worsening of any undesirable sign, symptom, or medical condition occurring in a subject even, if the event is not considered to be related to study products. Medical conditions/diseases present before starting study products are only considered adverse events, if they worsen after starting study products. Abnormal laboratory values or test results constitute adverse events only, if they induce clinical signs or symptoms, if they are considered clinically significant or require therapy.

The occurrence of adverse events should be sought by non-directive questioning of the subject at each visit during the study. Adverse events also may be detected when they are volunteered by the subject during or between visits or through physical examination, laboratory test, or other assessments. All adverse events must be recorded in the source and on the Adverse Events CRF with the following information:

- the severity grade (mild, moderate, severe)
- its relationship to the study products (suspected/not suspected)
- its duration (start and end dates or if continuing at final examination)
- the action taken
- whether it constitutes a serious adverse event (SAE)

An SAE is defined as an event which:

- is fatal or life-threatening
- results in persistent or significant disability/incapacity
- constitutes a congenital anomaly/birth defect
- requires inpatient hospitalization or prolongation of existing hospitalization, unless hospitalization is for:
  - routine treatment or monitoring of the studied indication, not associated with any deterioration in condition
  - elective or pre-planned treatment for a pre-existing condition that is unrelated to the indication under study and has not worsened since the start of study products
  - treatment on an emergency outpatient basis for an event not fulfilling any of the definitions of a SAE given above and not resulting in hospital admission

- social reasons and respite care in the absence of any deterioration in the patient's general condition
- is medically significant, i.e. defined as an event that jeopardizes the subject or may require medical or surgical intervention to prevent one of the outcomes listed above

Unlike routine safety assessments, SAEs are monitored continuously and have special reporting requirements; see Appendix II.

All adverse events should be treated appropriately. Treatment may include one or more of the following: no action taken (i.e. further observation only); study product dosage adjusted/temporarily interrupted; study product permanently discontinued due to this adverse event; concomitant medication given; non-products therapy given; patient hospitalized/patient's hospitalization prolonged. The action taken to treat the adverse event should be recorded on the Adverse Event CRF.

Once an adverse event is detected, it should be followed until its resolution or until it is judged to be permanent, and assessment should be made at each visit (or more frequently, if necessary) of any changes in severity, the suspected relationship to the study product, the interventions required to treat it, and the outcome.

8.3.1 Relationship of adverse event to the test product

The Investigator must determine the relationship of the AE to the test product under investigation and document this on the appropriate AE form. For each AE, an assessment of the relationship to the test product consumption should be made using the following scale:

- Not Related: no investigational product was taken or the AE can be ascribed with reasonable certainty to another cause.
- Unlikely: There are good reasons to think there is no relationship.
- Possible: Equal valid arguments can be considered for or against an implication of study product.
- Probable: The relationship is likely.
- Certain (definitely): There is strong relationship.

8.3.2 Reporting/Notification of adverse events

<u>Adverse events</u>

Details of all complaints reported spontaneously by subjects or observed by the Investigator or medical staff must be recorded in the AE report forms provided in the CRF. Each AE observed must be recorded separately.

The Investigator must report the following information on the AE Form; nature of the AE, time of onset, intensity, treatments, evolution, relationship to study procedure and study product, whether any action(s) were taken with respect to study product consumption, and finally the outcome for the subject. The intensity and the relationship to the study product will be evaluated based on the information stated above.

Any AE for which the Investigator considers that a link with the study product could reasonably be envisaged, will be considered to be a suspected adverse reaction(s).

Serious Adverse Events

All SAEs must be reported to the Sponsor and the producer of test products within 24 hours of the study site becoming aware of the problem. The Investigator should complete a SAE Report Form.

In the case whereby the SAE Report Form cannot be emailed within 24 hours, the Investigator may report the SAE via fax; however, a SAE Report Form must be completed as soon as possible after the informal report.

The Investigator will declare all SAEs which are likely to be due to the tested product and which occur during the course of the study, to the Ethical Committee (and Regulatory Authorities, if applicable) which gave their approval for the study. In the case of an event leading to death or endangered life, this declaration will be done without delay and within a maximum of 7 days from obtaining knowledge of the event. In the case of other unexpected serious reactions, the declaration will be made within a time limit of 15 days.

The Investigator will reply to all requests for further information concerning such events from the Authorities or the Committee.

8.3.3 Monitoring of persons following the occurrence of adverse events

The Investigator will monitor all AEs until a satisfactory resolution is obtained. Any clinical or biological examinations deemed necessary by the Investigating doctor will continue to be performed until a return to normal.

8.4 Blood pressure

Blood pressure will be measured at screening and at time 0 (before ingestion of the test product) and 120 min after the ingestion of the test product. Volunteers will only be randomized and eligible for intervention if the blood pressure is considered by the Investigator to be not clinically relevant/significant.

8.5 Monitoring of laboratory parameters

For all laboratory investigations the Investigator will receive actual printouts of the data of the subjects from Laboratory Dr. Krause and Colleagues, Kiel. A signed original printout will be attached to the subjects file and a copy thereof will be filed in the CRF. The reference ranges will be provided in the clinical trial manual (Investigator’s file) together with the descriptions of the laboratory methods used. The laboratory will provide normal ranges on each report to ease the Investigator’s assessment.

As part of the overall safety monitoring plan, the safety monitor (Prof. Dr. Schrezenmeir) will also assess the data, particularly those falling outside the normal range.

8.6 Keeping back up blood material

At visits 1 - 3 venous blood samples will be drawn to receive back up serum resp. plasma samples for repeated parameter measurements or additional analyses. After blood withdrawal the blood sample will be centrifuged at room temperature and 2600 g for 10 minutes. The resp.plasma will be divided into 2 Eppendorf tubes and aliquots will be stored frozen at -20°C until further analysis.

9 STATISTICAL METHODS

The statistical methodology will be described in detail in the statistical analysis plan (SAP), which will be prepared before locking the data base.

9.1 Determination of sample size

The primary parameter has been selected by estimating the sample size for several potential parameters (HbA1c, HOMA-IR, body weight, body fat mass, waist etc.) within different populations (with overweight, obesity, type 2 diabetes, IFG etc.) based on the most recent meta-analysis of the effects by probiotics (over all species and strains) published by *Koutnikova et al. 2019*. Since we expect a more pronounced effect by the selected strains to be used in this study and the combination with acacia gum (see above), we assumed a twofold higher effect size (measured with Cohen's d) than found for the whole variety of probiotics. Taking this into account, the target parameter with the lowest estimated sample size was body fat mass in individuals with overweight (N = 56 for each arm). Accordingly, this target was defined as primary parameter. Assuming a drop-out rate of 5-10% a target number of n=60 subject per group was determined.

9.2 Definition of sets to be analyzed

9.2.1 Intention-To-Treat (ITT) collective

The **Intention-To-Treat (ITT) collective**: all subjects randomized and having taken at least one dose of the test products (at V1).

Subjects will be evaluated in the planned treatment regimen rather than the actual treatment given.

9.2.2 Full Analysis (FAS) Set

Compliance with the ITT principle would necessitate complete follow-up of all randomized subjects for study outcomes. As this can't be achieved, a full analysis set (FAS), will be analysed. It is as complete as possible and as close as possible to the ITT-set (FAS) including all randomized test persons. Elimination of subjects is considered to be justified according to ICH E9 Guideline in the following cases:

- violation of an essential, before randomization objectively measurable inclusion criterion
- taking not a single dose of the test substance (without knowledge of the assigned test group)
- lack of any dates for the judgment of the effectiveness after randomization

The evidence for an effect will be examined assessing this FAS collective.

9.2.3 Per-Protocol (PP) Set

The Per-protocol (PP) Set: all subjects randomized, who have no major protocol deviation.

Additionally, a per-protocol analysis will be carried out to check the robustness of the product effect. For this analysis subjects, who could not be treated per protocol, are excluded.

9.3 Statistical tests

For the primary goal, the comparison of BFM between placebo versus synbiotic group Mann Whitney U test is applied. This is also applied for the secondary goals, unless study population is normally distributed. In this case the statistical evaluation will be performed with the two-sided *t* test.

The testing for normality will be performed with Kolmogorov-Smirnov test with Lilliefords correction.

Comparison within the groups will be performed for each parameter using two-sided statistical tests (parametric such as paired t test and/or non-parametric such as Wilcoxon Signed Rank Test according to the distribution).

Descriptive statistics will be given in mean, SD, 95% CI of mean, median, 25th and 75th percentiles, frequency of observation. In figures mean ± SEM will be used for depicting the results.

The statistical evaluation will be performed at a 5% significance level acc. to the ITT principle in the FAS as main target population. Evaluation in the ITT and PP population will serve as sensitivity analyses with regard to the primary and secondary parameters.

For the secondary parameters adjustment for multiple testing will be done according Bonferroni-Holm.

10 ETHICAL CONSIDERATIONS

The study will be performed in accordance with the principles of the current version (2013) of the Declaration of Helsinki (WMA Declaration of Helsinki – Ethical Principles for Medical Research Involving Human Subjects adopted by the 18th WMA (World Medical Association) General Assembly, Helsinki, Finland, June 1964, and amended for the last time by the 64th WMA General Assembly, Fortaleza, Brazil, October 2013, the recommendations for GCP (ICH E6) and the legislative and regulatory clauses in force in Germany (GCP-Verordnung).

10.1 Ethics Committee

Before starting the study, the Investigator will submit the protocol, the written ICF, and any other relevant documents to the appropriate Ethics Committee (IRB/IEC). Copies of IRB/IEC approvals should be sent to the sponsor of the study, as well as a list of the members of the committee and their qualifications. If applicable, the documents will also be submitted to the Health Authority in accordance with the local regulatory and legal requirements.

This study will be undertaken after approval from the Ethics Committee of the Medical Association of Schleswig-Holstein (Ärztekammer Schleswig-Holstein, Ethik-Kommission, Bismarckallee 8-12, 23795 Bad Segeberg/SH).

During the study course, change(s) in any aspect of the study, such as modification(s) of the protocol, written ICF and any other written information to be provided to subjects should be submitted to the Ethics Committee (IRB/IEC). If applicable, all updates of the Investigator Brochure will be supplied to Ethics Committee (IRB/IEC) as well.

Subject recruitment will start only after reception of a favourable opinion from the Ethics Committee.

10.2 Changes to the Study Protocol

The Local Ethics Committee will be notified of any amendments to the Clinical Study Protocol and no changes will be effected without approval from the Regulatory Authorities. During the study course, all amendments likely to increase the risks to subject and/or involve major modifications of the protocol, changes of the ICF and any other written information to be provided to subjects may only be considered valid after approval by the independent EC (IEC) by whom the study protocol was initially approved.

Subject recruitment will start only, but directly after reception of a favourable opinion from the Ethics Committee.

10.3 Protocol deviations

No deviation is tolerated systematically. Any deviation from the approved protocol should be documented and explained.

10.4 Informed consent form

The requirements of the research, the title, the objectives of the research, the detailed research protocol and the risks and constraints of the research associated with this study must be explained to each subject/patient both orally and in writing (subject information sheet) by the Investigator before the start of the study.

The subject must sign and date all pages of the written informed consent form (ICF) to take part in this study. The same is in force for the Investigator. The original of the signed and dated ICF retains by the Investigator and will be archived in the Investigator site file. A copy will be handed out to the subject. This procedure is under the responsibility of the Investigator.

Subjects may withdraw from the study at any time without having to provide justification. The confidentiality of medical data must be upheld.

Subjects’ identity must not be revealed to any official bodies. All information obtained during the course of the study shall be entered into the Investigators patient file (source data).

The ICF must be approved by the IRB/IEC and must be in compliance with ICH GCP, local regulatory requirements and legal requirements.

Any revised version of the ICF and written information should receive the IRB/IEC's approval/favourable opinion before any use.

11 DATA MANAGEMENT AND MONITORING

11.1 Identification of source documentation and data

11.1.1 Source Document Definition

Source documents are original documents or certified copies of the subject (e.g. hospital records, laboratory parameter, subjects' diaries, filled questionnaires or evaluation checklists, recorded data from automated instruments, photographic negatives, microfilm or magnetic media, x-rays, subject files etc.).

11.1.2 Source Data Definition

Source data is all information documented in original records (or certified copies of original records) of clinical findings, observations, or other activities in a clinical trial necessary for the reconstruction and evaluation of the trial.

11.1.3 Management of source documents during the study

The Investigator agrees to create and update the source documents throughout the duration of the study.

Principal action at inclusion:

The Investigator must ensure that there are specific documents or information which will allow:

- the certification of the identification of the subject
- the certification of the information regarding the consent and the exact references of the corresponding study
- the confirmation that the subject meets the criteria for inclusion (and, where possible, has no exclusion criterion)

During the entire duration of the study:

The source documents must include all the documents which enable the full completion of the CRF, in particular:

- Accurate information on intercurrent diseases
- A precise description of concomitant medication taken by the subject
- Reasons for unscheduled or unrealised visits
- Clinical and paraclinical documents which enable the evaluation of an adverse event and/or serious adverse event

At the end of the study:

At the end of the study, the Investigator will check all the source documents. In particular, the Investigator must check that there is confirmation that the subject has participated in the study.

The Investigator must indicate if the subject’s participation has been consistent with the protocol or, in the case of a breach to the protocol, stating the reason behind the breach and reason(s) for a premature withdrawal or exit from the study whether initiated by the producers, Investigator of by the subject himself.

11.2 Direct Access to Source Data/Documents

The Investigator will permit trial-related monitoring, audits and regulatory inspection(s) by providing direct access to source data/documents.

Subjects may have access to their personal data at the end of the research if he/she wishes. They must address their request to the Investigator in writing.

11.3 Layout of the case report form (CRF)

The CRFs will be electronic CRFs.

The model CRF will be prepared by tecura GmbH, Kiel. The final version will be approved by the Principal Investigator. All data collected during the study for each subject will be provided by the source documents (medical files). All data should be coherent with source documents, if otherwise, the difference should be justified and documented. Each CRF will be identified with the subject identification number (here ID- number).

11.4 Identification of subjects

A unique identifier (subject identification number) will be allocated to each subject in sequential order at the first visit to the Investigator’s site. This unique identifier will be used to protect the subject's identity and will always been used instead of the subject's name. It will be used throughout the duration of the study.

The CRFs will be pre-printed with the subject’s number, and this number should be written on all study documents for the corresponding subject.

11.5 Subject diary and questionnaires

The subject number will be written on the first page and in the header of each page of the diary and questionnaires, where entries by the subject are expected.

All information resulting in an AE or SAE will be firstly documented in the subject file and afterwards transcribed into the CRF (AE/SAE form). The subject diary and questionnaires are considered as source document.

11.6 Electronic data capture

The data in the eCRFs will undergo double data computer entry. Data managed by external or internal service providers will also be transferred on computer media.

11.7 Database Management

Data from the eCRFs are entered into the study database by CRC staff following their own internal standard operating procedures.

Subsequently, the entered data are systematically checked by Data Management staff. Obvious errors are corrected by Data Management personnel. Quality control audits of all key safety and efficacy data in the database are made prior to locking the database.

Laboratory samples will be processed centrally and the results will be sent electronically for further statistical evaluation.

The occurrence of any protocol deviations will be determined.

After the database has been declared to be complete and accurate, it will be locked and made available for data analysis.

12 ARCHIVAL

12.1 General

The following documents must be and will be archived for a minimum of 10 years after the completion of the study:

- Final version of the study protocol,
- Any forms containing protocol amendments,
- Written informed consent forms (Investigator only),
- Original CRFs,
- Ethics committee approval forms,
- All correspondence between the partners involved and the Investigator,
- Curriculum vitae (CV) of Investigators,
- Acknowledgements of receipt and study product accountancy forms as well as all other documents generated during the study.

12.2 Investigator

All documents concerning this study must be kept by the Investigator: subject's medical files, the informed consent form, source documents, original of CRF, accounting forms for products administered, ethics committee approval and correspondence with the partners involved in the study.

The Investigator agrees to provide direct access to source documents during monitoring visits.

These documents are kept at the Investigator's site for at least 10 years after the completion of the study.

13 CONFIDENTIALITY

All information collected during the study is considered confidential and must not be disclosed without prior agreement of the Sponsor. The identity of study subjects must not be revealed to the Sponsor.

14 INSURANCE

The Principal Investigator has an insurance policy with an established insurance company in accordance with current regulatory requirements in Germany to cover its liability and the liability of the personnel involved in this study in the event of damage or injury resulting from the research project.

15 OWNERSHIP OF RESULTS

All data and results collected from the study are the proprietary of the Sponsor. The study results may be published or presented by the Investigator and/or by experts involved in the study, in collaboration and with the written approval of the Sponsor of the latter. The Sponsor may use the results of the study for publications or communications with the written agreement of the Investigator or experts if the latters are cited.

16 STUDY REPORT AND PUBLICATION

Following analysis of the study data, a final study report will be prepared describing the conditions under which the study was performed and the results. This report will be prepared and signed by the Sponsor's representative and the Principal Investigator. The results of the study may be published in a peer-reviewed international journal.

17 RESPONSIBILITIES

17.1 Sponsor

- The Sponsor must provide the Investigator with all documents required to provide information on the study products. In particular, the Sponsor must provide the Investigator with a certificate that the study products are appropriate for human nutrition.
- The Sponsor may decide to cancel the study at any time.
- Archiving the Sponsor documents for the legally required duration of 10 years.

17.2 Investigator

The Investigator is responsible for the following tasks:

- Submission of the study protocol to the local IEC before the start of the study.
- Taking out a specific trial insurance policy for the study and providing the subjects with insurance certification.
- Providing oral information and written subject information for participants.
- Confidentiality regarding all personal data of subjects.
- Verification and data entry in the electronic CRFs.
- Management of study products.

Before the study, provision of the following documents to the partners involved in the study: Ethics committee approval of the study protocol and study related documents:

- Conducting of the clinical study within the planned timeline
- Reporting of serious adverse events and adverse events.
- Meeting CRAs at the monitoring visits.
- Preparing study report.
- Archiving of study documents for 10 years.

18 REFERENCES

Ahmed, A.A., Musa, H.H., Fedail, J.S., Sifaldin, A.Z., & Musa, T.H.; Gum arabic suppressed diet-induced obesity by alteration the expression of mRNA levels of genes involved in lipid metabolism in mouse liver, Bioactive Carbohydrates and Dietary Fibre, Vol. 7, Issue 1, January 2016, Pages 15-20

Armellini, F., Zamboni, M., Rigo, L., Bergamo-Andreis, I.A., Robbi, R., de Marchi, M., & Bosello, O.; Sonography detection of small intra-abdominal fat variations., Int J Obes. 1991 Dec;15(12):847-52

Babiker, R., Merghani, T.H., Elmunsharaf, K., Badi, R.M., Lang, F., & Saeed, A.M.; Effects of gum Arabic ingestion on body mass index and body fat percentage in healthy adult females: two-arm randomized, placebo controlled, double-blind trial, Nutrition Journal 2012, 11:111

Babiker, R., Elmusharaf, K., Keogh, M.B., Banaga, A.S.I., & Saeed, A.M.; Metabolic effects of Gum Arabic (Acacia Senegal) in patients with Type 2 Diabetes Mellitus (T2DM): Randomized, placebo controlled double blind trial, Functional Foods in Health and Disease 2017; 7(3): 219-231

Babiker, R., Elmusharaf, K., Keogh, M.B., & Saeed, A.M.; Effect of Gum Arabic (Acacia Senegal) supplementation on visceral adiposity index (VAI) and blood pressure in patients with type 2 diabetes mellitus as indicators of cardiovascular disease (CVD): a randomized and placebo-controlled clinical trial, Lipids in Health and Disease (2018) 17:56

Beserra, B.T.S., Fernandes, R., do Rosario, V.A., Mocellin, M.C., Kuntz, M.G.F., & Trindade, F.B.S.M.; A systematic review and meta-analysis of the prebiotics and synbiotics effects on glycaemia, insulin concentrations and lipid parameters in adult patients with overweight or obesity, Clin. Nutr. 2015 Volume 34, Issue 5, Pages 845–858

Bourdichon, F., Berger, B., Casaregola, S., Farrokh, C., Frisvad, J.C., Gerds, M.L., Hammes, W.P., Harnett, J., Huys, G., Laulund, S., Ouwehand, A., Powell, I.B., Prajapati, J.B., Seto, Y., Schure, E.T., van Boven, A., Vankerckhoven, V., Zgoda, A., & Hansen, E.B.; Safety Demonstration of Microbial Food Cultures (MFC) in Fermented Food Products, Bulletin of the IDF 2012; 455: 1-68

Calame, W., Weseler, A.R., Viebke, Ch., Flynn, C., & Siemensma, A.D.; Gum arabic establishes prebiotic functionality in healthy human volunteers in a dose-dependent manner, British Journal of Nutrition (2008), 100, 1269–1275

do Carmo, M.M.R., Walker, J.C.L., Novello, D., Caselato, V.M., Sgarbieri, V.C., Ouwehand, A.C., Andrello, N.A., Hiane, P.A., & dos Santos, E.F.; Polydextrose: Physiological Function, and Effects on Health, Nutrients 2016, 8, 553; doi:10.3390/nu8090553

Le Chatelier, E., Nielsen, T., Qin, J., Prifti, E., Hildebrand, F., Falony, G., Almeida, M., Arumugam, M., Batto, J.-M., Kennedy, S., Leonard, P., Li, J., Burgdorf, K., Grarup, N., Jørgensen, T., Brandslund, I., Nielsen, H.B., Juncker, A.S., Bertalan, M., Levenez, F., Pons, N., Rasmussen, S., Sunagawa, S., Tap, J., & Tims,S.; Richness of human gut microbiome correlates with metabolic markers; Nature 500, 541–546 (29 August 2013) doi:10.1038/nature12506

Cherbut, Ch., Michel, C., Raison, V., Kravtchenko, T., & Severine, M.; Acacia Gum is a Bifidogenic Dietary Fibre with High Digestive Tolerance in Healthy Humans, Microbial Ecology in Health and Disease 2003; 15: 43 /50

Claesson, M.J., Jeffrey, I.B., Conde, S., Power, S.E., O'Connor, E.M., Cusack, S., Harris, H.M.B., Coakley M., Lakshminarayanan, B. O'Sullivan, O., Fitzgerald, G.F., Deane, J., O'Connor M., Harnedy, N., O'Connor, K., O'Mahony, D., van Sinderen, D., Wallace, M., Brennan, L., Stanton, C., Marchesi, J.R., Fitzgerald, A.P., Shanahan, F., Hill, C., Ross, R.P., & O'Toole P.W.; Gut microbiota composition correlates with diet and health in the elderly; Nature 9 August 2012; Vol. 488: 178-184; doi:10.1038/nature11319

Cotillard, A., Kennedy S.P., Kong L.Ch., Prifti, E., Pons N., Le Chatelier, E., Almeida, M., Quinquis, B., Leverenz, F., Galleron, N., Gougis, S., Rizkalla, S., Batto, J.-M., Renault, P., ANR MicroObes consortium, Dore, J., Zucker, J.-D., Clement, K., &Ehrlich, S.D.; Dietary intervention impact on gut microbial gene richness; Nature 29 August 2013, 500: 585-590; doi:10.1038/nature12480

David, L.A., Maurice, C.F., Carmody, R.N., Gootenberg, D.B., Button, j.E., Wolfe, B.E., Ling, A.V., Devlin, A.S., Varma, Y., Fischbach, M.A., Biddinger, S.B., Dutton, R.J., & Turnbaugh, P.J.; Diet rapidly and reproducibly alters the human gut microbiome, Nature. 2014 January 23; 505(7484): 559–563. doi:10.1038/nature12820

Devkota, S., Wang, Y., Musch, M., Leone, V., Fehlner-Peach, H., Nadimpalli, A., Antonopoulos, D.A., Jabri, B., & Chang, E.B.; Dietary fat-induced taurocholic acid production promotes pathobiont and colitis in IL-10 -/- mice, Nature. 2012 July 5; 487(7405): 104–108. doi:10.1038/nature11225

Dimenäs, E., Glise, H., Hallerbäck, B., Hernqvist, H., Svedlund, J., & Wiklund, I.; Well-Being and Gastrointestinal Symptoms among Patients Referred to Endoscopy Owing to Suspected Duodenal Ulcer, Scand J Gastroenterol. 1995 Nov;30(11):1046-52

EFSA Panel on Biological Hazards (BIOHAZ), 2008. Scientific Opinion of the Panel on Biological Hazards on the maintenance of the list of QPS microorganisms intentionally added to food or feed. The EFSA Journal 2008, 923, 1-48.

EFSA Panel on Dietetic Products, Nutrition and Allergies (NDA), Scientific Opinion on the substantiation of health claims related to acacia gum (gum arabic) and reduction of post-prandial glycaemic responses (ID 842, 1977) and maintenance of normal blood glucose concentrations (ID 842, 1977) pursuant to Article 13(1) of Regulation (EC) No 1924/2006 EFSA Journal 2010; 8(2):1475

EFSA Panel on Biological Hazards (BIOHAZ), 2011a. Scientific Opinion on the maintenance of the list of QPS biological agents intentionally added to food and feed (2011 update). EFSA Journal 2011;9(12):2497, 82 pp. doi:10.2903/j.efsa.2011.2497

EFSA Panel on Dietetic Products, Nutrition and Allergies (NDA); Guidance on the scientific requirements for health claims related to appetite ratings, weight management, and blood glucose concentrations. EFSA Journal 2012;10(3):2604. [11 pp.]. doi:10.2903/j.efsa.2012.2604

EFSA Panel on Additives and Products or Substances used in Animal Feed (FEEDAP), Guidance on the characterisation of microorganisms used as feed additives or as production organisms, The EFSA Journal 2018;16(3): 5206; ISO 10932:2010; IDF 223:2010

Elshaghabee, F.M.F, Bockelmann, W., Meske, D., de Vrese, M., Walte, H.-G., Schrezenmeir, J., & Heller, K.J.; Ethanol Production by Selected Intestinal Microorganisms and Lactic Acid Bacteria Growing under Different Nutritional Conditions, Frontiers in Microbiology, January 2016, Vol. 7, Article 47, doi: 10.3389/fmicb.2016.00047

Elshaghabee, F.M.F., Rokana, N., Panwar, H., Heller, K.J., & Schrezenmeir, J.; Probiotics for dietary management of non‑alcoholic fatty liver disease, Environmental Chemistry Letters 2019; doi.org/10.1007/s10311-019-00896-8

Everard, A., Belzer, C., Geurts, L., Ouwerkerk, J.P., Druart, C., Bindels, L.B., Guiot, Y., Derrien, M., Muccioli, G.G., Delzenne, N.M., de Vos, W.M., & Cani, P.D.; Cross-talk between Akkermansia muciniphila and intestinal epithelium controls diet-induced obesity, PNAS, May 28, 2013, Vol. 110 No. 22 9066-9071

Everad, A., Cani, P.D.; Diabetes, obesity and gut microbiota, Elsevier Ltd. 2013, 1521-6918, http://dx.doi.org/10.1016/j.bpg.2013.03.007

Falony, G., Joossens, M., Vieira-Solva, S., Wang, J., Darzi, Y., Faust, K., Kurilshikov, A., Bonder, M.J., Valles-Colomer, M., Vandeputte, D., Tito, R.Y., Chaffron, S., Rymenans, L., Verspecht, C., de Sutter, L., Lima-Mendez, G., D'hoe, K., Jonckheere, K., Homola, D., Garcia, R., Tigchelaar, E.F., Eeckhaudt, L., Fu, J., Henckaerts, L., Zhernakova, A., Wijmenga, C., & Raes, J., Population-level analysis of gut microbiome variation, Science 2016; 352: 560-64; doi:10.1126/science.aad3503

Flandroy, L., Poutahidis, Th., Berg, G., Clarke, G., Dao, M.-C., Decaestecker, E., Furman, E., Haahtela, T., Massart, S., Plovier, H., Sanz, Y., & Rook, G.; The impact of human activities and lifestyles on the interlinked microbiota and health of humans and of ecosystems, Science of the Total Environment 627 (2018) 1018–1038, https://doi.org/10.1016/j.scitotenv.2018.01.288

Ghadimi, D., Fölster-Holst, R., de Vrese, M., Winkler, P., Heller, K.J., & Schrezenmeir, J.; Effects of probiotic bacteria and their genomic DNA on TH1/TH2-cytokine production by peripheral blood mononuclear cells (PBMCs) of healthy and allergic subjects Immunobiology 213 (2008) 677–692; doi: 10.1016/j.imbio.2008.02.001

Ghadimi, D., Hasssan, M., Njeru, P.N., de Vrese, M., Geis, A., Shalabi, A.I., Abdel-Razek, S.T., Abdel-Khair, A.A., Heller, K.J., & Schrezenmeir, J.; Suppression subtractive hybridization identifies bacterial genomic regions that are possibly involved in hBD-2 regulation by enterocytes, Mol. Nutr. Food Res. 2011, 55, 1533–1542; doi:10.1002/mnfr.201100052

Ghadimi, D., Njeru, P.N., Guigas, C., Farghaly, M.H., Fölster-Holst, R., Geis, A., de Vrese, M., Schrezenmeir, J., & Heller K.J.; Molecular identification of potential Th1/Th2 responses-modulating bacterial genes using suppression subtractive DNA hybridization, Immunobiology 219 (2014) 208– 217; http://dx.doi.org/10.1016/j.imbio.2013.10.005

Gibb, R.D., McRorie (Jr.), J.W., Russell, D.A., Hasselblad, V., & D'Alessio, D.A.; Psyllium fiber improves glycemic control proportional to loss of glycemic control: a meta-analysis of data in euglycemic subjects, patients at risk of type 2 diabetes mellitus, and patients being treated 1 for type 2 diabetes mellitus, AJCN 2015;102:1604–14

Gibson, G.R., & Roberfroid, M.B.; Dietary Modulation of the Human Colonie Microbiota: Introducing the Concept of Prebiotics, J. Nutr. 1995; 125: 1401–12

Glover, D.A., Ushida, K., Philips, A.O., & Riley, S.G; Acacia(sen) SUPERGUM™ (Gum arabic): An evaluation of potential health benefits in human subjects, Food Hydrocolloids, Vol. 23, Issue 8, December 2009, Pages 2410-2415, https://doi.org/10.1016/j.foodhyd.2009.06.020

Han,F., Haiwen Zhang,H., Xi Xia,X., Xiong, H., Song,D., Zong,X., Wang, Y. Porcine ß-defensin 2 attenuates inflammation and mucosal lesions in dextran sodium sulfate–induced colitis. J. Immunology, 2015, 194: 1882–1893

Hu, Y., Zhou, F., Yuan, Y., Xu, Y.; Effects of probiotics supplement in patients with type 2 diabetes mellitus: A meta-analysis of randomized trials,. Medicina Clínica, Volume 148, Issue 8, 21 April 2017, Pages 362-370, http://doi.org/10.1016/j.medcle.2017.03.003

Ibrahim, N.M., Ali, A.M., Khogali, N.A.R., & Bashir, H.S.; Effect of Gum Arabic as Dietary Supplement for Type II Diabetes in Jabir Abu Aliz Diabetic Center- (Khartoum State), Global Journal of Health Science; Vol. 9, No. 7; 2017

Kellow, N.J., Coughlan, M.T., & Reid, Ch.M.; Metabolic benefits of dietary prebiotics in human subjects: a systematic review of randomised controlled trials, British Journal of Nutrition, Vol. 111, Issue 07, April 2014, pp 1147-1161

Koutnikova, H., Genser, B., Monteiro-Sepulveda, M., Faurie, J.-M., Rizkalla, S., Schrezenmeir, J., & Clément, K.; Impact of bacterial probiotics on obesity, diabetes and non-alcoholic fatty liver disease related variables: a systematic review and meta-analysis of randomised controlled trials, BMJ Open 2019;9: e017995. doi:10.1136/bmjopen-2017-017995

Liu, F., Prabhakar, M., Ju, J., Long, H., & Zhou, H.-W.; Effect of inulin-type fructans on blood lipid profile and glucose level: a systematic review and meta-analysis of randomized controlled trials, EJCN 2016: 1–12

Loman, B.R., Hernàndesz-Saavedra, An, R., & Rector, S.; Prebiotic and probiotic treatment of nonalcoholic fatty liver disease: a systematic review and meta-analysis, Nutrition Reviews Vol. 76(11):822–839

Ma, Y.-Y., Li, L., Yu, Ch.-H., Shen, Z., Chen, L.-H., & Li, Y.-M.; Effects of probiotics on nonalcoholic fatty liver disease: A meta-analysis, World J Gastroenterol 2013 October 28; 19(40): 6911-6918, doi:10.3748/wjg.v19.i40.6911

Mee, K.A., & Gee, D.L.; Apple fibre and gum arabic lowers total and low-density lipoprotein cholesterol levels in men with mild hypercholesterolemia, J Am Diet Assoc. Vol. 97 No. 4, 422-4, April 1997

Mohamed, R.E., Gabour, M.O., & Adam, I.; The lowering effect of Gum Arabic on hyperlipidemia in Sudanese patients, Frontiers in Physiology May 2015, Vol. 6, Article 160, doi: 10.3389/fphys.2015.00160

Morisky, D.E., Green, L.W., & Levine, D.M.; Concurrent and Predictive Validity of a Self-reported Measure of Medication Adherence, Medical Care, January 1986, Vol. 24, No. 1, pages 67-74

Musa, H.H., Ahmed, A.A., Musa, T.H., & Fedail, J.S.; Gum arabic down-regulate PPAR-γ and SCD mRNA expression in mice, Polish Annals of Medicine, Vol. 22, Issue 1, June 2015, Pages 11-17, https://doi.org/10.1016/j.poamed.2015.03.008

Nasir, O., Artunc, F., Wang, K., Rexhepaj, R., Föller, M., Ebrahim, A., Kempe, D.S., Biswas, R., Bhanduru, M., Walter, M., Mohebbi, N., Wangner, C.a., Saeed, A.M., & Lang, F.; Downregulation of Mouse Intestinal Na -coupled Glucose Transporter SGLT1 by Gum Arabic (Acacia Senegal), Cellular Physiology and Biochemistry, Cell Physiol Biochem 2010;25:203-210

Nasir, O.; Effect of Gum Arabic (Acacia Senegal) on Glucose Metabolism and Body Weight Gain in Mice, Journal of Biology, Agriculture and Healthcare, Vol.4, No.9, 2014

Nikbakht, E., Khalesi, S., Singh, I., Williams, L.Th., West, N.P., & Colson, N.; Effect of probiotics and synbiotics on blood glucose: a systematic review and meta-analysis of controlled trials, Eur J Nutr 2016, doi:10.1007/s00394-016-1300-3

Njeru, P.M., Rösch, N., Ghadimi, D., Bockelmann, W., de Vrese, M., Schrezenmeir, J., & Heller, K.J.; Identification and characterization of lactobacilli isolated from Kimere, a spontaneously fermented pearl millet dough from Mbeere, Kenya (East Africa), Beneficial Microbes: 2010; 1: 243 – 252; doi:10.3920/BM2010.0019

Qin, J., Li, Y., Cai, Z., Li, S., Zhu, J., Zhang, F., Liang, S., Zhang, W., Guan, Y., Shen, D., Peng, Y., Zhang, D., Jie, Z., Wu, W., Qin, Y., Xue, W., Li, J., Han, L., Lu, D., Wu, P., Dai, Y., Sun, X., Li, Z., Tang, A., Zhong, S.; A metagenome-wide association study of gut microbiota in type 2 diabetes Nature 490, 55–60 (04 October 2012)

Revicki, D.A., Wood, M., Wiklund, I., & Crawley, J.; Reliability and validity of the gastrointestinal symptom rating scale in patients with gastroesophageal reflux disease, Quality of Life Research, 1998, Vol. 7, pp. 75–83

Rinsky, L.H., Rinsky, G. (2009). The Pastry Chef's Companion: A Comprehensive Resource Guide for the Baking and Pastry Professional. Chichester: John Wiley & Sons. pp. 1, 134. ISBN 978-0-470-00955-0

Ross, A.H.M., Eastwood, M.A., Brydon, W.G., Anderson, J.R., & Anderson, D.M.W.; A study of the effects of dietary gum arabic in humans, The American Journal of Clinical Nutrition, Vol. 37, Issue 3, March 1983, Pages 368–375, https://doi.org/10.1093/ajcn/37.3.368

Ruan, Y., Sun, J., He, J., Chen, F., Chen, R., & Chen, H.; Effect of Probiotics on Glycemic Control: A Systematic Review and Meta-Analysis of Randomized, Controlled Trials; PLoS One July 10, 2015;10:e0132121, doi:10.1371/journal.pone.0132121

Saverymuttu, S.M., Joseph, A.E.A., & Maxwell, J.D.; Ultrasound scanning in the detection of hepatic fibrosis and steatosis, BMJ Vol. 292, 4 January 1986

Schrezenmeir, J., & de Vrese, M.; Probiotics, prebiotics, and synbiotics - approaching a definition. AJCN 2001;73 (suppl):361S–4S

Sharma, R.D.; Hypocholesterolemic effect of gum acacia in men, Nutrition Research, Volume 5, Issue 12, December 1985, Pages 1321-1326, https://doi.org/10.1016/S0271-5317(85)80042-7

Smolinske, S.C. (1992). Handbook of Food, Drug, and Cosmetic Excipients. p. 7. ISBN 0-8493-358

Sun, J., & Buys, N.; Effects of probiotics consumption on lowering lipids and CVD risk factors: A systematic review and meta-analysis of randomized controlled trials, Ann Med 2015;47:6,430-40, doi:10.3109/07853890.2015.1071872

Svedlund, J., Sjödin, I., & Dotevall, G.; GSRS--A Clinical Rating Scale for Gastrointestinal Symptoms in Patients with Irritable Bowel Syndrome and Peptic Ulcer Disease, Digestive Diseases and Sciences, Vol. 33, No. 2 (February 1988), pp. 129-134

Thompson, S.V., Hannon, B.A., An, R., & Holscher, H.D.; Effects of isolated soluble fiber supplementation on body weight, glycemia, and insulinemia in adults with overweight and obesity: a systematic review and meta-analysis of randomized controlled trials, AJCN 2017;106:1514–28

Ushida, K., Hatanaka, H., Tsukahara, R.T., & Philips, G.O.; Effect of long term ingestion of gum arabic on the adipose tissues of female mice, Food Hydrocolloids, Vol. 25, Issue 5, July 2011, Pages 1344-1349, https://doi.org/10.1016/j.foodhyd.2010.12.010

Webb, M., Yeshua, H., Zelber-Sagi, S., Santo, E., Brazowski, E., Halpern, Z., & Oren, R.; Diagnostic Value of a Computerized Hepatorenal Index for Sonographic Quantifcation of Liver Steatosis, AJR:19 2, April 2009, doi:10.2214/AJR.07.4016

Wu, G.D., Chen, J., Hoffmann, Ch., Bittinger, K., Chen, Y.-Y., Keilbaugh, S.A., Bewtra, M., Knights, D., Walters, W.A., Knight, R., Sinha, R., Gilroy, E., Gupta, K., Baldassano, R., Nessel, L., Li, H., Bushman, F.D., & ,Lewis, J.D.; Linking Long-Term Dietary Patterns with Gut Microbial Enterotypes, Science. 2011 October 7; 334(6052): 105–108. doi:10.1126/science.1208344

Zhang, Q., Wu, Y., & Fei, X.; Effect of probiotics on body weight and body-mass index: a systematic review and meta-analysis of randomized, controlled trials, Int J Food Sci Nutr 2016:1-10, Pages 571-580, http://dx.doi.org/10.1080/09637486.2016.1181156

Zhang, Q., Wu, Y., & Fei, X.; Effect of probiotics on glucose metabolism in patients with type 2 diabetes mellitus: A meta-analysis of randomized controlled trials, Medicina 2016;52:28-34, http://dx.doi.org/10.1016/j.medici.2015.11.008

19 APPENDICES

Appendix I: Sucralose

山东康宝生化科技有限公司
Shandong Kanbo Biochemical Technology Co., Ltd
Liba Rd. #1016, Lijin, Dongying, Shandong, China - 257400

Sucralose Specification

Updated on Dec.20th, 2016

Product Name: Sucralose Manufacturer: Shandong Kanbo Origin: China
Formula: $C_{12}H_{19}Cl_3O_8$ CAS: 【56038-13-2】 Molecular Weight: 397.64

Description:
Sucralose is white to off white, crystalline powder. It is freely soluble in water, methanol, and alcohol. It is slightly soluble in ethyl acetate. It functions as a nonnutritive sweetener/flavor enhancer. Kanbo sucralose has a clean, sweet taste that is approximately 600 times sweeter than sugar. This material is Kosher, BRC and Halal certified. The product complies with FCC9, EP7.0, USP38 and E955 standards, according to Commission Regulation (EU) No 231/2012.
Suggested uses/applications:
Tabletops, tablets, pharmaceutical, powdered drink mixes, baking, carbonated soft drinks, yogurts, ice cream.
Shelf Life: 2 years
Storage Conditions: Store in a cool, dry place, away from high temperature and direct sunlight.

Items	Specification	Test Method
Appearance	White to off-white crystalline powder.	FCC 9
Identification	The infrared absorption spectrum of a potassium bromide dispersion of the sample exhibits relative maxima at the same wavelengths as those of a similar preparation of Sucralose Standard for analytical use.	FCC 9
Assay	98.0% ~102.0%	FCC 9
Specific Rotation	+84.0°~ +87.5°	FCC 9
Moisture	Max. 2.0%	FCC 9
pH value (10%solution)	5.0-7.0	KB-B-501
Residue on ignition	Max.0.7%	FCC 9
Hydrolysis products	Pass	FCC 9
Methanol	Max.0.1%	FCC 9
Lead	Max.1 mg/kg	FCC 9
Arsenic(As)	Max.3 mg/kg	GB/T5009.76
Heavy Metals	Max.10 mg/kg	GB/T5009.74
Other chlorinated disaccharides	Max.0.5%	E955
Triphenylphosphine oxide	Max.150 mg/kg	E955

1 / 2

Chlorinated monosaccharides	Max.0.1%	E955
Total aerobic count	Max.250cfu/g	GB4789.2
Yeast & Moulds	Max.50cfu/g	GB4789.15
Coliforms	negative	GB4789.3
E.coli	negative	GB4789.38
S.aureus	negative	GB4789.10
Salmonella	negative	GB4789.4
Conclusion	Kanbo Sucralose is in conformity with the FCC 9.	

Mr. Liu Shilong

Quality Control Manager

Shandong Kanbo Biochemical Technology Co., Ltd

Address: Liba Rd. #1016, Lijin, Dongying, Shandong Province, China - 257400

Tel: +86 546 6092777

Fax: +86 546 5368 777

Email: shilong.liu@kanbosweet.com

Appendix II: Aroma Panna Dry

microcapsule®

essepi

SOTTERI & PROSPERO s.r.l.

Cap. Soc. Int. Vers. € 10.400
Sede Legale: **20161 Milano - Via Bellerio 35**
Uffici e Stab.: **20032 Cormano (MI) - Via Bizzozzero 104/A**
Telef. 02/66302866 - Fax 02/66302766
REA Milano [illegible] Registro Imprese Trib. MI Soc. [illegible]
P. IVA - Codice Fiscale [illegible] - CC Postale [illegible]

TECHNICAL SHEET

Trade-name: AROMA PANNA DRY

Code number: 2030/12-1

DESCRIPTION
Flavour obtained for mixing of flavouring substances

APPARECE
Fine powder white/straw yellow, with smell and taste charateristic

DESTINATION
Pharmaceutical industry

COMPOSITION
Flavouring substance
Flavouring preparations

SUPPORT/CARRIERS
Dextrose
Magnesium carbonate E504 (2% max)

ADDITIVES
=====

LIMITATIONS (All.III - Reg. CE 1334/2008)
=====

LIMITATIONS (Art.6 – All. VI)
=====

LIMITATIONS (Art.6 – All. VII)
=====

SPECIFICATIONS
Specific weight: 0.3000/0.7000
Humidity: 0.0000/5.0000

SOLUBILITY
Complete in water with light sediment

LEGAL STATUS (Reg. CE 1334/2008)
Flavour

DOSAGE (approx.)
1/1000

SHELF-LIFE
In the unopened original packing, stored in cool and dry conditions, its shelf-life is 1 year.

Cormano (Mi), 16/01/2018

Appendix III: Maltodextrin

ROQUETTE
Offering the best of nature™

SPECIFICATIONS Ref: G81-225L50

PAGE 1/2

GLUCIDEX® IT 19

DEFINITION :

MALTODEXTRIN (EP) - MALTODEXTRIN (NF)
Mixture of glucose, disaccharides and polysaccharides, obtained by the partial hydrolysis of starch.
CAS n° : 9050-36-6
INCI : MALTODEXTRIN

SPECIFICATIONS :

A) CHARACTERS

APPEARANCE	White or almost white, slightly hygroscopic powder or granules.
SOLUBILITY	Freely soluble in water.

B) IDENTIFICATION

IDENTIFICATION TEST-A (*)	EP	Complies.
IDENTIFICATION TEST-B (*)	EP	Complies.
IDENTIFICATION TEST-C	EP	Complies.
IDENTIFICATION TEST-D	EP	Complies.

C) TESTS

pH	EP / USP-NF	4.0 to 7.0
SULFUR DIOXIDE	EP	20 ppm max.
SULFUR DIOXIDE	USP-NF	40 ppm max.
LOSS ON DRYING	EP / USP-NF	6.0 % max.
SULFATED ASH (*)	EP	0.5 % max.
RESIDUE ON IGNITION (*)	USP/NF	0.5 % max.
DEXTROSE EQUIVALENT	EP - USP/NF	18 - 20
PROTEIN CONTENT (*)	USP/NF	0.1 % max.
PARTICLE SIZE (sieve):		
- RESIDUE ON 500 MIC.		5 % max.
- RESIDUE ON 40 MIC.		95 % min.

* Compliance data - Tests not performed
** Monitoring plan

QUALITY ASSURANCE / PHARMA COSMETICS++++ April 1, 2019

ROQUETTE ITALIA - Società per Azioni con Socio Unico - Roquette Frères SA (FRANCIA)
SEDE LEGALE, DIREZIONE E STABILIMENTO: 15063 CASSANO SPINOLA (AL) - VIA SERRAVALLE, 26
TELEFONO: 0143 774 1 r.a., TELEX: 210161 ROQUET I, TELEFAX: 0143 477 295
CAPITALE SOCIALE INT. VERS. € 5.165.000 - CODICE FISCALE PARTITA IVA N. 00161980065 - REG. IMP. N 242/27/275
TRIBUNALE DI TORTONA - C.C.I.A.A ALESSANDRIA N. 73302

Distributed by GIUSTO FARAVELLI Spa - Italy CODE 395700

ROQUETTE
Offering the best of nature

SPECIFICATIONS

Ref: G81-225L50

PAGE 2/2

GLUCIDEX® IT 19

MICROBIOLOGICAL VALUES:

- TOTAL AEROBIC MICROBIAL COUNT	1000 cfu/g max.
- TOTAL YEASTS AND MOULDS COUNT	100 cfu/g max.
- ESCHERICHIA COLI(**)	Not detected in 10g
- SALMONELLAE(**)	Not detected in 10g

TYPICAL VALUES :

Poured bulk density: 400 g/L approx.
Particle mean diameter: 230 microns

COMMENTS :

Methods used by Roquette may be the Pharmacopoeia methods or alternative validated methods which have been compared to the Pharmacopoeia methods.

CONFORMITY :

Conforms to the current edition of:
- European Pharmacopoeia (EP)
- National Formulary (NF)

Please contact us for any statement regarding compliance to the General Chapters (elemental impurities, residual solvents, organic volatile impurities, metal catalyst, metal reagent).

STORAGE :

We recommend to preserve the product in its unopened original packaging, preferably protected from wide variations in temperature and humidity.

Expiry date : Manufacturing date + 5 years, in its unopened packaging.

* Compliance data - Tests not performed
** Monitoring plan

QUALITY ASSURANCE / PHARMA COSMETICS++++ April 1, 2019

Distributed by GIUSTO FARAVELLI Spa - Italy CODE 395700

ROQUETTE ITALIA - Società per Azioni con Socio Unico - Roquette Frères SA (FRANCIA)
SEDE LEGALE, DIREZIONE E STABILIMENTO: 15063 CASSANO SPINOLA (AL) - VIA SERRAVALLE, 26
TELEFONO: 0143 774 1 r.a., TELEX: 210161 ROQUET I, TELEFAX: 0143 477 295
CAPITALE SOCIALE INT. VERS. € 5.165.000 - CODICE FISCALE PARTITA IVA N. 00161980065 - REG. IMP. N 242/27/275
TRIBUNALE DI TORTONA - C.C.I.A.A. ALESSANDRIA N. 73302

Appendix IV: DECLARATION OF HELSINKI

WMA Declaration of Helsinki - Ethical Principles for Medical Research Involving Human Subjects

Adopted by the 18th WMA General Assembly, Helsinki, Finland, June 1964
and amended by the:
29th WMA General Assembly, Tokyo, Japan, October 1975
35th WMA General Assembly, Venice, Italy, October 1983
41st WMA General Assembly, Hong Kong, September 1989
48th WMA General Assembly, Somerset West, Republic of South Africa, October 1996
52nd WMA General Assembly, Edinburgh, Scotland, October 2000
53rd WMA General Assembly, Washington DC, USA, October 2002 (Note of Clarification added)
55th WMA General Assembly, Tokyo, Japan, October 2004 (Note of Clarification added)
59th WMA General Assembly, Seoul, Republic of Korea, October 2008
64th WMA General Assembly, Fortaleza, Brazil, October 2013

Preamble

1. The World Medical Association (WMA) has developed the Declaration of Helsinki as a statement of ethical principles for medical research involving human subjects, including research on identifiable human material and data.

The Declaration is intended to be read as a whole and each of its constituent paragraphs should be applied with consideration of all other relevant paragraphs.

2. Consistent with the mandate of the WMA, the Declaration is addressed primarily to physicians. The WMA encourages others who are involved in medical research involving human subjects to adopt these principles.

General Principles

3. The Declaration of Geneva of the WMA binds the physician with the words,

“The health of my patient will be my first consideration,” and the International Code of Medical Ethics declares that, “A physician shall act in the patient's best interest when providing medical care.”

4. It is the duty of the physician to promote and safeguard the health, well-being and rights of patients, including those who are involved in medical research. The physician's knowledge and conscience are dedicated to the fulfilment of this duty.

5. Medical progress is based on research that ultimately must include studies involving human subjects.

6. The primary purpose of medical research involving human subjects is to understand the causes, development and effects of diseases and improve preventive, diagnostic and therapeutic interventions (methods, procedures and treatments). Even the best proven interventions must be evaluated continually through research for their safety, effectiveness, efficiency, accessibility and quality.

7. Medical research is subject to ethical standards that promote and ensure respect for all human subjects and protect their health and rights.

8. While the primary purpose of medical research is to generate new knowledge, this goal can never take precedence over the rights and interests of individual research subjects.

9. It is the duty of physicians who are involved in medical research to protect the life, health, dignity, integrity, right to self-determination, privacy, and confidentiality of personal information of research subjects. The responsibility for the protection of research subjects must always rest with the physician or other health care professionals and never with the research subjects, even though they have given consent.

10. Physicians must consider the ethical, legal and regulatory norms and standards for research involving human subjects in their own countries as well as applicable international norms and standards. No national or international ethical, legal or regulatory requirement should reduce or eliminate any of the protections for research subjects set forth in this Declaration.

11. Medical research should be conducted in a manner that minimises possible harm to the environment.

12. Medical research involving human subjects must be conducted only by

individuals with the appropriate ethics and scientific education, training and qualifications. Research on patients or healthy volunteers requires the supervision of a competent and appropriately qualified physician or other health care professional.

13. Groups that are underrepresented in medical research should be provided appropriate access to participation in research.

14. Physicians who combine medical research with medical care should involve their patients in research only to the extent that this is justified by its potential preventive, diagnostic or therapeutic value and if the physician has good reason to believe that participation in the research study will not adversely affect the health of the patients who serve as research subjects.

15. Appropriate compensation and treatment for subjects who are harmed as a result of participating in research must be ensured.

Risks, Burdens and Benefits

16. In medical practice and in medical research, most interventions involve risks and burdens.

Medical research involving human subjects may only be conducted if the importance of the objective outweighs the risks and burdens to the research subjects.

17. All medical research involving human subjects must be preceded by careful assessment of predictable risks and burdens to the individuals and groups involved in the research in comparison with foreseeable benefits to them and to other individuals or groups affected by the condition under investigation.

Measures to minimise the risks must be implemented. The risks must be continuously monitored, assessed and documented by the researcher.

18. Physicians may not be involved in a research study involving human subjects unless they are confident that the risks have been adequately assessed and can be satisfactorily managed.

When the risks are found to outweigh the potential benefits or when there is conclusive proof of definitive outcomes, physicians must assess whether to continue, modify or immediately stop the study.

Vulnerable Groups and Individuals

19. Some groups and individuals are particularly vulnerable and may have an increased likelihood of being wronged or of incurring additional harm.

All vulnerable groups and individuals should receive specifically considered protection.

20. Medical research with a vulnerable group is only justified if the research is responsive to the health needs or priorities of this group and the research cannot be carried out in a non-vulnerable group. In addition, this group should stand to benefit from the knowledge, practices or interventions that result from the research.

Scientific Requirements and Research Protocols

21. Medical research involving human subjects must conform to generally accepted scientific principles, be based on a thorough knowledge of the scientific literature, other relevant sources of information, and adequate laboratory and, as appropriate, animal experimentation. The welfare of animals used for research must be respected.

22. The design and performance of each research study involving human subjects must be clearly described and justified in a research protocol.

The protocol should contain a statement of the ethical considerations involved and should indicate how the principles in this Declaration have been addressed. The protocol should include information regarding funding, sponsors, institutional affiliations, potential conflicts of interest, incentives for subjects and information regarding provisions for treating and/or compensating subjects who are harmed as a consequence of participation in the research study.

In clinical trials, the protocol must also describe appropriate arrangements for post-trial provisions.

Research Ethics Committees

23. The research protocol must be submitted for consideration, comment, guidance and approval to the concerned research ethics committee before the study begins. This committee must be transparent in its functioning, must be independent of the researcher, the sponsor and any other undue influence and must be duly qualified. It must take into consideration the laws and regulations of the country or countries in which the research is to be performed as well as applicable international norms and

standards but these must not be allowed to reduce or eliminate any of the protections for research subjects set forth in this Declaration.

The committee must have the right to monitor ongoing studies. The researcher must provide monitoring information to the committee, especially information about any serious adverse events. No amendment to the protocol may be made without consideration and approval by the committee. After the end of the study, the researchers must submit a final report to the committee containing a summary of the study's findings and conclusions.

Privacy and Confidentiality

24. Every precaution must be taken to protect the privacy of research subjects and the confidentiality of their personal information.

Informed Consent

25. Participation by individuals capable of giving informed consent as subjects in medical research must be voluntary. Although it may be appropriate to consult family members or community leaders, no individual capable of giving informed consent may be enrolled in a research study unless he or she freely agrees.

26. In medical research involving human subjects capable of giving informed consent, each potential subject must be adequately informed of the aims, methods, sources of funding, any possible conflicts of interest, institutional affiliations of the researcher, the anticipated benefits and potential risks of the study and the discomfort it may entail, post-study provisions and any other relevant aspects of the study. The potential subject must be informed of the right to refuse to participate in the study or to withdraw consent to participate at any time without reprisal. Special attention should be given to the specific information needs of individual potential subjects as well as to the methods used to deliver the information.

After ensuring that the potential subject has understood the information, the physician or another appropriately qualified individual must then seek the potential subject's freely-given informed consent, preferably in writing. If the consent cannot be expressed in writing, the non-written consent must be formally documented and witnessed.

All medical research subjects should be given the option of being informed about the general outcome and results of the study.

27. When seeking informed consent for participation in a research study the physician must be particularly cautious if the potential subject is in a dependent relationship with the physician or may consent under duress. In such situations the informed consent must be sought by an appropriately qualified individual who is completely independent of this relationship.

28. For a potential research subject who is incapable of giving informed consent, the physician must seek informed consent from the legally authorised representative. These individuals must not be included in a research study that has no likelihood of benefit for them unless it is intended to promote the health of the group represented by the potential subject, the research cannot instead be performed with persons capable of providing informed consent, and the research entails only minimal risk and minimal burden.

29. When a potential research subject who is deemed incapable of giving informed consent is able to give assent to decisions about participation in research, the physician must seek that assent in addition to the consent of the legally authorised representative. The potential subject’s dissent should be respected.

30. Research involving subjects who are physically or mentally incapable of giving consent, for example, unconscious patients, may be done only if the physical or mental condition that prevents giving informed consent is a necessary characteristic of the research group. In such circumstances the physician must seek informed consent from the legally authorised representative. If no such representative is available and if the research cannot be delayed, the study may proceed without informed consent provided that the specific reasons for involving subjects with a condition that renders them unable to give informed consent have been stated in the research protocol and the study has been approved by a research ethics committee. Consent to remain in the research must be obtained as soon as possible from the subject or a legally authorised representative.

31. The physician must fully inform the patient which aspects of their care are related to the research. The refusal of a patient to participate in a study or the patient’s decision to withdraw from the study must never adversely affect the patient-physician relationship.

32. For medical research using identifiable human material or data, such as research on material or data contained in biobanks or similar repositories, physicians must seek informed consent for its collection, storage and/or reuse. There may be exceptional situations where consent would be impossible or impracticable to obtain

6/8

for such research. In such situations the research may be done only after consideration and approval of a research ethics committee.

Use of Placebo

33. The benefits, risks, burdens and effectiveness of a new intervention must be tested against those of the best proven intervention(s), except in the following circumstances:

Where no proven intervention exists, the use of placebo, or no intervention, is acceptable; or

Where for compelling and scientifically sound methodological reasons the use of any intervention less effective than the best proven one, the use of placebo, or no intervention is necessary to determine the efficacy or safety of an intervention

and the patients who receive any intervention less effective than the best proven one, placebo, or no intervention will not be subject to additional risks of serious or irreversible harm as a result of not receiving the best proven intervention.

Extreme care must be taken to avoid abuse of this option.

Post-Trial Provisions

34. In advance of a clinical trial, sponsors, researchers and host country governments should make provisions for post-trial access for all participants who still need an intervention identified as beneficial in the trial. This information must also be disclosed to participants during the informed consent process.

Research Registration and Publication and Dissemination of Results

35. Every research study involving human subjects must be registered in a publicly accessible database before recruitment of the first subject.

36. Researchers, authors, sponsors, editors and publishers all have ethical obligations with regard to the publication and dissemination of the results of research. Researchers have a duty to make publicly available the results of their research on human subjects and are accountable for the completeness and accuracy of their reports. All parties should adhere to accepted guidelines for ethical reporting. Negative and inconclusive as well as positive results must be published or otherwise made

publicly available. Sources of funding, institutional affiliations and conflicts of interest must be declared in the publication. Reports of research not in accordance with the principles of this Declaration should not be accepted for publication.

Unproven Interventions in Clinical Practice

37. In the treatment of an individual patient, where proven interventions do not exist or other known interventions have been ineffective, the physician, after seeking expert advice, with informed consent from the patient or a legally authorised representative, may use an unproven intervention if in the physician's judgement it offers hope of saving life, re-establishing health or alleviating suffering. This intervention should subsequently be made the object of research, designed to evaluate its safety and efficacy. In all cases, new information must be recorded and, where appropriate, made publicly available.

© World Medical Association, Inc. - All Rights reserved.
© Asociación médica mundial - Todos los derechos reservados.
© L'Association Médicale Mondiale - Tous droits réservés.

Appendix V: SERIOUS ADVERSE EVENT FORM

Sponsor: Slimbiotics GmbH	Serious Adverse Event Report Form	Study Code: Slim-LfX2-2021

1. Subject details

Subject ID No.	Sex ☐ M ☐ F	Age (years)	Height (cm)	Weight (kg)	SAE No.

INVESTIGATOR: Dr. Christiane Laue<br>INSTITUTION: CRC Kiel GmbH<br>Date and time investigator became aware of the SAE:<br>Date: |__|__|/|__|__|/|__|__|__|__| (DD/MM/YYYY)<br>Time: |__|__| : |__|__| (24 hr clock)	ADDRESS: Schauenburgerstr. 116 D-24118 Kiel /Germany<br>PHONE: +49(0)431-5606-599<br>FAX: +49(0)431-5606-596<br>E-MAIL: c.laue@crc-kiel.de	**REPORT INFORMATION**<br>☐ INITIAL REPORT<br>Date: |__|__|/|__|__|/|__|__|__|__| (DD/MM/YYYY)<br>☐ FOLLOW-UP<br>Date: |__|__|/|__|__|/|__|__|__|__| (DD/MM/YYYY)

2. Serious adverse event

SERIOUSNESS CRITERIA (check all that apply)	OUTCOME OF SAE (at time of report)
☐ Death<br>☐ Life threatening<br>☐ Hospitalization (initial)<br>☐ Hospitalization (prolonged)<br>☐ Congenital abnormality / Birth defect<br>☐ Persistent or significant disability / incapacity<br>☐ Medically significant	☐ Ongoing<br>☐ Resolved (no sequelae)<br>☐ Resolved (with sequelae)<br>☐ Recovering<br>☐ Death (cause of death: _______________)<br>Autopsy? ☐ yes ☐ no<br>☐ lost to follow-up (unknown?)

**DIAGNOSIS INCLUDING SYMPTOMS:**	
____________________<br>____________________<br>____________________<br>____________________	**Date and time of start (onset):**<br>Date: |__|__|/|__|__|/|__|__|__|__| (DD/MM/YYYY) Time: |__|__|:|__|__| (24 hr clock)<br>**Date and time of end (ceased):**<br>Date: |__|__|/|__|__|/|__|__|__|__| (DD/MM/YYYY) Time: |__|__|:|__|__| (24 hr clock)<br>**Date and time of death:**<br>Date: |__|__|/|__|__|/|__|__|__|__| (DD/MM/YYYY) Time: |__|__|:|__|__| (24 hr clock)

SEVERITY (Intensity grade): ☐ mild ☐ moderate ☐ severe

3. Test Product **UNBLINDING:** ☐ not applicable ☐ no ☐ yes

Product Name:	Date of first consumption (DD/MM/YYYY)	Time interval between last consumption and start of event	Daily dose, route of administration

Relationship to test product:
☐ not applicable ☐ certain ☐ probable ☐ possible ☐ unlikely ☐ not related ☐ not assessable

SAE Form_Slim-LfX2-2021_Version 1.0_30.04.2021.docx Seite 1 von 3

**Sponsor:** Slimbiotics GmbH	**Serious Adverse Event Report Form**	**Study Code:** Slim-LfX2-2021

**Subject details**			
Subject ID No.	Age (years)	SAE No.	☐ INITIAL REPORT Date: |__|__|/|__|__|/|__|__|__|__| DD/MM/YYYY)<br>☐ FOLLOW-UP Date: |__|__|/|__|__|/|__|__|__|__| (DD/MM/YYYY)

4. Medical history relevant to SAE	Start date	Stop date
**1.**		
**2.**		
**3.**		
**4.**		
**5.**		

5. Concomitant medication relevant to SAE

	Indication	Daily dose, unit, route of administration	Date of first administration	Date of last administration
**1.**				
**2.**				
**3.**				
**4.**				

6. Lab findings or investigations (enter only those findings necessary for SAE diagnosis)

	Normal range	Date	Result
**1.**			
**2.**			
**3.**			
**4.**			

7. Action taken

REGARDING TREATMENT OF SAE

☐ none

☐ drug treatment

☐ others

Specify:

____

____

____

REGARDING TEST PRODUCT

☐ dose not changed

☐ dose reduced

☐ temporary interrupted

(date of last intake) (date of continuation)

☐ test product discontinued

(date of last intake)

☐ unknown

☐ not applicable

**Sponsor:** Slimbiotics GmbH	**Serious Adverse Event Report Form**	**Study Code:** Slim-LfX2-2021

**Subject details**			
Subject ID No.	Age (years)	SAE No.	☐ INITIAL REPORT Date: |__|__|/|__|__|/|__|__|__|__| DD/MM/YYYY)<br>☐ FOLLOW-UP Date: |__|__|/|__|__|/|__|__|__|__| (DD/MM/YYYY)
**8. Additional information**			
**9. Investigator details**			
Name of investigator or reporting person:			
**Signature of investigator or reporting person**			**Date (DD/MM/YYYY)**

FAX WITHIN 24 hrs to, Slimbiotics GmbH, FAX No.: +43/1/235 07 90 and Email: knaipp-linnerth@slimbiotics.com

SAE Form_Slim-LfX2-2021_Version 1.0_30.04.2021.docx Seite 3 von 3

20 SIGNATURES

SPONSOR:

SLIMBIOTICS GMBH
Tuchlauben 18/12
A - 1010 Vienna, Austria

DATE……………………….. SIGNATURE………………………..

PRINCIPAL INVESTIGATOR:

Dr. med. Christiane Laue

Clinical Research Center (CRC) Kiel GmbH

Kiel Centre of Innovation and Technology

Schauenburgerstr. 116,

D-24118 Kiel, Germany

DATE……………………….. SIGNATURE………………………..
